# Defect‐Passivating and Dense Indolocarbazole‐Based Self‐Assembled Monolayers for Efficient Inverted Perovskite Solar Cells With over 26.1% Efficiency

**DOI:** 10.1002/smll.202512942

**Published:** 2026-01-19

**Authors:** Xu Fu, Yuxuan Yang, Dingqian He, Peng Zhao, Huixin Gao, Zhen Zhu, Yi Zhang, Bao Zhang, Mohammad Khaja Nazeeruddin

**Affiliations:** ^1^ School of Chemical Engineering and Technology Tianjin University Tianjin P. R. China; ^2^ Collaborative Innovation Center of Chemical Science and Engineering Tianjin University Tianjin P. R. China; ^3^ School of Integrated Circuits Southeast University Wuxi Jiangsu P. R. China; ^4^ Institute of Molecular Plus School of Chemical Engineering and Technology Tianjin University Tianjin P. R. China

**Keywords:** hole‐transporting layers, indolocarbazole, nickel oxide, perovskite solar cells, self‐assembled monolayers

## Abstract

Self‐assembled monolayers (SAMs) are widely used in NiO_x_‐based inverted perovskite solar cells (PSCs). However, the poor wettability, low surface coverage, inhomogeneous distribution, and inadequate defect passivation capability of SAMs limit further improvements in the efficiency and stability of IPSCs. Herein, we have developed a series of indolocarbazole‐based SAMs, namely, D3PAICz‐1, M3PAICz‐1, D3PAICz‐2, and M3PAICz‐2. We systematically investigated how the nitrogen positions in indolocarbazole‐based SAMs and the anchoring group number synergistically influence device performance and stability. Among these SAMs, monophosphonate‐anchored M3PAICz‐1 demonstrates advantages in surface wettability, compactness, film uniformity, energy level alignment, hole extraction, and defect passivation. Consequently, the corresponding 1.55 eV bandgap PSCs achieved the highest power conversion efficiency (PCE) of 26.12% and excellent air stability. More interestingly, M3PAICz‐1 also endowed 1.68 eV wide‐bandgap (WBG) PSCs with an impressive efficiency of 22.19%, demonstrating its good universality as HTL. Our findings provide valuable insights for designing novel SAM molecules targeted at practical high‐performance PSCs.

## Graphical abstract

This study proposes a strategy to modulate nitrogen positions (meta‐/para‐) in indolocarbazole units and the number of anchoring groups (mono‐/bis‐phosphonate), achieving a remarkable PCE of 26.12%, among the highest reported for PSCs using indolocarbazole‐based SAMs as HTLs.

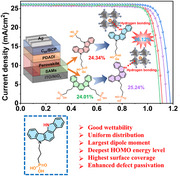

## 1 Introduction

Perovskite solar cells are now regarded as a leading contender in the field of photovoltaics for the unprecedented progress in power conversion efficiency in recent years [1, 2, 3, 4, 5, 6, 7]. The reported PCE values for laboratory‐scale PSCs have already approached 27.0% [8, 9, 10, 11], gradually approaching the theoretical limit. The inverted (p‐i‐n) PSCs (IPSCs) possess several advantages over regular (n‐i‐p) PSCs, including low‐temperature processing, superior stability, and negligible hysteresis, as well as their compatibility with the commercially mature c‐Si PV technology for tandem devices [12, 13, 14, 15].

As an efficient interface design in IPSCs, the double HTLs composed of NiO_x_/SAM could minimize interfacial reactions between perovskite and Ni^3+^, enhance hole extraction, and suppress non‐radiative recombination [16, 17, 18, 19, 20, 21]. However, commercially used SAMs, such as [2‐(9H‐carbazol‐9‐yl)ethyl]phosphonic acid (2PACz), suffer from low surface coverage, inhomogeneous distribution, poor wettability to perovskite precursors, and inadequate defect passivation [22, 23, 24, 25, 26]. These disadvantages challenge the deposition of a high‐quality perovskite layer on substrates and result in low fabrication yield as well as undesired interfacial losses, thereby limiting PSC performance and stability [27, 28, 29, 30]. Compared to carbazole‐based SAMs, SAMs incorporating the indolocarbazole (ICz) unit exhibit stronger intermolecular interactions, promoting molecular ordering during self‐assembly and enabling the formation of a compact SAM layer [31, 32]. Moreover, the extended *π*‐conjugation in ICz‐based SAMs also enhances charge transport, making them promising HTLs for IPSCs [33, 34, 35]. Wu et al. developed a series of bisphosphonate‐anchored ICz‐based SAMs by tuning the nitrogen positions in the ICz unit to enhance molecular dipole moments and *π*–*π* interactions, achieving an impressive PCE of 25.15% for IDCz‐3‐based IPSC, which is a record efficiency for multipodal SAMs‐based PSCs [36]. Lan et al. developed a monophosphonate‐anchored SAM (2PICz) based on indolo[3,2‐b]carbazole. Notably, when 2PICz was employed as the HTL, the corresponding PSC attained a PCE of 25.51% [37], significantly exceeding that of devices using the bisphosphonate‐anchored counterpart IDCz‐1 (20.97%). However, they did not investigate how the exposed N─H of 2PICz specifically affects the performance of PSCs. Even though previous studies have demonstrated that the indolocarbazole unit is a promising template for the development of efficient SAMs [38], how the interplay between the position of the two nitrogen atoms and the number of anchoring groups (mono or multipodal) influences PSC device performance and stability remains unknown.

**FIGURE 1:**
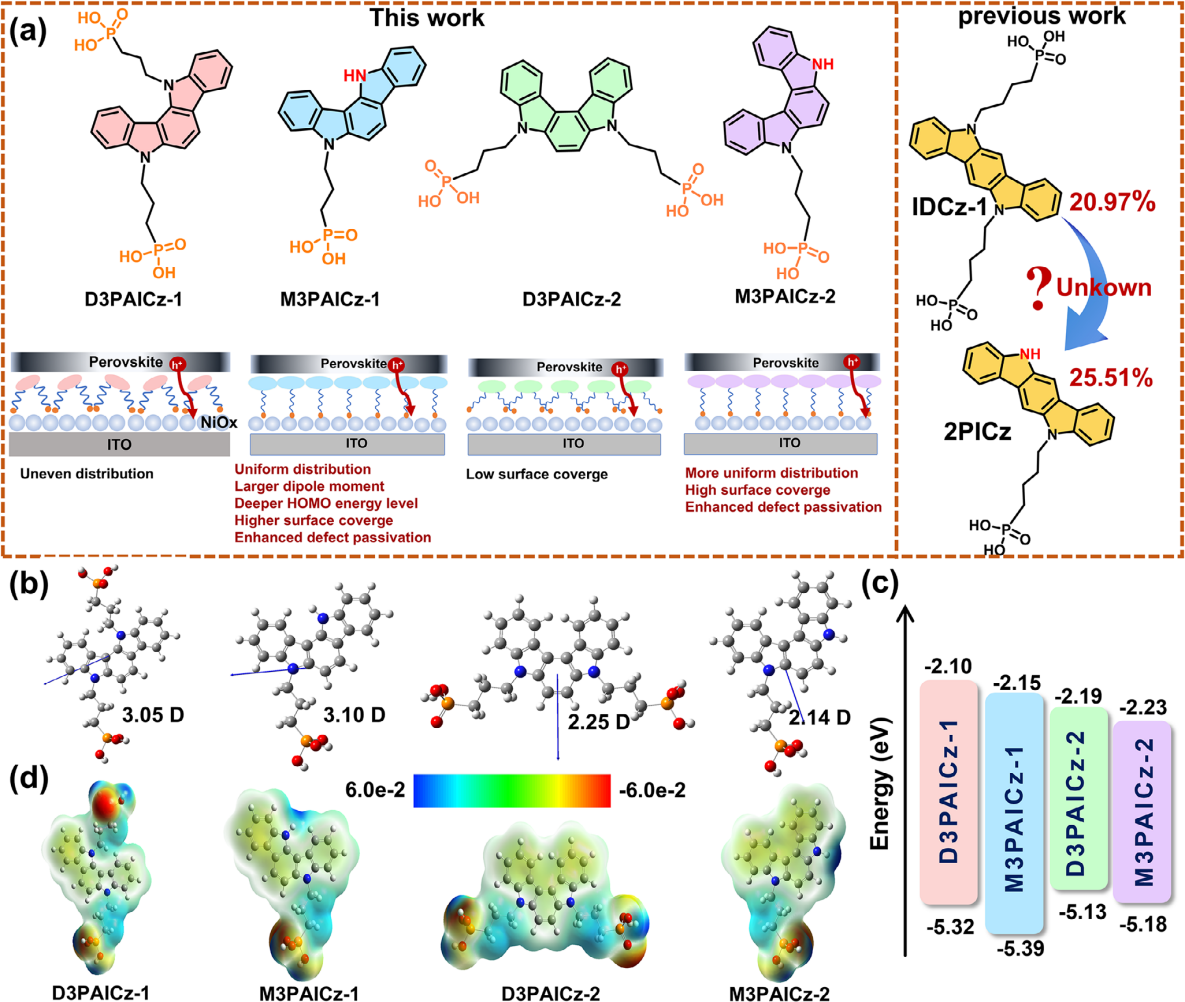
(a) Molecular design and structures. (b) Dipole moments, (c) Energy levels, (d) ESP mapping of the indolocarbazole‐derived SAMs.

Herein, we developed a class of ICz‐derived SAMs involving bisphosphonate (D) and/or monophosphonate (M) anchoring groups, namely, D3PAICz‐1, M3PAICz‐1, D3PAICz‐2, and M3PAICz‐2 (Figure 1a). The regulation of dipole moment, the highest occupied molecular orbital energy level, and compactness of the SAMs could be realized by adjusting the position (meta‐, para‐) of two nitrogen atoms in ICz units. Importantly, we revealed the mechanism by which the number of anchoring groups of ICz‐based SAM molecules affects the surface coverage and film uniformity on the substrate, and the ability of defect passivation. Among these SAMs, meta‐M3PAICz‐1 with a single anchoring group exhibits the largest dipole moment and the highest HOMO energy level, which facilitates efficient hole extraction. M3PAICz‐1 also forms the densest film on NiO_x_, thereby improving interfacial contact and suppressing non‐radiative recombination. Moreover, M3PAICz‐1 passivates the perovskite most effectively by the formation of a hydrogen bond between the exposed N‐H site and I^−^ ions, consistent with its highest binding energy to the perovskite surface. Consequently, the champion PCE of 26.12% is achieved in 1.55 eV bandgap IPSCs using NiO_x_/M3PAICz‐1 as the HTL, which is among the highest for IPSCs. The corresponding 1.68 eV wide‐bandgap IPSCs also obtained an impressive PCE of 22.19%, demonstrating the universality of M3PAICz‐1 as HTL. Moreover, the stability of IPSCs with M3PAICz‐1 was significantly improved, with the PCE retaining 87.3% of its original value after 1000 h of storage in ambient air (20 ± 5°C, 35 ± 5% RH).

## 2 Results and Discussion

The synthetic routes and detailed procedures for the SAMs derived from 5,12‐dihydroindolo[3,2‐a]carbazole (5,12‐ICz) and 5,8‐dihydroindolo[2,3‐c]carbazole (5,8‐ICz) are presented in Scheme S1 and Supporting Information. Comprehensive characterization, including ^1^H, ^13^C, and ^31^P NMR spectroscopy as well as high‐resolution mass spectrometry (HRMS), was employed to confirm the chemical structures of the SAMs. Thermogravimetric analysis (TGA) in Figure S27 indicates sufficient thermal stability of the SAMs, while NMR spectra (Figure S28) confirm their stability under UV light irradiation.

The optical bandgap (*E_g_*) of these SAMs was estimated from UV–vis absorption spectra (Figure S29a). Moreover, these SAMs exhibit negligible parasitic absorption (Figure S29b). As shown in Figure 1d and Figure S31, the exposed N‐H sites of monophosphonate‐anchored M3PAICz‐1 and M3PAICz‐2 exhibit positive charges, enabling them to passivate I‐related defects. The perovskite precursor solution displayed a contact angle of 8.4° on bare NiO_x_, which changed to 10.6°, 11.3°, 10.3°, and 10.7° after modified with D3PAICz‐1, M3PAICz‐1, D3PAICz‐2, and M3PAICz‐2 (Figure S32). The slightly increased hydrophobicity of the NiO_x_/SAMs substrates does not affect the coverage of the perovskite film (Figure S33).

To deeply understand how the different positions of the two nitrogen atoms in the ICz units affect the *π*–*π* interactions of the four SAMs (Figure S34 and Table S1), we analyzed the single‐crystal structures of their *π*‐conjugated units, sourced from the Cambridge Crystallographic Data Centre (CCDC). The absence of anchoring groups in these structures enables a direct comparison of the intrinsic intermolecular interactions among different *π*‐expanded carbazoles, thereby eliminating the confounding effects of strong interactions from anchoring‐group‐like hydrogen bonding.

The 5,12‐ICz compound adopts a slipped *π*‐stacking arrangement, with a vertical distance of 3.17 Å and a longitudinal slip of 5.52 Å between parallel adjacent carbazole molecules, resulting in strong CH‐*π* (2.40 Å) and *π*–*π* interactions. These strong *π*–*π* interactions lead to a well‐aligned, 1D linear structure extending along the a‐axis, which in turn enables the formation of a dense and well‐ordered M3PAICz‐1 film on the NiO_x_ substrate. 5,8‐ICz exhibits a T‐shaped stacking configuration, with intermolecular distances of approximately 2.75 and 3.56 Å. Multiple CH‐*π* (2.40 Å) and *π*–*π* interactions are present. However, when the intermolecular distance is 2.75 Å, the longitudinal slip of 6.06 Å fails to provide sufficient *π*–*π* orbital overlap. Overall, the void rate of 5,8‐ICz is 3.90%, which is higher than that of 5,12‐ICz (33.05%), indicating a less compact stacking structure compared to 5,12‐ICz. The enhanced intermolecular interactions and reduced skeletal distortion of 5,12‐ICz may promote the formation of denser films during the self‐assembly processes of M3PAICz‐1 and D3PAICz‐1.

Meanwhile, the phosphonic acid‐containing SAM structure was optimized using DFT calculations with the Perdew–Burke–Ernzerhof (PBE) functional (Figure S35). The results indicate that multiple strong hydrogen bonds (1.559, 1.545, and 1.594 Å) tend to form among the phosphonic acid groups in the bisphosphonate‐anchored SAM D3PAICz‐1. Similarly, multiple strong hydrogen bonds (1.444 and 1.624 Å) were observed in D3PAICz‐2. Such strong hydrogen bonding interactions between phosphonic acid groups readily lead to SAM agglomeration in solution, affecting subsequent deposition on NiO_x_ substrates and resulting in rough NiO_x_/SAM substrates. In contrast, M3PAICz‐1 and M3PAICz‐2 possess only one phosphonic acid anchoring group. The introduction of N─H groups causes the N‐H moiety to form relatively weak hydrogen bonds with the phosphonic acid group (1.992 Å/1.894 Å/1.844 Å or 1.750 Å/1.914 Å/ 2.849 Å), significantly reducing the possibility of strong hydrogen bonds forming between phosphonic acid groups and thereby decreasing SAM agglomeration. This facilitates the formation of uniform, smooth films of SAM molecules on the NiO_x_ substrate. To determine the hydrodynamic diameter of SAM particles in ethanol solution, we performed dynamic light scattering (DLS) measurements (Figure S36). The results show that the average size of D3PAICz‐1 particles and D3PAICz‐2 particles in ethanol solution is 647 nm and 663 nm, respectively, significantly higher than the 311 and 349 nm values for M3PAICz‐1 and M3PAICz‐2. This validates our analysis of simulated intermolecular stacking. After modification with SAMs, the RMS roughness of the NiO_x_ substrate, from 4.51 nm for the bare NiO_x_ substrate, decreased to 3.76 nm for NiO_x_/D3PAICz‐1, 2.14 nm for NiO_x_/M3PAICz‐1, 3.65 nm for NiO_x_/D3PAICz‐2, and 2.96 nm for NiO_x_/M3PAICz‐2 (Figure S37). This further confirms that the reduction of phosphonic acid groups and the introduction of N─H bonds effectively mitigate aggregation effects, thereby facilitating the formation of smoother films on the NiO_x_ substrate. This reduction in RMS roughness indicates improved interfacial contact between the NiO_x_ and the perovskite [39].

To investigate the interaction between SAM molecules and the underlying NiO_x_, DFT calculations were performed to determine the binding configurations of four SAM molecules on NiO_x_ surfaces (Figure S38). Theoretical calculations revealed that both phosphonic acid groups in D3PAICz‐1 and D3PAICz‐2 can achieve coordination. Both anchoring groups of D3PAICz‐1 can react with NiO_x_ substrates via a P‐O─Ni bond and a P═O─Ni coordination bond, forming a weak didentate anchoring. For D3PAICz‐2, an anchoring group reacts with the NiO_x_ substrate via a P‐O─Ni bond and a P═O─Ni coordination bond, while the other anchoring group reacts with the NiO_x_ substrate via two P‐O─Ni bonds and a P═O─Ni coordination bond. The adsorption energies (*ΔE*_ads_) of D3PAICz‐1 and D3PAICz‐2 on NiO_x_ were calculated to be −12.39 and −13.64 eV, respectively, apparently higher than that of M3PAICz‐1 (−7.13 eV) and M3PAICz‐2 (−6.89 eV) on NiO_x_. However, both M3PAICz‐1 and M3PAICz‐2 can react with NiO_x_ substrates via two P‐O─Ni bonds and a P═O─Ni coordinate bond, forming a more robust tridentate anchoring.

**FIGURE 2:**
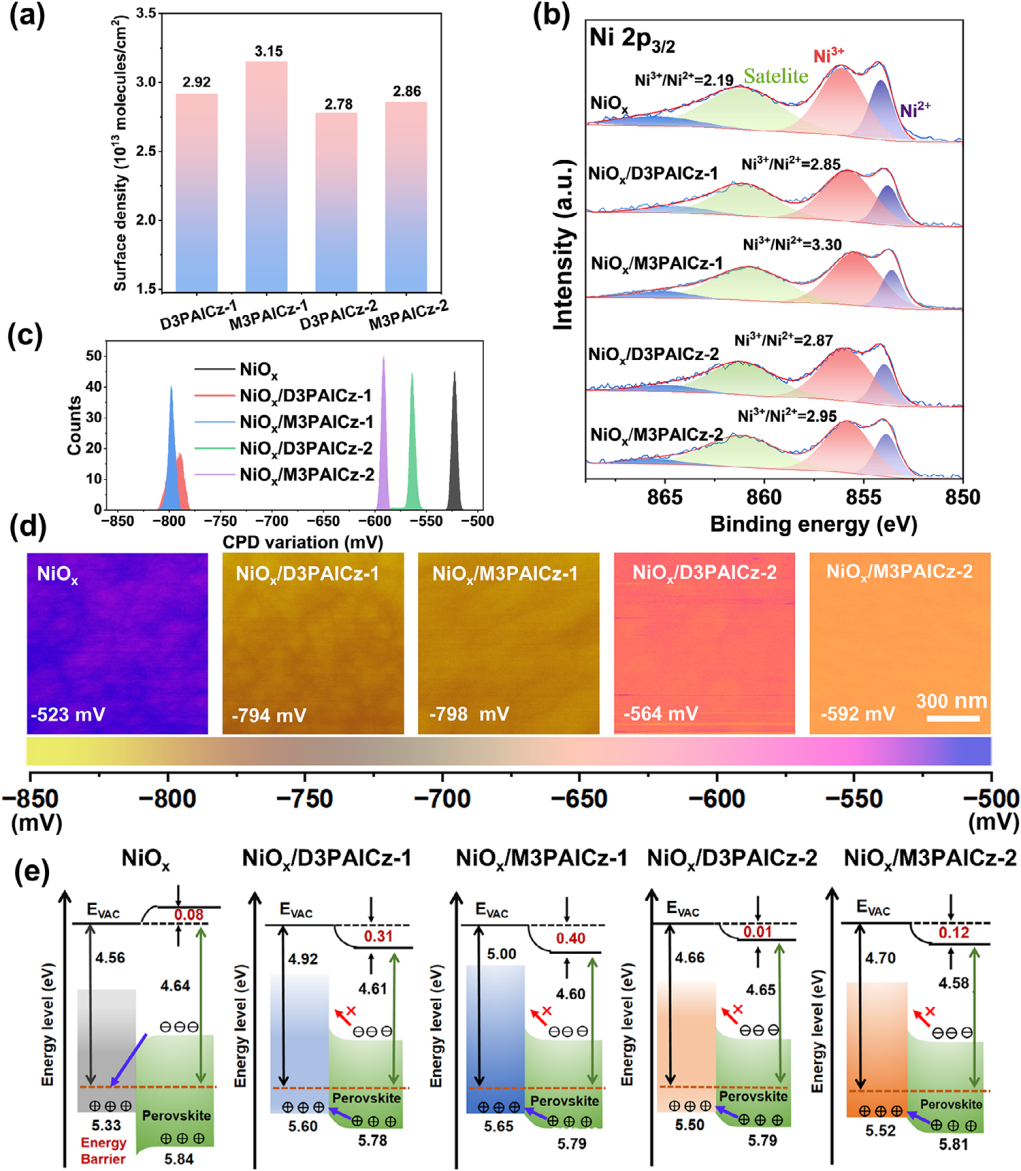
(a) The surface density of the four SAMs on the NiO_x_ surface determined by CV voltammograms. (b) Ni 2p3/2 XPS, (c) surface potential distribution, and (d) KPFM images of bare and SAM‐modified NiO_x_. (e) Energy level diagrams of NiO_x_/perovskite and NiO_x_/SAMs/perovskite.

The number and position of anchoring groups influence the surface coverage of these SAMs on NiO_x_. To quantify the actual molecular density on the NiO_x_ surface, we estimated it using both UV–vis spectroscopy and cyclic voltammetry, and validated the results with the N/Ni ratio measured by XPS. We performed UV–vis spectroscopy on NiO_x_/SAM samples before and after ethanol rinsing. Surface coverage was calculated using the formula Γ = (*A*λ)/ε(λ))/100 [40]. The surface coverage of SAM on NiO_x_ substrates before/after rinsing with ethanol was determined to be 14.97/8.62, 15.66/8.72, 14.10/7.31, and 14.73/8.42 × 10^−10^ mol cm^−2^, respectively (Figure S39). The surface coverage of M3PAICz‐1 and D3PAICz‐1 is higher than that of M3PAICz‐2 and D3PAICz‐2, confirming that the density of the SAM layer can be regulated by adjusting the position of the N atom in the ICZ unit. Simultaneously, the higher coverage of M3PAICz‐1 compared to D3PAICz‐1 and that of M3PAICz‐2 relative to D3PAICz‐2 confirms the regulatory role of N─H groups in the self‐assembly process. Additionally, we conducted cyclic voltammetry testing. The quantity of adsorbed molecules on the NiO_x_ surface was derived from the scan‐rate‐dependent oxidative peak intensity in cyclic voltammetry measurements (Note S1). The oxidation peaks observed for ITO/NiO_x_/SAM in cyclic voltammetry confirm the successful adsorption of these SAMs onto the NiO_x_ substrate. The surface density of D3PAICz‐1, M3PAICz‐1, D3PAICz‐2, and M3PAICz‐2 was determined to be 2.92 × 10^13^, 3.15 × 10^13^, 2.78 × 10^13^, and 2.86 × 10^13^ molecules cm^−2^, respectively (Figure 2a; Figure S40), consistent with the results calculated from the UV–vis spectroscopy test results. Simultaneously, we tested and analyzed the XPS data of NiO_x_/SAM samples before and after ethanol washing (Figures S41–S44). We calculated the N/Ni ratio for NiO_x_/SAM samples before and after ethanol rinsing, which more directly reflects SAM surface coverage. The results indicate that the N/Ni ratios for D3PAICz‐1, M3PAICz‐1, D3PAICz‐2, and M3PAICz‐2 before and after ethanol rinsing were 0.0619/0.0375, 0.0637/0.0387, 0.0536/0.0335, and 0.0597/0.0358, respectively (Figure S45). The N/Ni ratios are consistent with the UV–vis spectroscopy results and the SAM coverage measured by cyclic voltammetry. These results indicate that these SAMs on NiO_x_ are not strictly monolayer structures. The bilayer configuration, formed by a chemically anchored SAM beneath a disordered overlayer, was demonstrated to have super‐wetting characteristics and is beneficial for high‐quality perovskite film deposition. Thus, in our work, the SAM modification was conducted by spin‐coating the SAM molecules onto the NiO_x_ substrate, followed by device fabrication without any additional washing step.

To probe the influence of these SAMs on the surface properties of the NiO_x_ substrate, we performed X‐ray photoelectron spectroscopy (XPS) measurements. The C 1s, N 1s, P 2p, and Ni 2p3/2 XPS spectra demonstrated the anchoring of these SAMs on the NiO_x_ substrate (Figures S46 and S47). The Ni 2p3/2 XPS spectra are shown in Figure 2b, in which the 856.08 and 854.04 eV peaks represent Ni^3+^ and Ni^2+^ species in NiO_x_ film, respectively. With SAM modification, the peaks shifted to lower binding energy due to the coordination between Ni atoms and the ‐P═O moieties of the SAM molecules, and the Ni^3+^/Ni^2+^ ratio increased from NiOx (2.19) to NiO_x_/D3PAICz‐1 (2.85), NiO_x_/M3PAICz‐1 (3.30), NiO_x_/D3PAICz‐2 (2.87), and NiO_x_/M3PAICz‐2(2.95) (Table S3), contributing to the improvement of the electrical conductivity of NiO_x_ (Figure S48) [41].

After the NiO_x_ surface is modified with these SAMs, the WF of NiO_x_ will change to different degrees. In this process, the dipole moments and the highest occupied molecular orbital (HOMO) energy level of the SAMs play crucial roles. The dipole moments of these SAMs were obtained via DFT calculations (Figure 1b), the HOMO energy level was determined by cyclic voltammetry (Figure 1c; Figure S30), and the WFs of the substrate were measured using ultraviolet photoemission spectroscopy (UPS) (Figures S49 and S50). For indolocarbazole‐based SAMs, the anchoring group position exerts a more pronounced influence on both the HOMO energy level and the dipole moment, whereas the number of anchoring groups plays a relatively minor role in modulating the HOMO level but does affect the orientation of the dipole moment to some extent. Specifically, when the N atom is in the meta‐position, the asymmetric molecules D3PAICz‐1 and M3PAICz‐1 exhibit deeper HOMO energy levels and larger dipole moments. In this case, the number of anchoring groups has little effect on the dipole moment. The dipole moment of M3PAICz‐1 (3.10 D) is slightly larger than that of D3PAICz‐1 (3.05 D). The dipole moment orientation of both D3PAICz‐1 and M3PAICz‐1 is relatively distant from the anchoring groups and nearly perpendicular to them. However, the HOMO energy level measured by cyclic voltammetry shows that M3PAICz‐1 (−5.39 eV) < D3PAICz‐1 (−5.32 eV). Therefore, due to the combined effect of the deeper HOMO level and slightly larger dipole moment, M3PAICz‐1 yields a deeper work function (5.00 eV) compared to D3PAICz‐1 (4.92 eV). When the N atom is in the para‐position, D3PAICz‐2 and M3PAICz‐2 exhibit shallower HOMO energy levels and smaller dipole moments compared to D3PAICz‐1 and M3PAICz‐1, resulting in a lower substrate work function. The HOMO level of M3PAICz‐2 (−5.18 eV) is slightly deeper than that of D3PAICz‐2 (−5.13 eV). The dipole moment of D3PAICz‐2 (2.25 D) is only slightly larger than that of M3PAICz‐2 (2.14 D). However, the dipole moment orientation of D3PAICz‐2 is farther from the anchoring groups and nearly perpendicular to them, whereas the dipole moment of M3PAICz‐2 is closer to the anchoring groups and almost parallel to them, pointing toward the NiO_x_ substrate upon anchoring, which is favorable for hole extraction. Ultimately, under the synergistic influence of the HOMO energy level and dipole moment, M3PAICz‐2 yields a slightly deeper work function (4.70 eV) than D3PAICz‐2 (4.66 eV). Among these SAMs, M3PAICz‐1 shows the lowest HOMO energy level and the largest dipole moment, which facilitates increasing the work function (WF) of the NiO_x_/ M3PAICz‐1 substrate.

To further investigate the effect of SAMs on energy level alignment at the HTLs/perovskite interface, we employed a damage‐free peeling technique to obtain the bottom interfaces of perovskite films deposited on different HTLs and performed UPS measurements (Figure S51). The corresponding energy level alignment diagrams are presented in Figure 2e and Table S4. According to the band alignment of the Schottky contact, for NiO_x_/perovskite (Figure 2e), the bare NiO_x_ substrate exhibits an additional 0.08 eV energy barrier arising from perovskite's downward band bending. Conversely, SAM‐modified NiO_x_ substrates induce upward band bending in perovskite, eliminating energy barriers to enhance hole extraction and suppress electron accumulation at the bottom interface [42]. For NiO_x_/M3PAICz‐1/perovskite, the deeper work function leads to enhanced interfacial hole extraction through minimized energy shifts, thereby enhancing the open‐circuit voltage (*V_OC_*) and the corresponding power‐fill factor (FF) of PSCs [42]. Furthermore, Kelvin probe force microscopy (KPFM) measured the surface contact potential difference (CPD) of bare NiO_x_ and SAM‐modified NiO_x_ (Figure 2c,d). The WFs were calculated as shown in Table S5, corresponding to the UPS measurement. The monophosphate‐anchored SAMs (M3PAICz‐1 and M3PAICz‐2) exhibit narrower CPD distributions than their corresponding bisphosphonate‐anchored SAMs (D3PAICz‐1 and D3PAICz‐2). Notably, although the NiO_x_/D3PAICz‐1 sample exhibits a deep work function, its CPD variation amplitude is significantly larger than that of other samples. This may explain why its hole extraction efficiency is substantially lower than that of M3PAICz‐1.

**FIGURE 3:**
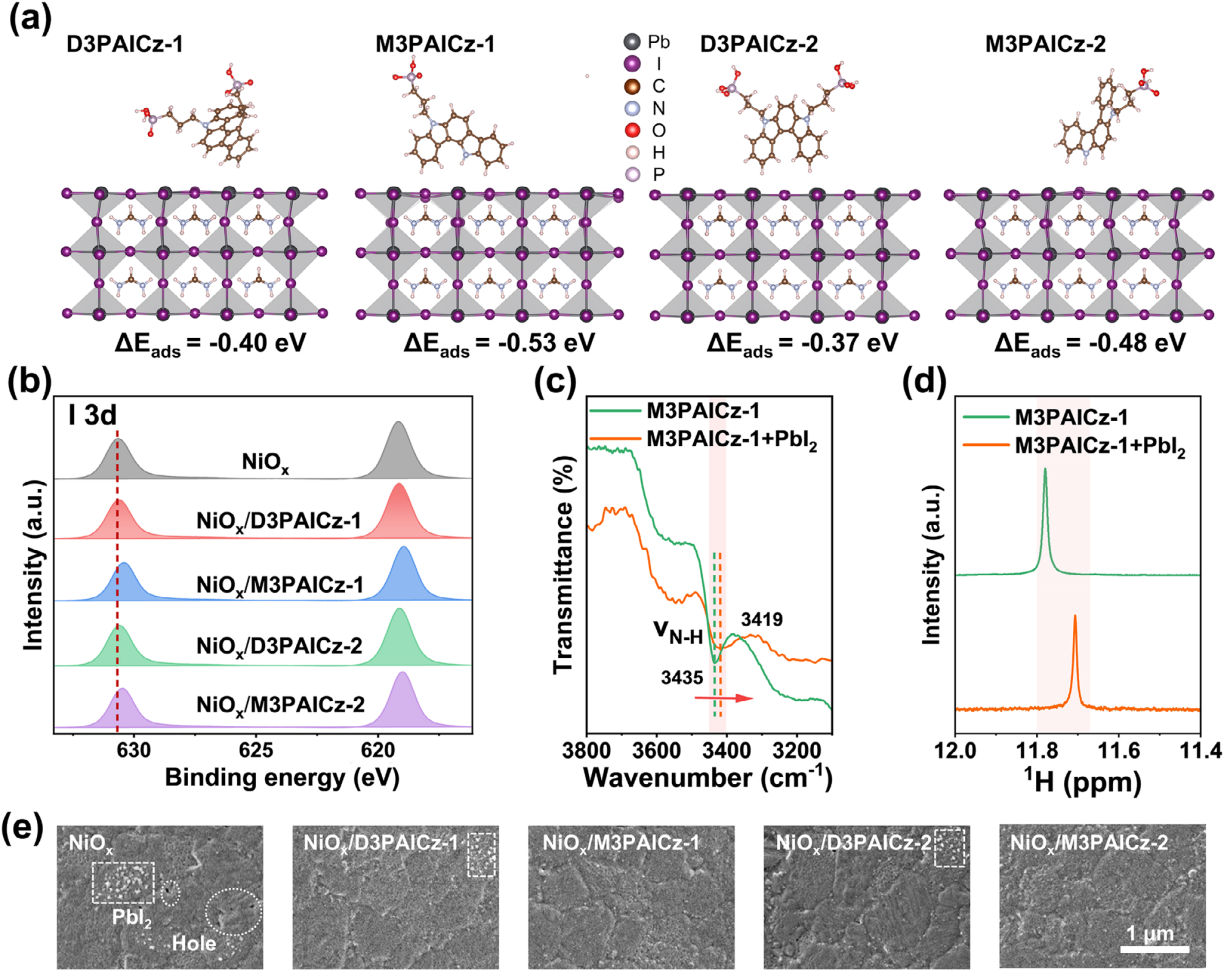
(a) Stick‐and‐ball models and DFT adsorption energies of different SAM molecules on Pb‐terminated perovskite surface. (b) I 3d XPS for the perovskite films deposited on different HTLs. (c) FTIR and (d) ^1^H NMR spectra of M3PAICz‐1 and PbI_2_+M3PAICz‐1 composite materials. (e) Bottom‐view SEM images of perovskite films deposited on different HTLs.

To investigate the SAMs/perovskite interaction, the adsorption energy (*ΔE*_ads_) of SAM molecules on perovskite was determined by DFT calculation, selecting FAPbI_3_ as the perovskite layer for simplicity. As shown in Figure 3a, the *ΔE*_ads_ for D3PAICz‐1, M3PAICz‐1, D3PAICz‐2, and M3PAICz‐2 with FAPbI_3_ were determined to be −0.40, −0.53, −0.37, and −0.48 eV, respectively, indicating a more effective passivation ability of M3PAICz‐1 and M3PAICz‐2, with M3PAICz‐1 demonstrating the strongest passivation ability. Figure S52 illustrates the differential charge density distribution and its longitudinal integral curve of the optimized SAM/perovskite interface. Compared to the biphosphate‐anchored SAMs, both monophosphate SAMs exhibit more peaks at the interface, indicating enhanced charge transfer effects. The red circles indicate charge transfer at the N─H…I hydrogen bond sites, confirming the effectiveness of the hydrogen bonding interaction. Notably, the peak intensity for M3PAICz‐1 is significantly stronger than that for M3PAICz‐2, indicating the larger charge transfer between M3PAICz‐1 and perovskite. The space charge limited current (SCLC) measurements yielded the hole trap density (*n_t_*) for hole‐only devices under dark conditions (Figures S53 and S54) [43]. The *n_t_* value of the perovskite film on bare NiO_x_ was determined to be 4.97×10^15^ cm^−2^, which decreased to 3.96, 2.92, 4.27, and 3.07×10^15^ cm^−2^ for perovskite deposited on NiO_x_/D3PAICz‐1, NiO_x_/M3PAICz‐1, NiO_x_/D3PAICz‐2, and NiO_x_/M3PAICz‐2, respectively. The results showed that the defects were effectively passivated after SAMs modification, particularly in M3PAICz‐1, consistent with DFT calculations.

The interactions of M3PAICz‐1 and M3PAICz‐2 with perovskite were further confirmed by XPS spectra and Fourier transform infrared spectroscopy (FTIR). As shown in Figure 3b, the I 3d peaks of the perovskite films grown on NiO_x_/SAMs shifted to a lower binding energy compared to the control film. This change in binding energy is more pronounced in Perovskite films with M3PAICz‐1 and M3PAICz‐2, and can be attributed to the formation of hydrogen bonds between N─H and I^−^ [44]. The same phenomenon was also observed in Pb 4f XPS spectra in Figure S55, which we attribute to the electron‐donating properties of the ICz unit [45]. Notably, the reduction of metallic Pb in SAMs‐based perovskite films demonstrates the effective passivation of non‐coordinated Pb^2+^ ions [46]. As shown in Figure 3c and Figure S56, after mixing with PbI_2_, the N─H stretching vibration peaks of both M3PAICz‐1 and M3PAICz‐2 became weaker and migrated toward the lower frequency from 3435 and 3397 cm^−^^1^ to 3419 and 3391 cm^−1^, respectively, which indicated that the N─H bond strength is weakened owing to the formation of the hydrogen bond between N─H bond and iodide ions. To further understand atomic‐level chemical interactions between the SAMs and perovskite, we performed ^1^H NMR on the pristine SAMs and their mixtures with PbI_2_, PbBr_2_, and FAI. The ^1^H NMR spectra reveal that after mixing M3PAICz‐1 with PbI_2_, FAI, and PbBr_2_, respectively, the N─H chemical shift shifted upfield from 11.78 ppm to 11.71, 11.70, and 11.73 ppm, respectively, while the chemical shifts of the remaining peaks remained essentially unchanged (Figure 3d; Figure S58). This rules out global electronic or environmental effects caused by halide‐induced shielding. Similarly, after mixing M3PAICz‐2 with PbI_2_, FAI, and PbBr_2_, the N‐H chemical shift shifted from 11.55 ppm to higher fields at 11.47, 11.43, and 11.48 ppm, respectively, while the chemical shifts of the remaining peaks remained essentially unchanged (Figure S59). These shifts could be attributed to the formation of hydrogen bonds, which increase electron density around hydrogen atoms, thereby enhancing the shielding effect. For D3PAICz‐1 and D3PAICz‐2 (Figures S60 and S61), the chemical shifts of all peaks remained essentially unchanged before and after mixing with PbI_2_, PbBr_2_, and FAI, further ruling out global electronic or environmental effects caused by halide‐induced shielding.

To evaluate the quality of perovskite layers grown on NiO_x_ films with and without SAM modification, scanning electron microscopy (SEM) was conducted. As shown in Figure S62a, the average grain size of perovskite deposited on bare NiO_x_ was measured to be 502 nm, significantly smaller than that on NiO_x_ modified with D3PAICz‐1 (618 nm), M3PAICz‐1 (723 nm), D3PAICz‐2 (643 nm), and M3PAICz‐2 (658 nm). In contrast, the SAM‐modified NiOx substrates, particularly those treated with M3PAICz‐1 and M3PAICz‐2, promoted the formation of larger perovskite grains. This increase in grain size led to a reduction in grain boundary density, thereby improving charge carrier transport across the perovskite layer. Atomic force microscopy (AFM) measurements (Figure S62b) indicated a reduction in the RMS roughness of perovskite films deposited on SAM‐modified NiO_x_ substrates, with values of 22.2 nm (NiO_x_/D3PAICz‐1), 17.2 nm (NiO_x_/M3PAICz‐1), 22.3 nm (NiO_x_/D3PAICz‐2), and 17.8 nm (NiO_x_/M3PAICz‐2), compared to 25.3 nm on bare NiO_x_. This decrease in roughness reflects improved surface homogeneity in perovskite films based on M3PAICz‐1 and M3PAICz‐2. Bottom‐view SEM images (Figure 3e) revealed the presence of conspicuous PbI_2_ particles and holes in the perovskite layer without SAM modification, along with a small number of PbI_2_ particles in layers with D3PAICz‐1 and D3PAICz‐2. In contrast, perovskite films modified with M3PAICz‐1 or M3PAICz‐2 exhibited smoother and more uniform buried interfaces, completely free of PbI_2_ particles and holes, indicating enhanced interfacial contact properties. These findings further demonstrate that M3PAICz‐1 and M3PAICz‐2 effectively optimize the morphology of the perovskite film. The X‐ray diffraction (XRD) patterns of perovskite films deposited on the different HTLs are shown in Figure S63, further confirming this finding. The SAM‐modified samples exhibited stronger diffraction intensity at 14.2°, corresponding to the (100) crystal plane, and significantly higher intensity ratios of the (100) peak to other peaks compared to the film on bare NiO_x_. The full width at half maximum (FWHM) of the dominant (100) diffraction peak was measured for all samples. Results showed that the FWHM was significantly narrower for perovskites on SAM‐modified NiO_x_ substrates than on bare NiO_x_, reflecting an increase in grain size and enhanced crystallinity. This improvement was particularly pronounced for the M3PAICz‐1‐ and M3PAICz‐2‐modified samples. The UV–vis absorption spectra of the different perovskite films are presented in Figure S64. In the visible region, the absorption strengths of perovskite films deposited on different HTLs are ranked as follows: NiO_x_/M3PAIC‐1> NiO_x_/M3PAIC‐2>NiO_x_/D3PAIC‐1> NiO_x_/D3PAIC‐2> bare NiO_x_. These findings collectively demonstrate that the introduction of SAMs, particularly M3PAICz‐1 and M3PAICz‐1, can effectively enhance the crystallinity and film quality of perovskite layers.

**FIGURE 4:**
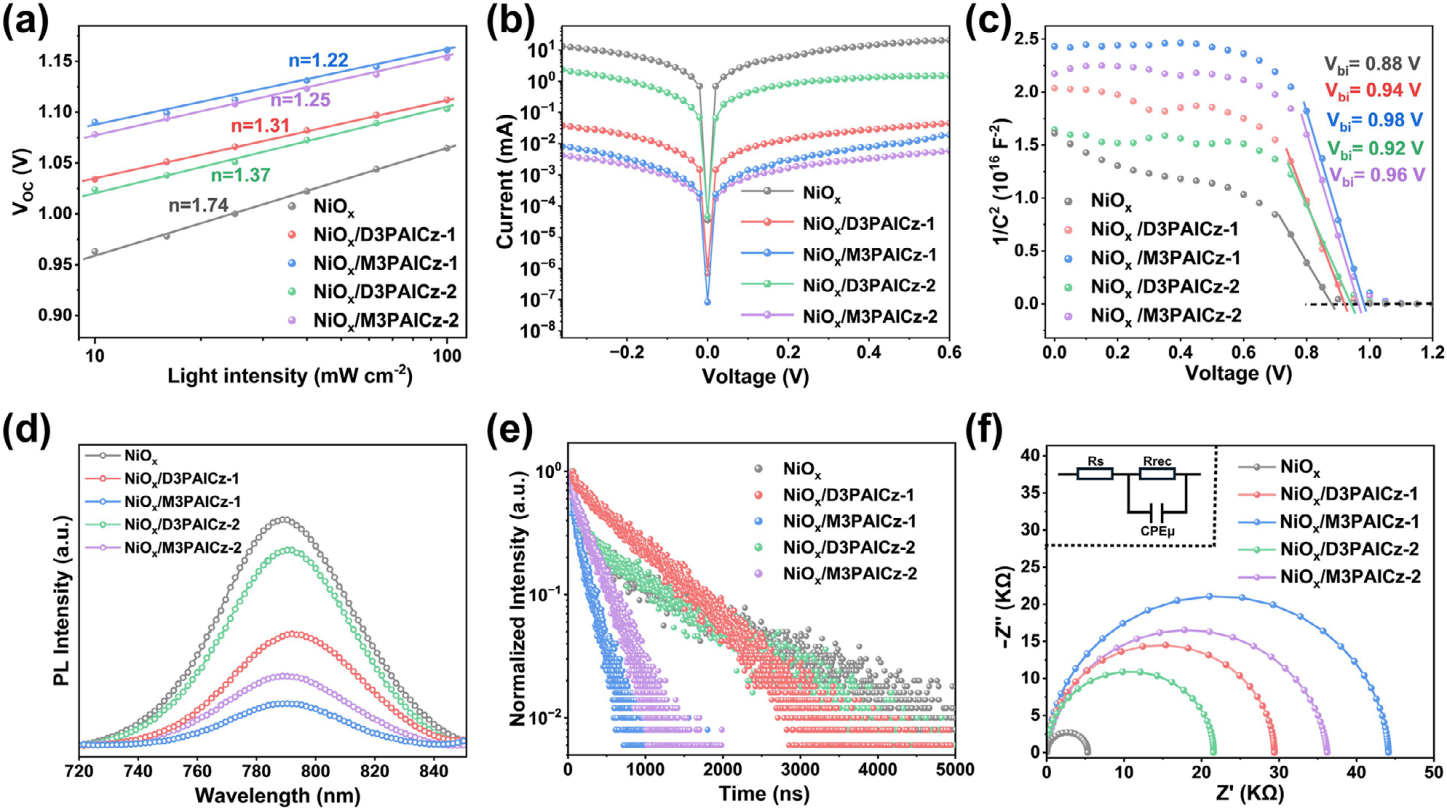
(a) Light intensity‐dependent VOC. (b) Dark *J*–*V* curves. (c) Mott–Schottky plots. (d) PL and (e) TRPL spectra of perovskite films deposited on different HTLs. (f) EIS measurements.

To further investigate the influences of improved perovskite films on the PSC performance, the ideal factor (*n*) of the device was calculated through the *V_OC_*‐light intensity curve in Figure 4a, which is related to the charge extraction process or charge carrier recombination. The *n* values of NiO_x_‐based PSCs modified with different SAMs were determined with a sequence of NiO_x_/M3PAICz‐1 (1.22) < NiO_x_/M3PAICz‐2 (1.25) < NiO_x_/D3PAICz‐1 (1.31) <NiO_x_/D3PAICz‐2 (1.37) < bare NiO_x_ (1.74). For devices based on NiO_x_/M3PAICz‐1 and NiO_x_/M3PAICz‐2, the reduced *n* value benefited from diminished trap‐involved non‐radiative recombination [47]. Moreover, according to the dark Tafel curves for perovskite samples with different HTLs (Figure 4b), the NiO_x_‐based PSCs modified with M3PAICz‐1 and M3PAICz‐2 exhibited lower leakage current owing to improved interfacial contact and reduced recombination losses. Furthermore, Mott‐Schottky measurement was implemented to evaluate the built‐in potential (*V_bi_*). The *V_bi_* value of NiO_x_‐based devices was determined to be 0.88 V, which increased to 0.94, 0.98, 0.92, and 0.96 V for devices based on NiO_x_/D3PAICz‐1, NiO_x_/M3PAICz‐1, NiO_x_/D3PAICz‐2, and NiO_x_/M3PAICz‐2, respectively (Figure 4c). Compared to bare NiO_x_, the carrier transport driving force in devices based on NiO_x_/M3PAICz‐1 and NiO_x_/M3PAICz‐2 was enhanced through fine‐tuned energy level alignment and defect passivation.

Steady‐state and time‐resolved photoluminescence (TRPL) measurements were employed to characterize the charge carrier dynamics across the NiO_x_/SAM/perovskite interface. The SAM‐modified samples showed a larger quenching in PL intensity compared to the reference sample, with the strongest quenching observed for perovskite on NiO_x_/M3PAICz‐1, indicating the most efficient charge transfer. Furthermore, we analyzed the TRPL curves in Figure 4d using a biexponential decay model, with the derived fitting parameters detailed in Table S6. The perovskite film deposited on bare NiO_x_ exhibited an average decay time (τ_ave_) of 1190.4 ns, and when the films were deposited on NiO_x_/D3PAICz‐1, NiO_x_/M3PAICz‐1, NiO_x_/D3PAICz‐2, and NiO_x_/M3PAICz‐2, the decay time dramatically decreased to 722.3, 158.3, 933.2, and 198.1 ns, respectively (Figure 4e). The shortest decay lifetime observed for perovskite films on NiO_x_/M3PAICz‐1 indicates enhanced hole extraction at the interface, thereby suppressing charge recombination [48]. This finding aligns with the steady‐state PL measurements. Additionally, Electrochemical Impedance Spectroscopy (EIS) was employed, and the fitted parameters based on the equivalent circuit model are listed in Table S7. The devices with M3PAICz‐1 exhibited higher charge recombination resistance (*Rrec*) than other devices (Figure 4f), indicating weak trap‐assisted charge recombination and improved interfacial carrier transport.

**FIGURE 5:**
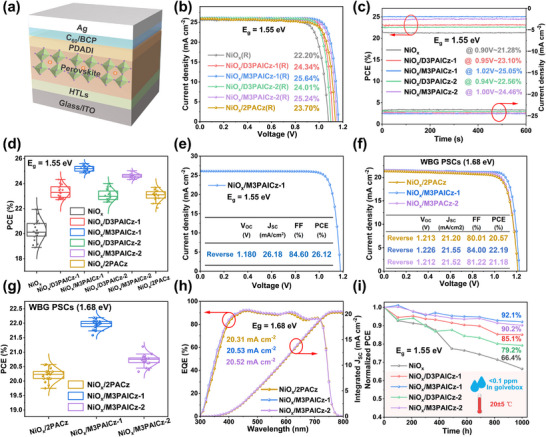
(a) The device structure of the PSCs. (b) *J*–*V* curves and (c) SPOs of the best cells (1.55 eV). (d) The statistics of PCE values of the corresponding 20 devices. (e) *J*–*V* curve of the champion M3PAICz‐1‐based PSC with pre‐synthesized single‐crystals and 2D perovskite. (f) *J*–*V* curves, (g) the statistics of PCE values, and (h) EQEs of the corresponding WBG PSCs. (i) The PCE degradation curves of PSCs with different HTLs at room temperature in a glovebox.

The impact of different HTLs on photovoltaic properties was first evaluated with 1.55 eV bandgap perovskite in device structure as ITO/HTLs/perovskite/1,3‐propanediammonium iodide (PDADI)/C60/bathocuproine (BCP)/Ag, with device fabrication detailed in Supporting Information. As shown in Figure S65, the optimized concentration of all SAMs was found to be 0.5 mg/mL in ethanol. The current density–voltage (*J*–*V*) curves of the champion devices based on different HTLs under AM 1.5G‐1‐sun illumination are shown in Figure 5b and Figure S66, and the photovoltaic parameters were detailed in Table S8. The bare NiO_x_‐based device achieved a PCE of 22.20% with severe hysteresis. With SAM modification, all devices showed significantly enhanced performance. The NiO_x_/2PACz‐based reference PSCs exhibited a PCE of 23.70% with apparent hysteresis. In contrast, the device with NiO_x_/M3PAICz‐1 exhibited the best performance with a PCE of 25.64% in the reverse scan, which is higher than those of the devices involving D3PAICz‐1, D3PAICz‐2, and M3PAICz‐2 (PCEs of 24.34%, 24.01%, and 25.24%, respectively). A stabilized PCE of 25.05% from steady‐state power output (SPO) confirmed the reliability of the device (Figure 5c). The NiO_x_/M3PAICz‐1‐based device also showed a narrower distribution of the photovoltaic parameters (Figure 5d; Figure S67). Moreover, the M3PAICz‐1‐involved device, fabricated with pre‐synthesized single‐crystals as solutes and with a passivated interface to the corresponding 2D perovskite (Note S2, Figures S68 and S69) exhibited the champion PCE of 26.12% with a *V_OC_* of 1.180 V, a *J_SC_* of 26.18 mA/cm^2^, and an FF of 84.60% (Figure 5e; Figure S70). Given the significantly superior performance of M3PAICz‐1 and M3PAICz‐2 over other SAMs, we applied them to 1.68 eV WBG PSCs (Figure S72), both of which achieved higher PCE than NiO_x_/2PACz‐based devices (Figure 5g). Encouragingly, the NiO_x_/M3PAICz‐1‐based WBG devices achieved an impressive PCE of 22.19% in reverse scan (Figure 5f), demonstrating the generality of M3PAICz‐1 as HTL. In addition, the integrated *J_SC_* of the PSCs, calculated from the external quantum efficiency (EQE) curves (Figure 5h; Figure S73), closely matches the *J‐V* results, with an error within 5%, indicating all the values are reasonable.

The device stability was evaluated by exposing unencapsulated 1.55 eV PSCs to various storage conditions. After 1000 h at 20 ± 5°C in the dark (Ar glovebox), devices based on NiO_x_/D3PAICz‐1, NiO_x_/M3PAICz‐1, NiO_x_/D3PAICz‐2, and NiO_x_/M3PAICz‐2 retained their initial PCE of 85.1%, 92.1%, 79.2% and 90.2%, respectively, versus 66.4% for bare NiO_x_ (Figure 5i). Upon aging at 20 ± 5°C with 35 ± 5% relative humidity (RH) under dark conditions in ambient air, devices based on NiO_x_/D3PAICz‐1, NiO_x_/M3PAICz‐1, NiO_x_/D3PAICz‐2, and NiO_x_/M3PAICz‐2 maintained 72.7%, 87.3%, 71.5%, and 82.9% of their initial PCEs after 1000 h, while the bare NiO_x_‐based device only retained 46.6% initial PCE (Figure S74). The results demonstrate that ICz‐based monophosphonate‐anchored SAMs exhibited enhanced stability compared to their bisphosphonate‐anchored counterparts. Notably, devices modified with M3PAICz‐1 showed superior stability, attributed to the stronger interaction with perovskite and improved crystal quality.

## 3 Conclusion

In summary, we have systematically developed a novel series of indolocarbazole‐based SAMs by modulating the orientation and number of the phosphonic acid anchoring groups. It was found that both the position and number of anchoring groups influence the SAM in three main aspects: the interaction with the NiOx substrate, the intermolecular interactions among the SAM molecules, and the interaction between the SAM and the perovskite layer. Among these molecules, the monophosphonate‐anchored meta‐M3PAICz‐1 exhibits the largest dipole moment and the deepest HOMO level, resulting in an enlarged work function for the NiO_x_/M3PAICz‐1 substrate and consequently improved hole extraction in the device. Meanwhile, M3PAICz‐1 forms the densest SAM layer on the NiO_x_ surface, minimizing direct contact between the perovskite and NiO_x_. More importantly, the N─H group of M3PAICz‐1 can form stronger interactions with the perovskite through hydrogen bonding, leading to the most effective passivation of perovskite defects. Consequently, the 1.55 eV PSC with M3PAICz‐1 exhibited a champion PCE of 26.12%. Notably, the WBG PSC (1.68 eV) also achieved an outstanding PCE of 22.19%, demonstrating the broad applicability of M3PAICz‐1 as HTL. Moreover, due to improved perovskite crystallinity, PSCs with M3PAICz‐1 exhibited notably enhanced stability, maintaining 87.3% of the original PCE after 1000 h of storage in ambient air (20 ± 5°C, 35 ± 5% RH) without encapsulation. Overall, our strategy for modulating the orientation and number of phosphonic acid anchoring groups in indolocarbazole‐based SAMs provides valuable insights for designing SAMs to achieve efficient and stable PSCs.

## 4 Experimental Section

Detailed experimental procedures can be found in the Supporting Information.

## Conflicts of Interest

The authors declare no conflicts of interest.

## Supporting information




**Supporting file**: smll72463‐sup‐0001‐SuppMat.docx

**Supporting file**: smll72463‐sup‐0002‐SuppMat.docx

## Data Availability

The data that support the findings of this study are available in the supplementary material of this article.

## References

[1] A. Fan , M. An , T. K. Zhang , et al., “Corannulene‐Derivative‐Interfaced Inverted Perovskite Solar Cells With a Fill Factor Above 0.87,” Advanced Functional Materials 35 (2025): 10193, 10.1002/adfm.202510193.

[2] Z. Cui , W. Li , B. Feng , et al., “Thickness‐Insensitive Polymeric Hole‐Transporting Layer For Efficient Inverted Perovskite Solar Cells,” Joule 9 (2025): 102011, 10.1016/j.joule.2025.102011.

[3] L. Yuan , Q. Xue , F. Wang , et al., “Perovskite Solar Cells and Light Emitting Diodes: Materials Chemistry, Device Physics and Relationship,” Chemical Reviews 125 (2025): 5057–5162, 10.1021/acs.chemrev.4c00663. 40397873

[4] Y. Lin , Z. Lin , S. Lv , et al., “A Nd@C_82_–Polymer Interface For Efficient And Stable Perovskite Solar Cells,” Nature 642 (2025): 78–84, 10.1038/s41586-025-08961-9. 40199342

[5] P. Chen , Y. Xiao , S. Li , et al., “The Promise and Challenges of Inverted Perovskite Solar Cells,” Chemical Reviews 124 (2024): 10623–10700, 10.1021/acs.chemrev.4c00073. 39207782

[6] J. Zhang , W. Zhang , H.‐M. Cheng , and S. R. P. Silva , “Critical Review Of Recent Progress Of Flexible Perovskite Solar Cells,” Materials Today 39 (2020): 66–88, 10.1016/j.mattod.2020.05.002.

[7] Q. Jiang , J. Tong , Y. Xian , et al., “Surface Reaction For Efficient And Stable Inverted Perovskite Solar Cells,” Nature 611 (2022): 278–283, 10.1038/s41586-022-05268-x. 36049505

[8] M. A. Green , E. D. Dunlop , M. Yoshita , et al., “Solar Cell Efficiency Tables (Version 66),” Progress in Photovoltaics: Research and Applications 33 (2025): 795–810, 10.1002/pip.3919.

[9] L. Zhan , S. Zhang , Z. Li , et al., “Anchorable Polymers Enabling Ultra‐Thin and Robust Hole‐Transporting Layers for High‐Efficiency Inverted Perovskite Solar Cells,” Angewandte Chemie International Edition 64 (2025): 202422571, 10.1002/anie.202422571. 39780690

[10] M. W. Dong , Y. Li , Y. Yang , et al., “Self‐Assembled Bilayer For Perovskite Solar Cells With Improved Tolerance Against Thermal Stresses,” Nature Energy 10 (2025): 342–353.

[11] S. Qu , F. Yang , H. Huang , et al., “Redox Mediator‐Modified Self‐Assembled Monolayer Stabilizes A Buried Interface In Efficient Inverted Perovskite Solar Cells,” Energy & Environmental Science 18 (2025): 3186–3195, 10.1039/D4EE05319B.

[12] E. Aydin , E. Ugur , B. K. Yildirim , et al., “Enhanced Optoelectronic Coupling For Perovskite/Silicon Tandem Solar Cells,” Nature 623 (2023): 732–738, 10.1038/s41586-023-06667-4. 37769785

[13] S. Wu , Y. Yan , J. Yin , et al., “Redox Mediator‐Stabilized Wide‐Bandgap Perovskites For Monolithic Perovskite‐Organic Tandem Solar Cells,” Nature Energy 9 (2024): 411–421, 10.1038/s41560-024-01451-8.

[14] Y. C. Li , Z. Zhang , C. Liu , F. Guo , W. Ahmad , and P. Gao , “Pros and Cons Of Hole‐Selective Self‐Assembled Monolayers In Inverted PSCs and TSCs: Extensive Case Studies And Data Analysis,” Energy & Environmental Science 17 (2024): 6157–6203, 10.1039/D4EE02492C.

[15] Y. Xu , C. Wang , U. Amornkitbamrung , et al., “Molecular Bridge on Buried Interface for Energy Level Alignment in Inverted Perovskite Solar Cell With Efficiency Over 25%,” ACS Energy Letters 10 (2025): 3407–3414, 10.1021/acsenergylett.5c01437.

[16] X. Wu , H. Yang , Z. Yue , et al., “Hydroxyl‐Driven Homogeneous And Robust SAM Anchoring on NiO_x_ for High‐Performance Inverted Perovskite Solar Cells,” Chemical Engineering Journal 520 (2025): 166008, 10.1016/j.cej.2025.166008.

[17] H. Bi , J. Liu , L. Wang , et al., “Selective Contact Self‐Assembled Molecules For High‐Performance Perovskite Solar Cells,” eScience 5 (2025): 100329, 10.1016/j.esci.2024.100329.

[18] J. Wang , B. Jiao , R. Tian , et al., “Less‐Acidic Boric Acid‐Functionalized Self‐Assembled Monolayer For Mitigating NiO_x_ Corrosion For Efficient All‐Perovskite Tandem Solar Cells,” Nature Communications 16 (2025): 4148, 10.1038/s41467-025-59515-6. PMC12049437 40319016

[19] X. Huang , T. Wang , W. Chen , et al., “Bottom‐Up Regulation of Perovskite Growth and Energetics via Oligoether Functionalized Self‐Assembling Molecules for High‐Performance Solar Cells,” Angewandte Chemie International Edition 64 (2025): 202507513, 10.1002/anie.202507513. 40598821

[20] Q. Cao , T. Wang , X. Pu , et al., “Co‐Self‐Assembled Monolayers Modified NiO_x_ for Stable Inverted Perovskite Solar Cells,” Advanced Materials 36 (2024): 2311970, 10.1002/adma.202311970. 38198824

[21] X. Zhang , Z. Cao , L. Shen , et al., “Unraveling the Combined Photothermal Stability of Common Perovskite Solar Cell Compositions,” ACS Energy Letters 9 (2024): 5728–5736, 10.1021/acsenergylett.4c02325.

[22] M. Liu , L. Bi , W. Jiang , et al., “Compact Hole‐Selective Self‐Assembled Monolayers Enabled by Disassembling Micelles in Solution for Efficient Perovskite Solar Cells,” Advanced Materials 35 (2023): 2304415, 10.1002/adma.202304415. 37487572

[23] S. Lenaers , S. Lammar , A. Krishna , et al., “Pyrene‐Based Self‐Assembled Monolayer with Improved Surface Coverage and Energy Level Alignment for Perovskite Solar Cells,” Advanced Functional Materials (2024): 202411922.

[24] J. Roe , J. G. Son , S. Park , et al., “Synergistic Buried Interface Regulation of Tin–Lead Perovskite Solar Cells via Co‐Self‐Assembled Monolayers,” ACS Nano 18 (2024): 24306–24316, 10.1021/acsnano.4c06396. 39172688

[25] P. Han and Y. Zhang , “Recent Advances in Carbazole‐Based Self‐Assembled Monolayer for Solution‐Processed Optoelectronic Devices,” Advanced Materials 36 (2024): 2405630, 10.1002/adma.202405630. 38940073

[26] X. Zhu , C. F. J. Lau , K. Mo , et al., “Inverted Planar Heterojunction Perovskite Solar Cells With High Ultraviolet Stability,” Nano Energy 103 (2022): 107849, 10.1016/j.nanoen.2022.107849.

[27] J. Du , J. Chen , B. Ouyang , et al., “Face‐on Oriented Self‐Assembled Molecules With Enhanced π–π Stacking For Highly Efficient Inverted Perovskite Solar Cells On Rough FTO Substrates,” Energy & Environmental Science 18 (2025): 3196–3210, 10.1039/D4EE05849F.

[28] S. Hu , S. Zeng , X. Deng , et al., “Scalable Impregnation Method for Preparing a Self‐Assembled Monolayer in High‐Performance Vapor‐Deposited Lead‐Halide Perovskite Solar Cells,” ACS Nano 19 (2025): 15018–15029. 40193592 10.1021/acsnano.5c01479

[29] D. Song , S. W. Shin , H.‐P. Wu , E. W.‐G. Diau , and J.‐P. Correa‐Baena , “Toward Maximizing Hole Selection With Self‐Assembled Monolayers in Sn‐Based Perovskite Solar Cells,” ACS Energy Letters 10 (2025): 1292–1312, 10.1021/acsenergylett.4c03228.

[30] R. Xu , C. Wang , Z. Zhang , et al., “Buried Interface Engineering: a Key to Unlocking the Potential of Self‐Assembled Monolayer (SAM)‐Based Inverted Perovskite Solar Cells,” Small 21 (2025): 2503114, 10.1002/smll.202503114. 40538302

[31] J. M. Ramón , J. G. Sánchez , M. Más‐Montoya , et al., “Revealing the Role of Spacer Length and Methoxy Substitution of Dipodal Indolocarbazole‐based SAMs on the Performance of Inverted Perovskite Solar Cells,” Small 21 (2025): 2500067. 40270211 10.1002/smll.202500067 PMC12138858

[32] H. Liu , X. Gao , Y. Xin , et al., “Central Fluorination Strategy Of Biphosphonic Acid Molecule For Self‐Assembled Monolayer Enables Efficient Organic Solar Cells,” Science China Materials 68 (2025): 1408–1414.

[33] H. Liu , Y. Xin , Z. Suo , et al., “Dipole Moments Regulation of Biphosphonic Acid Molecules for Self‐assembled Monolayers Boosts the Efficiency of Organic Solar Cells Exceeding 19.7%,” Journal of the American Chemical Society 146 (2024): 14287–14296, 10.1021/jacs.4c03917. 38718348

[34] W. Jiang , F. Li , M. Li , F. Qi , F. R. Lin , and A. K. Y. Jen , “π‐Expanded Carbazoles as Hole‐Selective Self‐Assembled Monolayers for High‐Performance Perovskite Solar Cells,” Angewandte Chemie International Edition 61 (2022): 202213560, 10.1002/anie.202213560. 36300589

[35] H. Chen , G. W. Liu , X. Chen , et al., “Methylthio Substituent in SAM Constructing Regulatory Bridge With Photovoltaic Perovskites,” Angewandte Chemie International Edition 64 (2024): 202419375, 10.1002/anie.202419375. 39618342

[36] J. Wu , P. Yan , D. Yang , et al., “Bisphosphonate‐Anchored Self‐Assembled Molecules With Larger Dipole Moments for Efficient Inverted Perovskite Solar Cells With Excellent Stability,” Advanced Materials 36 (2024): 2401537, 10.1002/adma.202401537. 38768481

[37] Z.‐R. Lan , D.‐X. Ma , K. Tang , J. Yao , J.‐Y. Shao , and Y.‐W. Zhong , “Indolo[3,2‐b]carbazole‐Based Self‐Assembled Monolayer Enables High‐Performance Inverted Perovskite Solar Cells With Over 25.5% Efficiency,” CCS Chemistry 7 (2025): 2672–2680, 10.31635/ccschem.024.202404647.

[38] S. Tu , W. Chen , Y. Gang , Q. Xiong , and X. Li , “Engineering a thermally robust hole‐selective layer for stable flexible perovskite solar cells,” Chemical Engineering Journal 503 (2025): 158389, 10.1016/j.cej.2024.158389.

[39] J. Chen , X. Fan , J. Wang , et al., “23.81%‐Efficiency Flexible Inverted Perovskite Solar Cells With Enhanced Stability And Flexibility Via A Lewis Base Passivation,” ACS Nano 18 (2024): 19190–19199. 38989607 10.1021/acsnano.4c04768

[40] Y. Zhou , X. Huang , J. Zhang , et al., “Interfacial Modification of NiO_x_ for Highly Efficient and Stable Inverted Perovskite Solar Cells,” Advanced Energy Materials 14 (2024): 2400616, 10.1002/aenm.202400616.

[41] J. Zhang , J. Yang , R. Dai , et al., “Elimination of Interfacial Lattice Mismatch and Detrimental Reaction by Self‐Assembled Layer Dual‐Passivation for Efficient and Stable Inverted Perovskite Solar Cells,” Advanced Energy Materials 12 (2022): 2103674, 10.1002/aenm.202103674.

[42] P. Zhao , D. He , X. Fu , et al., “Thiophene Substituent Engineering of Carbazole Based Self‐Assembled Monolayers for Use in High‐Performance Inverted Perovskite Solar Cells,” Small 21 (2025): 2500281, 10.1002/smll.202500281. 39981964

[43] M. Liu , M. Li , Y. Li , et al., “Defect‐Passivating and Stable Benzothiophene‐Based Self‐Assembled Monolayer for High‐Performance Inverted Perovskite Solar Cells,” Advanced Energy Materials 14 (2024): 2303742, 10.1002/aenm.202303742.

[44] T. Li , C. Wang , C. Hu , et al., “Auxiliary Buried‐Interface Passivation Toward Stable and Low‐Recombination‐Loss Perovskite Photovoltaics,” Small Science 4 (2023): 2300218. 40212618 10.1002/smsc.202300218 PMC11935047

[45] C. Li , N. Zhang , and P. Gao , “Lessons Learned: How to Report XPS Data Incorrectly About Lead‐Halide Perovskites,” Materials Chemistry Frontiers 7 (2023): 3797–3802, 10.1039/D3QM00574G.

[46] L. Chi , Q. Junming , Z. Mengqi , et al., “Multifunctional Anionic Metal‐Organic Frameworks Enhancing Stability Of Perovskite Solar Cells,” Chemical Engineering Journal 433 (2022): 133587.

[47] M. Wolff , P. Caprioglio , M. Stolterfoht , and D. Neher , “Nonradiative Recombination in Perovskite Solar Cells: The Role of Interfaces,” Advanced Materials 31 (2019): 1902762, 10.1002/adma.201902762. 31631441

[48] Q. Tan , Z. Li , G. Luo , et al., “Inverted Perovskite Solar Cells Using Dimethylacridine‐Based Dopants,” Nature 620 (2023): 545–551, 10.1038/s41586-023-06207-0. 37224876

