# Supplementary material for: Defect‐Passivating and Dense Indolocarbazole‐Based Self‐Assembled Monolayers for Efficient Inverted Perovskite Solar Cells With over 26.1% Efficiency
Source: Small. 2026 Jan 19;22(16):e12942. doi: 10.1002/smll.202512942 (PMC12994557; doi:10.1002/smll.202512942)
Supplement: Supplementary file 1 — Supporting file: smll72463‐sup‐0001‐SuppMat.docx [file SMLL-22-e12942-s001.docx]

**Supporting Information**

# Defect-Passivating and dense Indolocarbazole-Based Self-Assembled Monolayers for Efficient Inverted Perovskite Solar Cells with Over 26.1% Efficiency

Xu Fu,^1, 3^ Yuxuan Yang,^1, 4^ Dingqian He,^1,3^ Peng Zhao,^1,3^ Huixin Gao,^1,3^ Zhen Zhu,^2^ Yi Zhang,^1, 4,*^ Bao Zhang^1,3,*^, and Mohammad Khaja Nazeeruddin^2,*^

^1^School of Chemical Engineering and Technology, Tianjin University, Tianjin 300350, P.R. China

^2^School of Integrated Circuits, Southeast University, Wuxi, 214026 Jiangsu, P. R. China

^3^Collaborative Innovation Center of Chemical Science and Engineering, Tianjin University, Tianjin 300072, P.R. China

^4^Institute of Molecular Plus, School of Chemical Engineering and Technology, Tianjin University, Tianjin 300072, P.R. China

E-mail: yi_zhang@tju.edu.cn, baozhang@tju.edu.cn, [mdkhaja.nazeeruddin@epfl.ch](mailto:mdkhaja.nazeeruddin@epfl.ch)

1. **Materials**

All starting chemicals, reagents, and solvents used for the material synthesis were purchased from commercial suppliers with regent grade and used without further purification unless otherwise noted.

For the materials used for device fabrication, indium tin oxide glass (ITO), NiO_x_, PbI_2_ (99.999%), [2-(9H-carbazol-9-yl)ethyl]phosphonic acid (2PACz, 98%), propylenediamine dihydroiodine (PDADI) and C60 were purchased from Liaoning Youxuan Technology Corp, China and used directly. BCP and PbBr_2_ (99.99%) were purchased from Xi’an Polymer Light Technology Corp, China and used directly. FAI, MABr and MACl were purchased from Greatcell Solar and used directly. CsI was purchased from Sigma-Aldrich, United States and used directly. All the dissolving and processing solvents including N, N-dimethylformamide (DMF, 99.8%), dimethyl sulfoxide (DMSO, 99.7%), chlorobenzene (CB, 99.8%) and ethanol (99.5%) are anhydrous and are purchased from Sigma-Aldrich, United States. Silver (99.999%) was purchased from Alfa Aesar Chemical Co., Ltd. Single crystals CsPbI_3_ (99.5%) and FAPbI3 (99.5%) were purchased from Zhoushan Huazhou Chemical Co., Ltd. and used directly.

1. **Device fabrication**

ITO glass substrates were ultrasonically cleaned with deionized water, ethanol, acetone and isopropanol for 20 min, respectively, and then dried with N_2_, followed by oxygen plasma cleaning for 30 min before using. The NiO_x_ hole transport layer was formed by spin-coating NiO_x_ nanoparticle ink with a concentration of 10 mg ml^−1^ in a mixed solution of deionized water and IPA (3:1, vol/vol) onto the ITO substrate at 3,000 rpm for 30 s, then annealed at 130 °C for 10 min in an air environment and then transferred to an N2-filled glovebox. The four types of SAMs were dissolved in ethanol with a concentration of 0.5 mg mL^-1^. Then, it was spin-coated on the ITO/NiO_x_ substrates at a speed of 3000 rpm for 30 s and then thermally annealed at 100 ℃ for 10 min. The CsFAMAPI_X_Br_3-X_ perovskite layer was fabricated by the anti-solvent method. Perovskite precursor solutions were prepared in a glove box. For 1.55 eV Cs_0.05_(FA_0.95_MA_0.05_)_0.95_Pb(I_0.95_Br_0.05_)_3_ perovskite, 1.6 M undoped perovskite precursor solution was prepared by dissolving PbI_2_, FAI, PbBr_2_, MABr, CsI, MACl (15 mol%) in 1 mL DMF and DMSO (4:1, v:v). For 1.68 eV Cs_0.05_FA_0.73_MA_0.22_Pb(I0.77Br_0.23_)_3_ wide-bandgap perovskite, 1.6 M perovskite precursor solution was prepared by mixing PbI_2_, FAI, PbBr_2_, MABr, CsI, MACl (15 mol%) in 1 mL DMF and DMSO (4:1, v: v). The perovskite layer was deposited with two-step spin-coating procedures: (1) 1000 rpm for 10 s; (2) 4500 rpm for 32 s, where 170 mL of chlorobenzene was dropped on the spinning substrate during the second spin-coating step at 7 s before the end of the procedure. The prepared perovskite film was annealed at 100 °C for 30 min. The 1,3-propanediammonium iodide (PDADI) solution (5 mg/mL PDADI in IPA/CB (V: V = 2:1)) was deposited on top of the perovskite layer at a rotational speed of 4500 rpm for 25 s, followed by annealing at 100 ℃ for 5 min to form the PDADI passivation layer. We used thermally evaporated doub, including C_60_ (20 nm) and 2,9-Dimethyl-4,7-diphenyl-1,10-phenanthroline (BCP) (7nm), as cathode buffer layer in the device by vacuum evaporation under 2 × 10^-4^ mbar. Finally, an Ag electrode (120 nm) was thermally evaporated (under a vacuum condition of <10^-5^ Pa) using a shadow mask to complete the fabrication. When measuring, a 0.057 cm^2^ mask was used to define the accurate active cell area. The fabrication of 1.68 eV wide bandgap devices is carried out using a similar process as described above, except that the perovskite absorber layer is replaced with Cs_0.05_FA_0.73_MA_0.22_Pb(I_0.77_Br_0.23_)_3_. For high-efficiency 1.55 eV PSCs, in order to enhance phase purity of cubic α-FAPbI3 and reduce defects, the conventional precursor mixture (FAI, PbI_2_, MABr and PbBr_2_) was replaced with presynthesized single crystals. Specifically, a perovskite solution was prepared by dissolving 0.08 mol/L CsPbI3, 0.076 mol/L MAPbBr3, and 1.444 mol/L FAPbI3, along with additional 4.4% PbI_2_ and 25% MACl, in a mixed anhydrous solvent (DMF:DMSO = 4:1). Except for an additional step of passivating defects with two-dimensional perovskite, the other fabrication processes remain unchanged. After perovskite deposition, The PDADI solution (0.5 mg/mL in IPA) was then spin-coated onto the perovskite layer at 4000 rpm for 30 seconds and annealed at 100 °C for 5 minutes. This process resulted in the formation of two-dimensional perovskite at the interface on the perovskite layer.

The hole-only samples for the SCLC measurements were fabricated with a configuration of ITO/NiO_x_/SAMs/perovskite/spiro-OMeTAD/Ag according to the PSC fabrication procedure. The concentration of spiro-OMeTAD solution was 50mg/mL in chlorobenezene, where 30 μL of 4-tert-butylpyridine and 35 μL of lithium bis(trifluoromethane-sulfonyl)imide (Li-TFSI) solution (260 mg Li-TFSI in 1 mL acetonitrile) were added as additives. Spiro-OMeTAD solution was spin-coated onto the perovskite layer at 4000 rpm for 30 s. Finally, 90 nm of silver as the metal electrode on the HTM-coated films was evaporated in a metallization chamber.

1. **Characterization**

The ^1^H and ^13^C NMR spectra of different SAMs were recorded by a AVANCE III HD 400 MHz. The thermogravimetric measurements were performed on Netzsch STA 449 F3 instrument at a heating rate of ℃ min^-1^ in the range of 25 °C to 800 °C under a nitrogen atmosphere. The water contact angle images were taken on a DSA100 contact angle analysis instrument. The ultraviolet photoelectron spectrometer (UPS) measurements were carried out on AXIS Ultra DLD instrument under excitation from the He line (21.2 eV) of a helium discharge lamp. The X-ray photoelectron spectroscopy (XPS) measurements were obtained on an Thermo K-Alpha+ X-ray photoelectron spectrometer and calibrated by the C 1s peak (284.8 eV). Kelvin probe force microscopy (KPFM) tests were observed by an Oxford asylum research cypher atomic force microscopy (AFM) and the work function (WF) of tip is calibrated to be 4.75 eV by the highly oriented pyrolytic graphite (HOPG) with fresh cleavage whose WF is 4.6 eV. AFM images of perovskite surface were acquired using a Bruker Dimension icon AFM. X-ray diffraction (XRD) patterns were obtained from a Bruker D8 Focus X-ray diffractometer with Cu Kα radiation as the X-ray source. UV-vis absorption spectra were obtained on a Shimdazu UV-1800 spectrophotometer. Steady state photoluminescence (PL) was acquired by a Edinburgh FLS1000 fluorescence spectrophotometer, where photoluminescence was excited with a 450 nm Xe lamp. Time-resolved photoluminescence spectra (TRPL) were recorded by Light Conversion Harpia with an excitation of 475 nm pulsed laser (F-7100, HITACHI). The PL experiment’s samples were fabricated with a configuration of ITO/NiO_x_/SAMs/perovskite. The surface and cross-sectional morphology of perovskite were characterized by a field emission scanning electron microscope (SEM, Regulus 8100, Hitachi).

Cyclic voltammetry (CV) measurements were performed at room temperature on a CHI660D electrochemical workstation with a conventional three-electrode system using a glassy carbon electrode as the working electrode, platinum wire as the counter electrode, and an Ag/AgCl (saturated KCl) as the reference electrode. SAMs (5×10^-4^ M) were dissolved in DMF with tetrabutylammonium perchlorate (NBu_4_ClO_4_, 0.1M) as a supporting electrolyte, and the scan rate was 0.05 V/s. For calibration, ferrocene was useed as an external reference and the redox potential of ferrocene/ferrocenium (Fc/Fc^+^) was measured under the same condition.The HOMO energy level was calculated according to the following equation: $\text{E}_{\text{HOMO}}\text{= -[}\text{E}_{\text{ox}}^{\text{oneset}}$+4.8], Where is the onset of oxidation potential vs. Fc/Fc^+^.

The electrochemical impedance spectroscopy (EIS) measurements were conducted with a Chenhua CHI660D electrochemical workstation with a frequency range from 0.001 Hz to 1MHz under an applied voltage of 0.4 V in dark condition. In regard of the Mott-Schottky measurements, the capacitance dependent voltage was measured at a fixed frequency (10 kHz) through the Chenhua CHI660D instrument in the dark with range from 0 V to 1.2 V in positive direction. For EIS and Mott-Schottky measurements, samples were fabricated with a configuration of ITO/NiO_x/_SAMs/perovskite/C60/BCP/Ag.

Regarding the device characterization, the simulated solar illumination (AM 1.5 G, 100 mW cm^−2^) was provided by a solar simulator (SS150, Zolix, CN) and calibrated with a calibrated Si reference cell. The current density-voltage (*J-V*) curves of the PSCs were measured using a Keithley 2400 source meter at a scan speed of 100 mV s^-1^ in an air atmosphere with 30-40% humidity. External quantum efficiency (EQE) was obtained on a computer-controlled quantum efficiency instrument (QE-R, Enli Technology, Inc.). Space-charge-limited current (SCLC) measurements of the hole-only devices were achieved through a Keithley 2400 with a scan step 0.01 V under dark conditions. The SCLC’s samples were fabricated with a configuration of ITO/NiO_x_/SAMs/perovskite/spiro-OMeTAD/Ag.

The conductivity of NiO_x_ films is calculated using the following equation:$\text{ }\text{ }\text{σ}\text{ = }{\frac{\text{d}}{\text{AR}}}$

where σ is the conductivity, d is the thickness of the NiOx film, A is the active area, and R represents the resistance determined from the I–V curves of the NiO_x_ or NiOx/SAM films. The thickness of the NiOx film was determined to be 271 nm based on the cross-sectional SEM image shown in **Figure S54**.

1. **Computational Method**

Calculations for the SAMs molecules were performed using the Gaussian 09W program at the B3LYP/6-311G(d,p) level of theory in the solid state. The electrostatic potential map was computed with the Multiwfn code, which was based on the highly effective algorithm.^[1-3]^Theoretical adsorption mode between SAMs and perovskite was are performed in the framework of the density functional theory (DFT) with the projector augmented plane-wave method, as implemented in the Vienna ab initio simulation package(VASP).^[4]^ The generalized gradient approximation proposed by Perdew, Burke, and Ernzerhof is selected for the exchange-correlation potential.^[5]^ The long-range van der Waals interaction is described by the DFT-D3 approach.^[6]^ The plane wave cut-off energy of 400 eV is adopted, the energy convergence accuracy is set to 1 × 10^−5^ eV/atom, and the force acting on each atom is not greater than 0.01 eV/Å. The Brillouin zone is integrated using a 1 × 1 × 1 k-point grid. The ΔEads was determined by the equation: Δ*E*_ads_ = (*E*_A_−*E*_0_−n*E*s)/n, where the energy of *E*_A_ is the total energy of the optimized perovskite surface adsorbed with the SAMs, the E0 is the total energy of the optimized perovskite surface, and the Es is the energy of optimized SAMs, n is the number of the SAMs.

1. **Synthesis of SAMs and MAPbBr_3_ single crystals**

The synthetic routes to the four SAMs are illustrated in **Scheme S1**. 1,3-Cyclohexanedione, 1,4-Cyclohexanedione, phenylhydrazine hydrochloride, and diethyl 4-bromobutylphosphonate were purchased from BIDE Pharm. All the chemicals were used as-received without further purification.


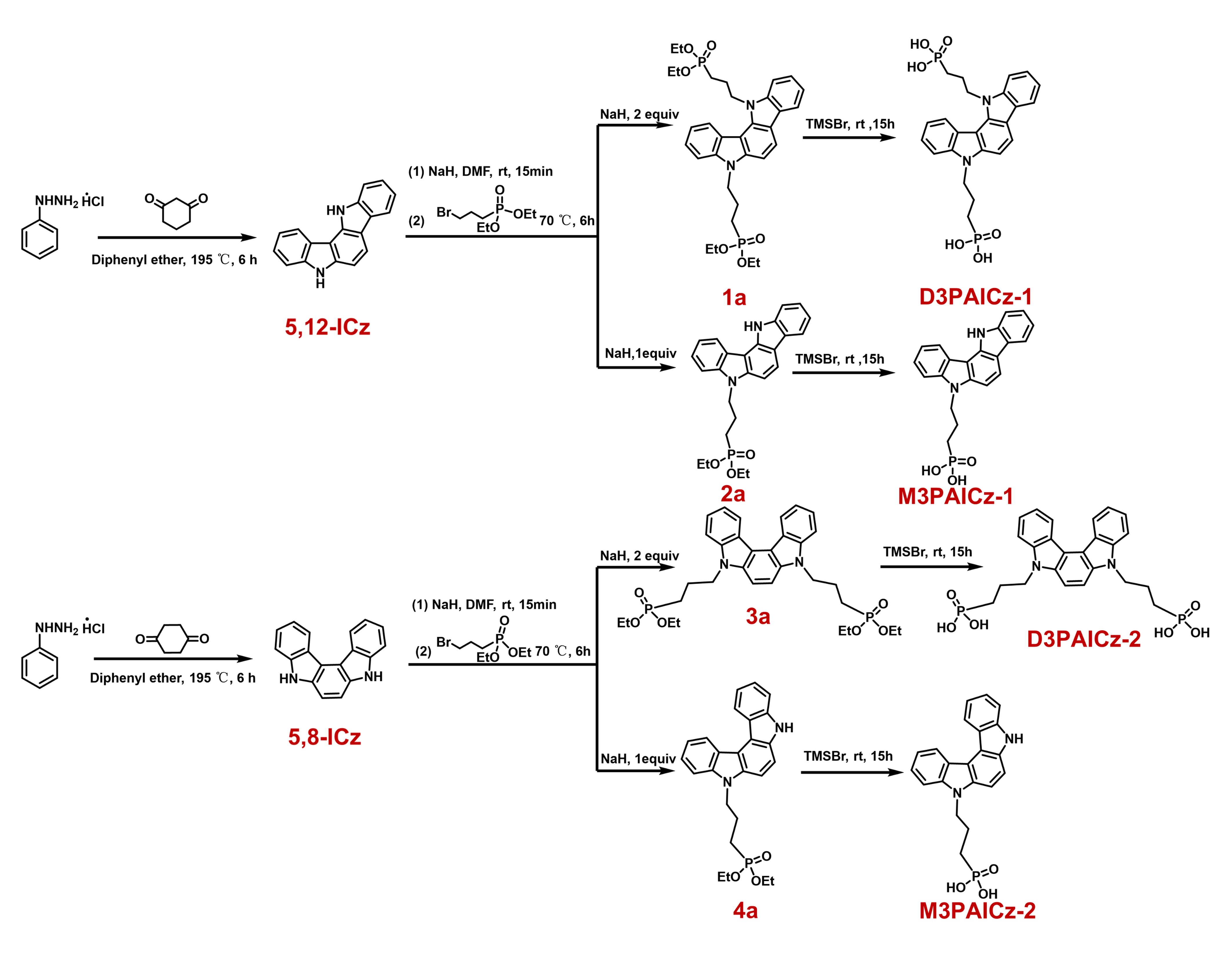


**Scheme S1.** Synthetic routes of D3PAICz-1, M3PAICz-1, D3PAICz-2, and M3PAICz-2

**Synthesis of 5,12-ICz**

1,3-Cyclohexanedione (1.50 g, 13.39 mmol) and phenylhydrazine hydrochloride (6 g, 44.49 mmol) were weighed into a two-necked reaction flask, 50 mL of diphenyl ether was added under nitrogen atmosphere, and the reaction mixture was refluxed for 6 hours at 195 °C. The reaction mixture was cooled to room temperature and the organic solvent was removed. After cooling to room temperature, the organic solvent was removed. The crude product was purified by column method using dichloro/petroleum ether (1:3) as eluent and rotary evaporation to remove the organic solvent to give a light yellow solid **5,12-ICz** (1.12 g, 32.7%). ^1^H NMR (400MHz, d6-DMSO, ppm), δ):11.72 (s, 1H), 11.52 (s, 1H), 8.64 (d, J = 7.7 Hz, 1H), 8.12 (d, J = 8.3 Hz, 1H), 7.62 (d, J = 7.9 Hz, 1H), 7.56 (d, J = 8.0 Hz, 1H), 7.41 (t, J = 7.5 Hz, 1H), 7.34 (t, J = 7.5 Hz, 1H), 7.28 (t, J = 7.4 Hz, 1H), 7.19 (t, J = 7.2 Hz, 1H).

**Synthesis of 1a**

Compound **5,12-ICz** (256 mg, 1.0 mmol) and NaH (48 mg, 2.0 mmol) were dissolved in 25 mL of anhydrous DMF and stirred under nitrogen for 15 min at room temperature. Then diethyl (3-bromopropyl) phosphonate (648 mg, 2.5 mmol) was added via syringe and heated at 70 °C for 6 h. The reaction mixture was poured into 150 mL of NaCl aqueous solution and extracted with ethyl acetate (EA) four times. The organic phase was dried with anhydrous sodium sulfate and concentrated under reduced pressure to give the crude product. The crude product was purified by silica gel chromatography (eluent, petroleum ether: ethyl acetate=1:15) to give compound **1a** (398 mg, 63.6%) as a white solid. ^1^H NMR (400MHz, d6-DMSO, ppm), δ):8.37 (d, J = 8.2 Hz, 1H), 8.30 (d, J = 8.6 Hz, 1H), 8.17 (d, J = 7.6 Hz, 1H), 7.80 (d, J = 3.6 Hz, 1H), 7.78 (d, J = 3.5 Hz, 1H), 7.60 (d, J = 8.7 Hz, 1H), 7.51 (t, J = 7.6 Hz, 1H), 7.42 (t, J = 7.6 Hz, 1H), 7.35 (t, J = 7.6 Hz, 1H), 7.25 (t, J = 7.5 Hz, 1H), 5.04 (t, J = 7.3 Hz, 2H), 4.63 (t, J = 7.2 Hz, 2H), 4.00 -3.90 (m, 4H), 3.88 - 3.76 (m, 4H), 2.14 (q, J = 8.8 Hz, 2H), 2.01 (p, J = 6.4 Hz, 2H), 1.90-1.75 (m, J = 18.3, 6.9 Hz,4H), 1.17 (t, J = 7.0 Hz, 6H), 1.02 (t, J = 7.0 Hz, 6H).^13^C NMR (101 MHz, d6-DMSO, ppm), δ):140.4, 139.8, 139.3, 135.6, 124.4, 124.0, 123.5, 122.5, 120.1, 119.6, 119.5, 119.0, 118.9, 115.9, 109.6, 109.5, 106.2, 102.5, 61.0, 24.0, 24.0, 22.7, 22.3, 21.8, 21.8, 21.3, 20.9, 16.3, 16.2, 16.1, 16.0.

**Synthesis of D3PAICz-1**

Compound **1a** (398 mg, 0.636 mmol) was dissolved in 20 mL of anhydrous dichloromethane and nitrogen was vented for 15 min at room temperature. Tribromomethylsilane (3.41 g, 22.3 mmol) was then added via syringe and stirred for 24 h. After that 15 mL of methanol was added and stirred for 6 h. The solution was concentrated under reduced pressure to about 5 ml of solution. Finally, deionised water was added slowly until a large solid was produced. Filtration and the filter cake was washed with deionised water to give **D3PAICz-1** as a white solid (203 mg, yield 63.8%). ^1^H NMR (400MHz, d_6_-DMSO, ppm), δ):8.32 (d, J = 8.3 Hz, 1H), 8.23 (d, J = 8.5 Hz, 1H), 8.13 (d, J = 7.7 Hz, 1H), 7.74 (d, J = 8.2 Hz, 2H), 7.54 (d, J = 8.6 Hz, 1H), 7.44 (t, J = 7.7 Hz, 1H), 7.38 (t, J = 8.1 Hz, 1H), 7.29 (t, J = 7.6 Hz, 1H), 7.22 (t, J = 7.4 Hz, 1H), 4.98 (t, J = 7.2 Hz, 2H), 4.56 (t, J = 7.1 Hz, 2H), 2.16 (d, J = 6.5 Hz, 2H), 2.00 (d, J = 28.4 Hz, 2H), 1.61-1.49 (m, 4H).^13^C NMR (101 MHz, d6-DMSO, ppm), δ):140.5, 139.9, 139.4, 135.8, 124.3, 123.9, 123.6, 122.5, 120.2, 119.5, 119.4, 118.9, 118.7, 115.7, 109.7, 109.6, 106.1, 102.5, 25.7, 25.5, 25.2, 25.2, 25.0, 24.8, 22.8, 22.8. ^31^P NMR (162 MHz, d6-DMSO, ppm), δ):25.665(s,1P), 25.176(s,1P). HR-MS(ESI) *m/z* calc. for C_24_H_26_N_2_O_6_P_2_: calcd: 500.1266; found:499.1191 [M-H]^-^.

**Synthesis of 2a**

Compound **5,12-ICz** (256 mg, 1.0 mmol) and NaH (24 mg, 1.0 mmol) were dissolved in 25 mL of anhydrous DMF and stirred under nitrogen for 15 min at room temperature. then diethyl (3-bromopropyl) phosphonate (259 mg, 1.0 mmol) was added via syringe and heated at 70 °C for 6 h. The reaction mixture was poured into 150 mL of NaCl aqueous solution and extracted with ethyl acetate (EA) four times. The organic phase was dried with anhydrous sodium sulfate and concentrated under reduced pressure to give the crude product. The crude product was purified by silica gel chromatography (eluent, petroleum ether: ethyl acetate=1:8) to give compound **2a** (271 mg, 62.4%) as a white solid. ^1^H NMR (400MHz, d6-DMSO, ppm), δ):11.80 (s, 1H), 8.71 (d, J = 7.7 Hz, 1H), 8.21 (d, J = 8.5 Hz, 1H), 8.13 (d, J = 7.7 Hz, 1H), 7.74 (d, J = 8.2 Hz, 1H), 7.65 (d, J = 8.0 Hz, 1H), 7.54 -7.44 (m, 2H), 7.35 (q, J = 7.5 Hz, 2H), 7.21 (t, J = 7.4 Hz, 1H), 4.59 (t, J = 7.0 Hz, 2H), 3.93 (d, J = 18.1 Hz, 4H), 2.02 (d, J = 27.6 Hz, 2H), 1.81 (d, J = 33.7 Hz, 2H), 1.16 (t, J = 7.0 Hz, 6H).^13^C NMR (101 MHz, d6-DMSO, ppm), δ):139.4, 138.9, 133.8, 124.4, 123.7, 123.5, 121.4, 121.0, 119.0, 119.0, 118.5, 115.0, 111.1, 109.1, 106.1, 101.7, 61.0, 61.0, 22.7, 22.1, 22.1, 21.3, 16.3, 16.2.

**Synthesis of M3PAICz-1**

Compound **2a** (271 mg, 0.624 mmol) was dissolved in 20 mL of anhydrous dichloromethane and nitrogen was vented for 15 min at room temperature. Tribromomethylsilane (1.67 g, 10.9 mmol) was then added via syringe and stirred for 24 h. After that 15 mL of methanol was added and stirred for 6 h. The solution was concentrated under reduced pressure to about 5 ml of solution. Finally, deionised water was added slowly until a large solid was produced. Filtration and the filter cake was washed with deionised water to give **M3PAICz-1** as a white solid (162 mg, 68.6%). ^1^H NMR (400MHz, d6-DMSO, ppm), δ): 11.78 (s, 1H), 8.69 (d, J = 7.8 Hz, 1H), 8.19 (d, J = 8.6 Hz, 1H), 8.12 (d, J = 7.8 Hz, 1H), 7.74 (d, J = 8.2 Hz, 1H), 7.63 (d, J = 8.1 Hz, 1H), 7.53 -7.44 (m, 2H), 7.34 (q, J = 8.0 Hz, 2H), 7.20 (t, J = 7.4 Hz, 1H), 4.59 (s, 2H), 2.04 (d, J = 27.9 Hz, 2H), 1.60 (d, J = 33.9 Hz, 2H).^13^C NMR (101 MHz, d6-DMSO, ppm), δ:)139.5, 139.4, 139.0, 133.8, 124.4, 123.7, 123.5, 121.4, 121.0, 119.0, 119.0, 118.9, 118.5, 115.0, 111.1, 109.3, 106.1, 101.9, 25.8, 24.4, 22.9. ^31^P NMR (162 MHz, d6-DMSO, ppm) δ:24.397(s,1P). HR-MS(ESI) *m/z* calc. for C_21_H_19_N_2_O_3_P: calcd: 378.1133; found:377.1058 [M-H]^-^.

**Synthesis of 5,8-ICz**

1,4-Cyclohexanedione (1.50 g, 13.39 mmol) and phenylhydrazine hydrochloride (6 g, 44.49 mmol) were weighed into a two-necked reaction flask, 50 mL of diphenyl ether was added under a nitrogen atmosphere, and the reaction mixture was refluxed for 6 hours at 195 °C. After cooling to room temperature, the organic solvent was removed. The crude product was purified by column method using dichloro/petroleum ether (1:3) as eluent and rotary evaporation to remove the organic solvent to give a pale yellow solid **5,8-ICz** (0.79 g,23.1%). ^1^H NMR (400MHz, d6-DMSO, ppm), δ): 11.42 (s, 2H), 8.66 (d, J = 7.9 Hz, 2H), 7.59 (s, 2H), 7.55 (d, J = 8.0 Hz, 2H), 7.38 (t, J = 7.4 Hz, 2H), 7.26 (t, J = 6.9 Hz, 2H).

**Synthesis of 3a**

Compound **5,8-ICz** (256 mg, 1.0 mmol) and NaH (48 mg, 2.0 mmol) were dissolved in 25 mL of anhydrous DMF and stirred under nitrogen for 15 min at room temperature. Then diethyl (3-bromopropyl) phosphonate (648 mg, 2.5 mmol) was added via syringe and heated at 70 °C for 6 h. The reaction mixture was poured into 150 mL of NaCl aqueous solution and extracted with ethyl acetate (EA) four times. The organic phase was dried with anhydrous sodium sulfate and concentrated under reduced pressure to give the crude product. The crude product was purified by silica gel chromatography (eluent: petroleum ether: ethyl acetate=1:15) to give compound **3a** (359 mg, 57.4%) as a white solid. ^1^H NMR (400MHz, d6-DMSO, ppm), δ):8.80 (d, J = 8.1 Hz, 2H), 7.92 (s, 2H), 7.79 (d, J = 8.2 Hz, 2H), 7.54 (t, J = 7.7 Hz, 2H), 7.38 (t, J = 7.3 Hz, 2H), 4.64 (t, J = 7.0 Hz, 4H), 3.93 (d, J = 19.1 Hz, 8H), 2.02 (d, J = 27.3 Hz, 4H), 1.80 (d, J = 33.6 Hz, 4H), 1.16 (t, J = 7.1 Hz, 12H).^13^C NMR (101 MHz, d6-DMSO, ppm), δ:)139.7, 135.0, 125.0, 123.0, 121.5, 118.7, 115.3, 109.4, 108.5, 61.1, 61.0, 22.7, 22.1, 22.0, 21.3, 16.3, 16.2.

**Synthesis of D3PAICz-2**

Compound **3a** (359 mg, 0.574 mmol) was dissolved in 20 mL of anhydrous dichloromethane and nitrogen was vented for 15 min at room temperature. tribromomethylsilane (3.08 g, 20.1 mmol) was then added via syringe and stirred for 24 h. After that, 15 mL of methanol was added and stirred for 6 h. The solution was concentrated under reduced pressure to about 5 ml of solution. Finally, deionized water was added slowly until a large solid was produced. Filtration was performed and the filter cake was washed with deionized water to give **D3PAICz-2** as a white solid (187 mg, 65.1%). ^1^H NMR (400MHz, d6-DMSO, ppm), δ):8.79 (d, *J* = 8.1 Hz, 2H), 7.91 (s, 2H), 7.79 (d, *J* = 8.2 Hz, 2H), 7.53 (t, *J* = 7.6 Hz, 2H), 7.37 (t, *J* = 7.5 Hz, 2H), 4.65 (t, *J* = 7.0 Hz, 4H), 2.03 (d, *J* = 27.5 Hz, 4H), 1.59 (d, *J* = 33.9 Hz, 4H)。^13^C NMR (101 MHz, d6-DMSO, ppm), δ):139.9, 135.1, 125.0, 123.0, 121.6, 118.7, 115.4, 109.6, 108.7, 25.8, 24.4, 22.9. ^31^P NMR (162 MHz, d6-DMSO, ppm), δ):26.221(s,1P). HR-MS(ESI) *m/z* calc. for C_24_H_26_N_2_O_6_P_2_: calcd: 500.1266; found:499.1193 [M-H].

**Synthesis of 4a**

Compound **5,8-ICz** (256 mg, 1.0 mmol) and NaH (24 mg, 1.0 mmol) were dissolved in 25 mL of anhydrous DMF and stirred under nitrogen for 15 min at room temperature. then diethyl (3-bromopropyl) phosphonate (259 mg, 1.0 mmol) was added via syringe and heated at 70 °C for 6 h. The reaction mixture was poured into 150 mL of NaCl aqueous solution and extracted with ethyl acetate (EA) four times. The organic phase was dried with anhydrous sodium sulfate and concentrated under reduced pressure to give the crude product. The crude product was purified by silica gel chromatography (eluent: petroleum ether: ethyl acetate=1:8) to afford compound **4a** (283 mg, 65.1%) as a white solid. ^1^H NMR (400MHz, d6-DMSO, ppm), δ):11.53 (s, 1H), 8.77 (d, J = 8.0 Hz, 1H), 8.74 (d, J = 7.9 Hz, 1H), 7.83 (d, J = 8.7 Hz, 1H), 7.78 (d, J = 8.0 Hz, 1H), 7.72 (d, J = 8.6 Hz, 1H), 7.62 (d, J = 8.1 Hz, 1H), 7.52 (t, J = 7.5 Hz, 1H), 7.46 (t, J = 7.4 Hz, 1H), 7.36 (t, J = 7.4 Hz, 1H), 7.32 (t, J = 7.4 Hz, 1H), 4.63 (t, J = 7.4 Hz, 2H), 3.96-3.90 (m, 4H), 2.03-1.99 (m, 2H), 1.83-1.78 (m, 2H), 1.17 (t, J = 7.0 Hz, 6H). ^13^C NMR (101 MHz, DMSO), δ): 139.6, 139.5, 134.9, 134.7, 124.7, 122.8, 122.7, 121.8, 121.7, 118.6, 118.4, 115.5, 115.1, 111.2, 110.4, 109.3, 108.4, 61.0, 61.0, 22.7, 22.1, 22.1, 21.3, 16.3, 16.2.

**Synthesis of M3PAICz-2**

Compound **4a** (283 mg, 0.651 mmol) was dissolved in 20 mL of anhydrous dichloromethane and nitrogen was vented for 15 min at room temperature. tribromomethylsilane (1.74 g, 11.4 mmol) was then added via a syringe and stirred for 24 h. After that, 15 mL of methanol was added and stirred for 6 h. The solution was concentrated under reduced pressure to about 5 ml of solution. Finally, deionized water was added slowly until a large solid was produced. Filtration was performed and the filter cake was washed with deionized water to give **M3PAICz-2** as a white solid (177 mg, 71.9% ).^1^H NMR (400MHz, d6-DMSO, ppm), δ):11.55 (s, 1H), 8.80-8.72 (m, 2H), 7.83 (d, J = 8.8 Hz, 1H), 7.78 (d, J = 8.3 Hz, 1H), 7.72 (d, J = 8.7 Hz, 1H), 7.62 (d, J = 8.1 Hz, 1H), 7.51 (t, J = 7.6 Hz, 1H), 7.46 (t, J = 7.6 Hz, 1H), 7.34 (dt, J = 14.6, 7.5 Hz, 2H), 4.63 (t, J = 7.0 Hz, 2H), 2.10-1.96 (m, 2H), 1.66-1.53 (m, 2H).^13^C NMR (101 MHz, DMSO), δ): 139.7, 139.6, 134.9, 134.9, 124.7, 122.9, 122.8, 122.0, 121.7, 118.6, 118.4, 115.6, 115.2, 111.3, 110.5, 109.5, 108.6, 39.5, 25.8, 24.4,22.9.^31^P NMR (162 MHz, d6-DMSO, ppm), δ):25.583(s,1P). HR-MS(ESI) *m/z* calc. for C_21_H_19_N_2_O_3_P: calcd: 378.1133; found:377.1059 [M-H].

**Synthesis of MAPbBr_3_ single crystals**

The synthesis method of single-crystal MAPbBr_3_ is as follows: 2.4 g of MABr, 7.7 g of PbBr_2_, and 15 mL of DMF were mixed in a closed glass bottle and stirred overnight at room temperature (20 ℃). After complete dissolution, the colorless solution was filtered by 0.45 μm filters, moved to a 30 mL glass bottle, and heated on a hot plate (40 ℃ for 1 h, 70 ℃ for 1 h, 100 ℃ for 1 h, 110 °C for 1 h and 120 °C for 1 h). The bright orange nanocubes (MAPbBr_3_) were grown in DMF solution, dried on a hot plate at 80 °C for 2 h in a nitrogen glove box and collected in transparent glass bottles (the yield of MAPbBr_3_ was ~50%). The phase purity of MAPbBr_3_ was verified by XRD.

1. **Figures and Tables**


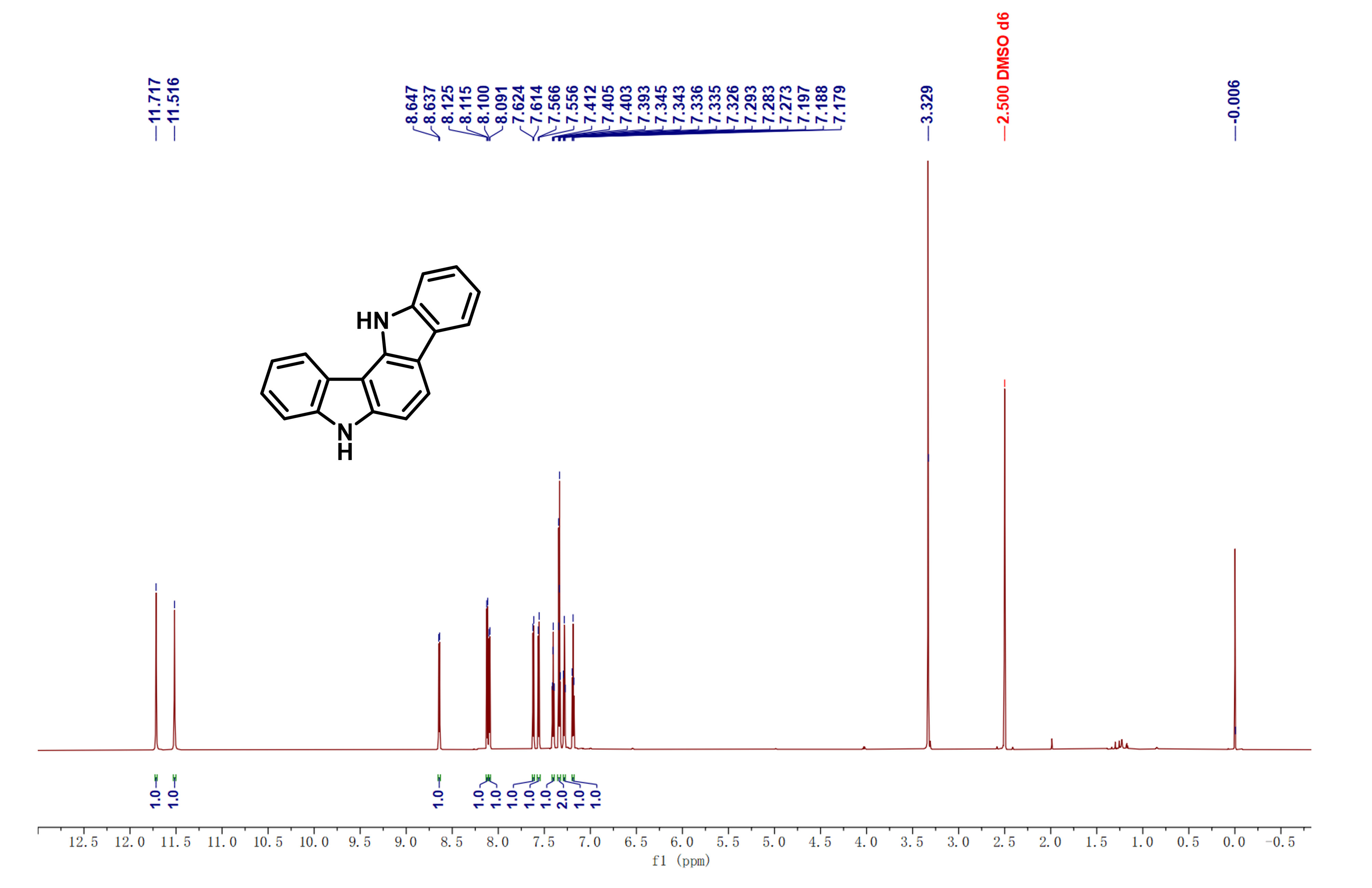


**Figure S1.** ^1^H NMR spectrum of compound **5,12-ICz** in DMSO-*d*_6_.


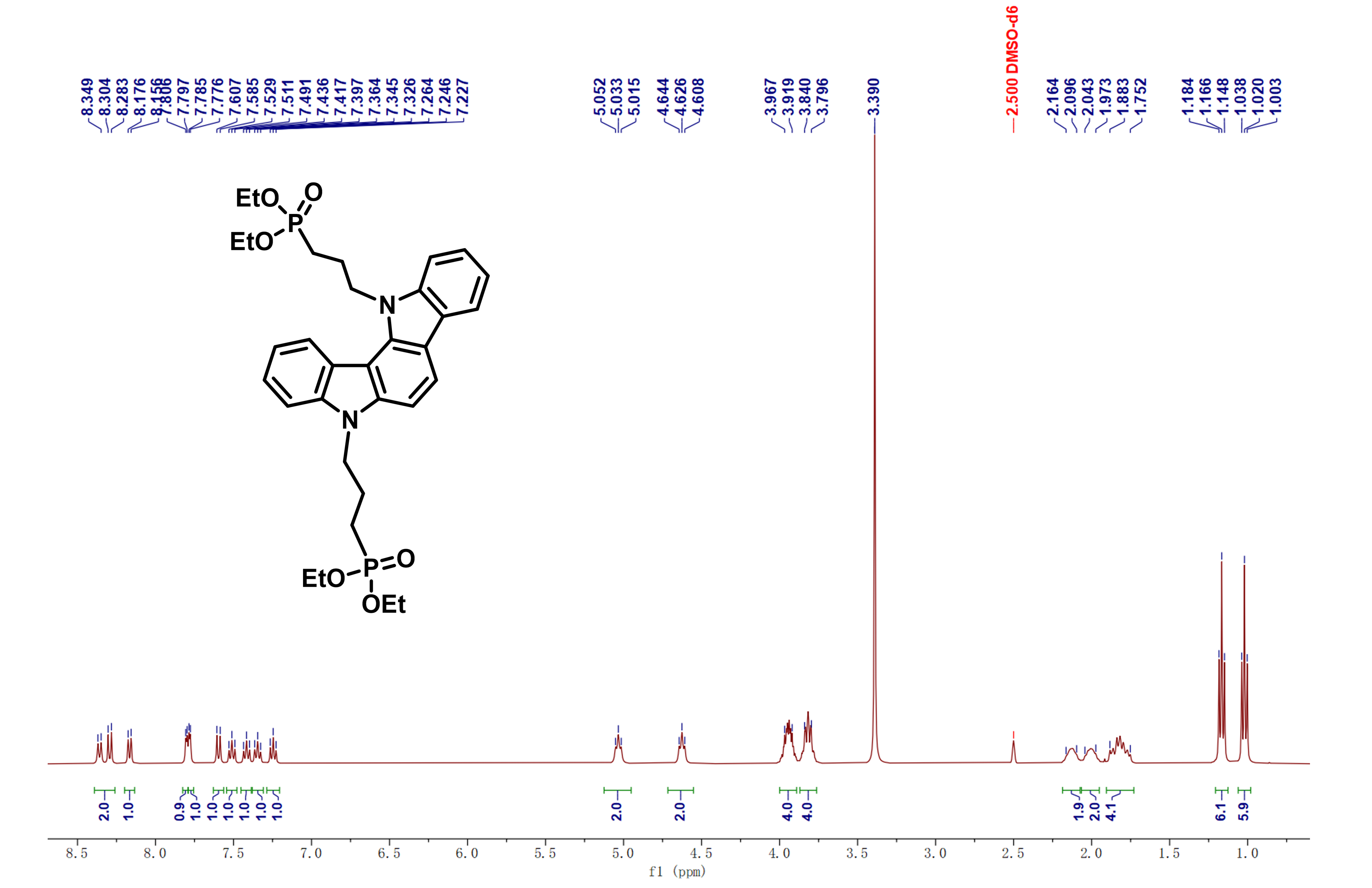


**Figure S2.** ^1^H NMR spectrum of compound **1a** in DMSO-*d*_6_.


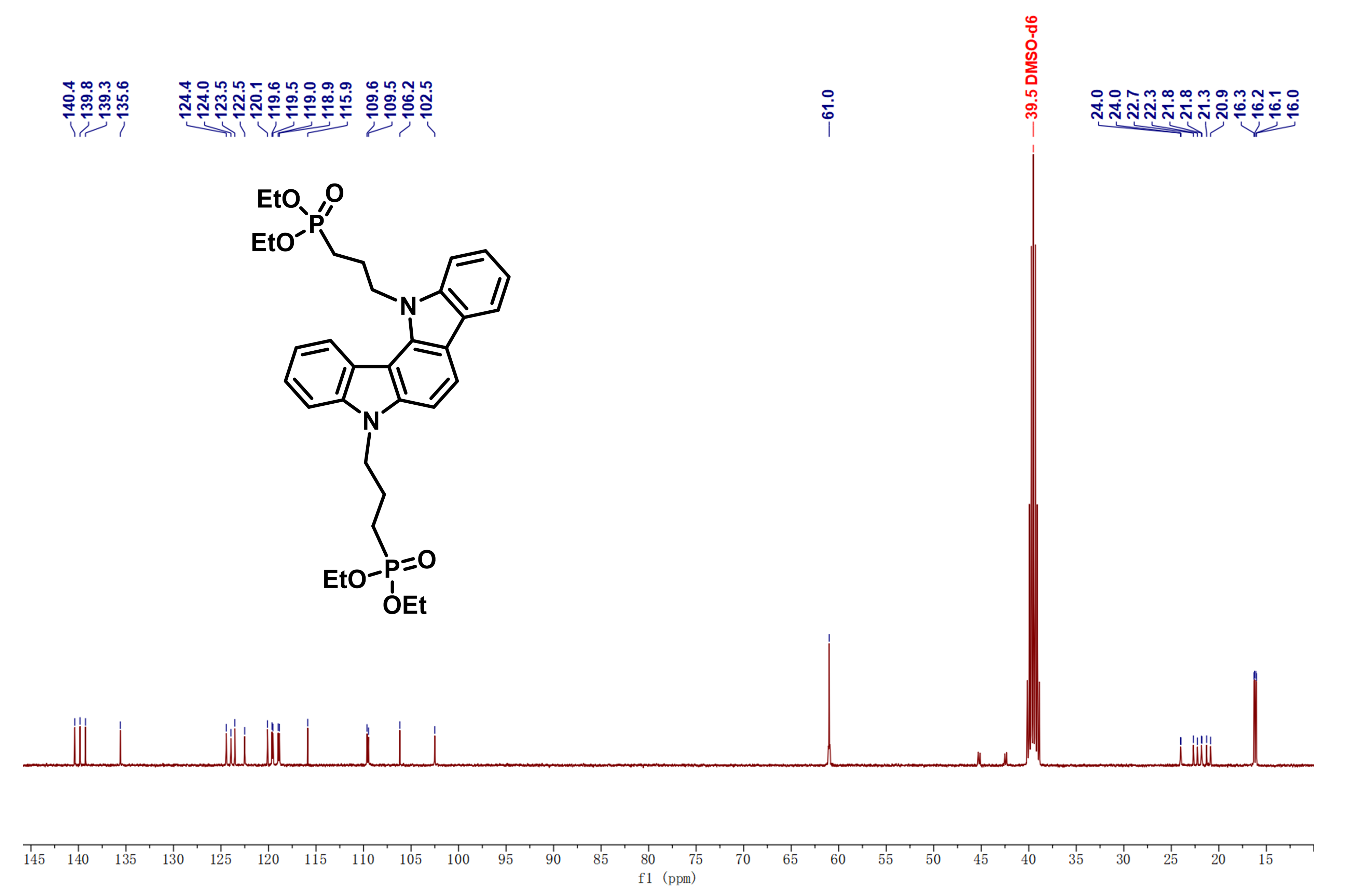


**Figure S3.** ^13^C NMR spectrum of compound **1a** in DMSO-*d*_6_.


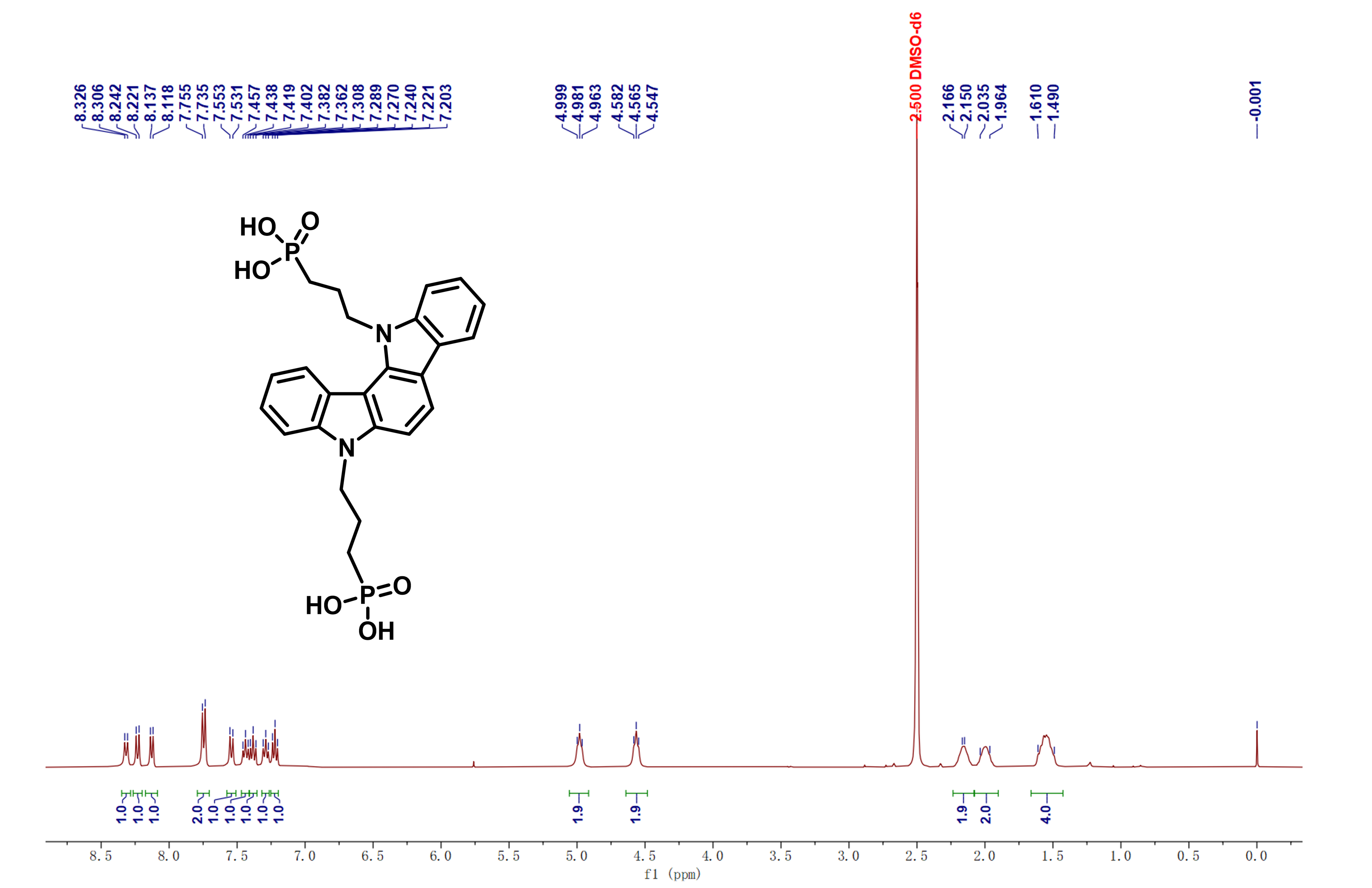


**Figure S4.** ^1^H NMR spectrum of compound **D3PAICz-1** in DMSO-*d*_6_.


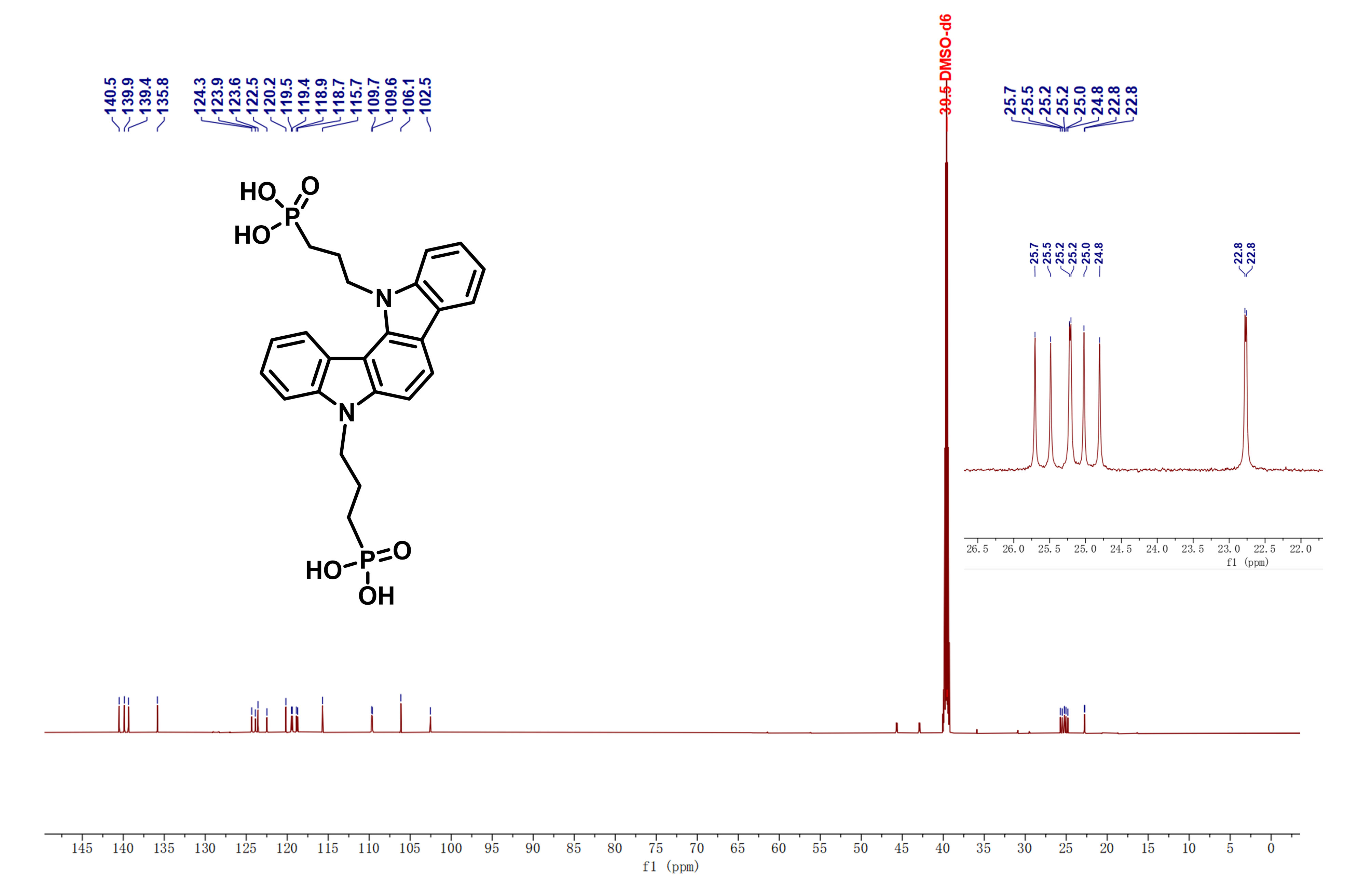


**Figure S5.** ^13^C NMR spectrum of compound **D3PAICz-1** in DMSO-*d*_6_.


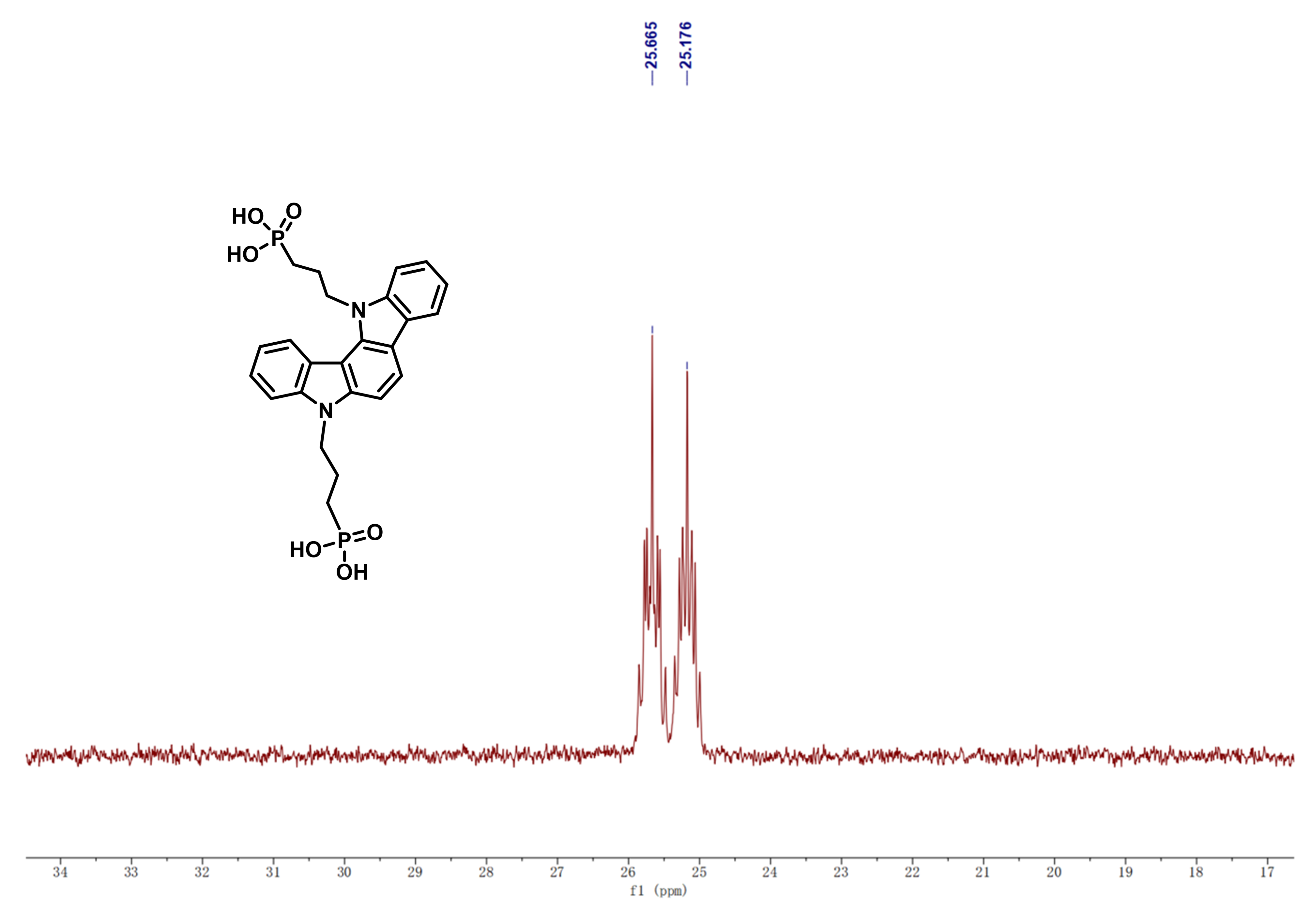


**Figure S6.** ^31^P NMR spectrum of compound **D3PAICz-1** in DMSO-*d*_6_.


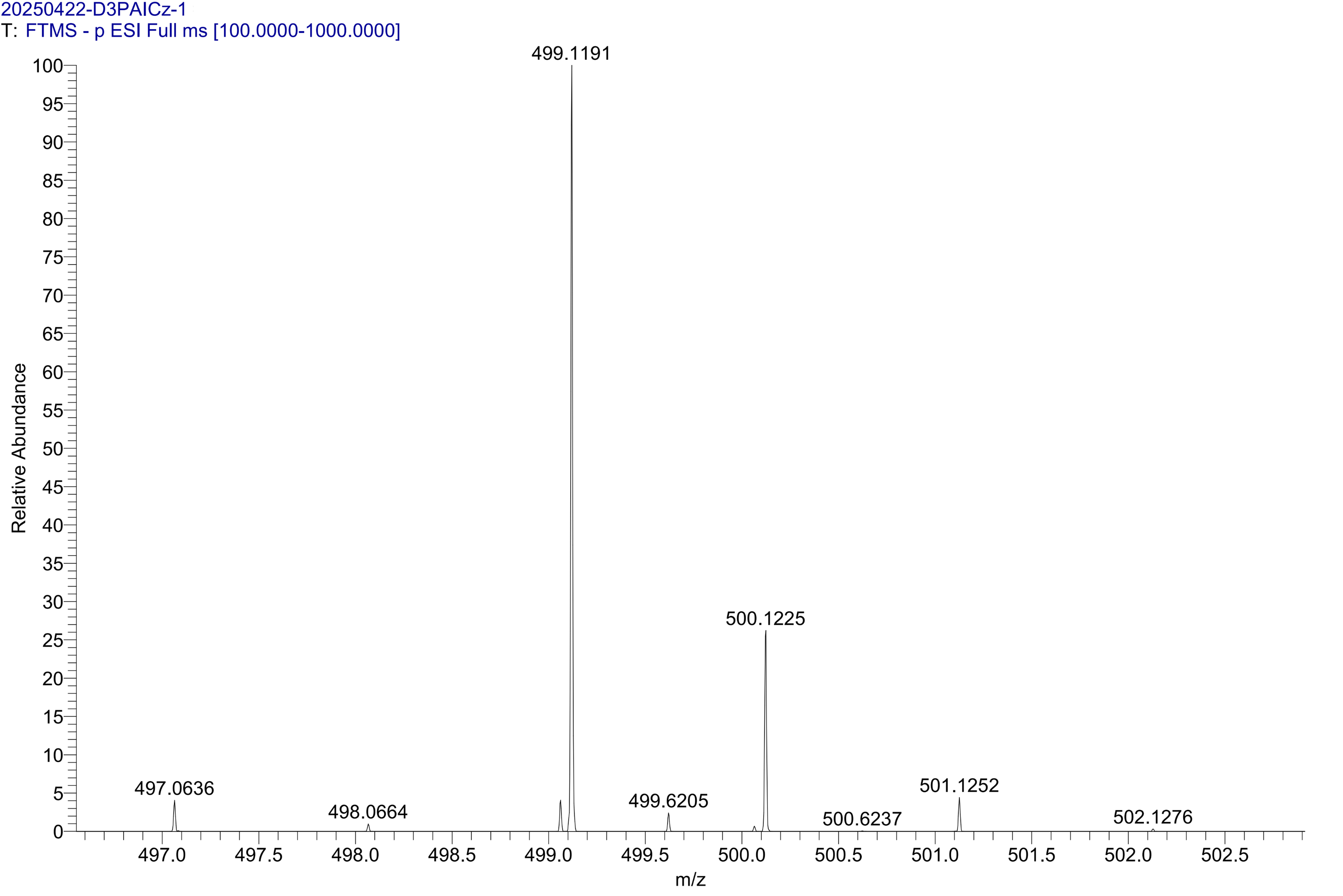


**Figure S7.** HRMS spectrum of **D3PAICz-1**


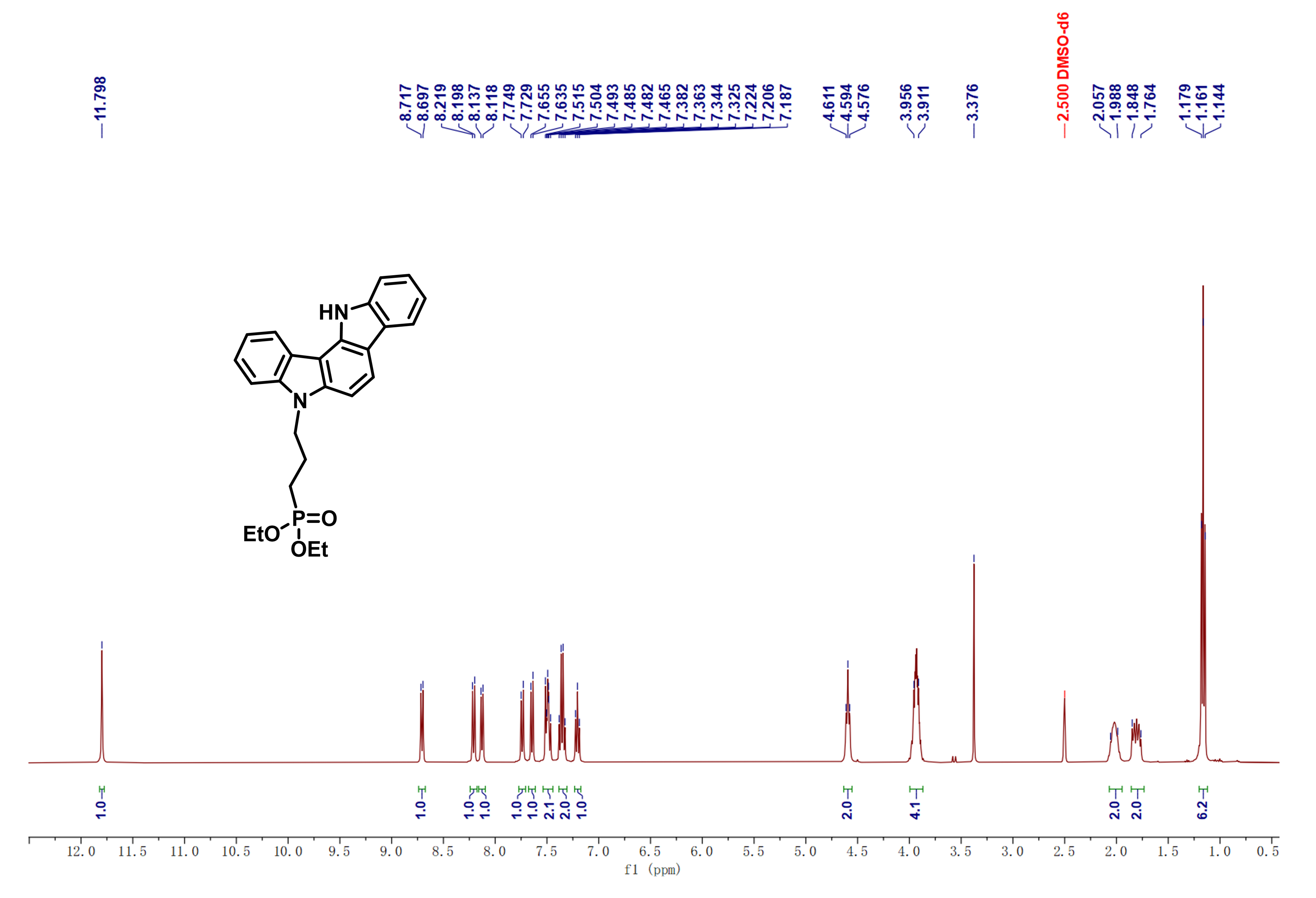


**Figure S8.** ^1^H NMR spectrum of compound **2a** in DMSO-*d*_6_.


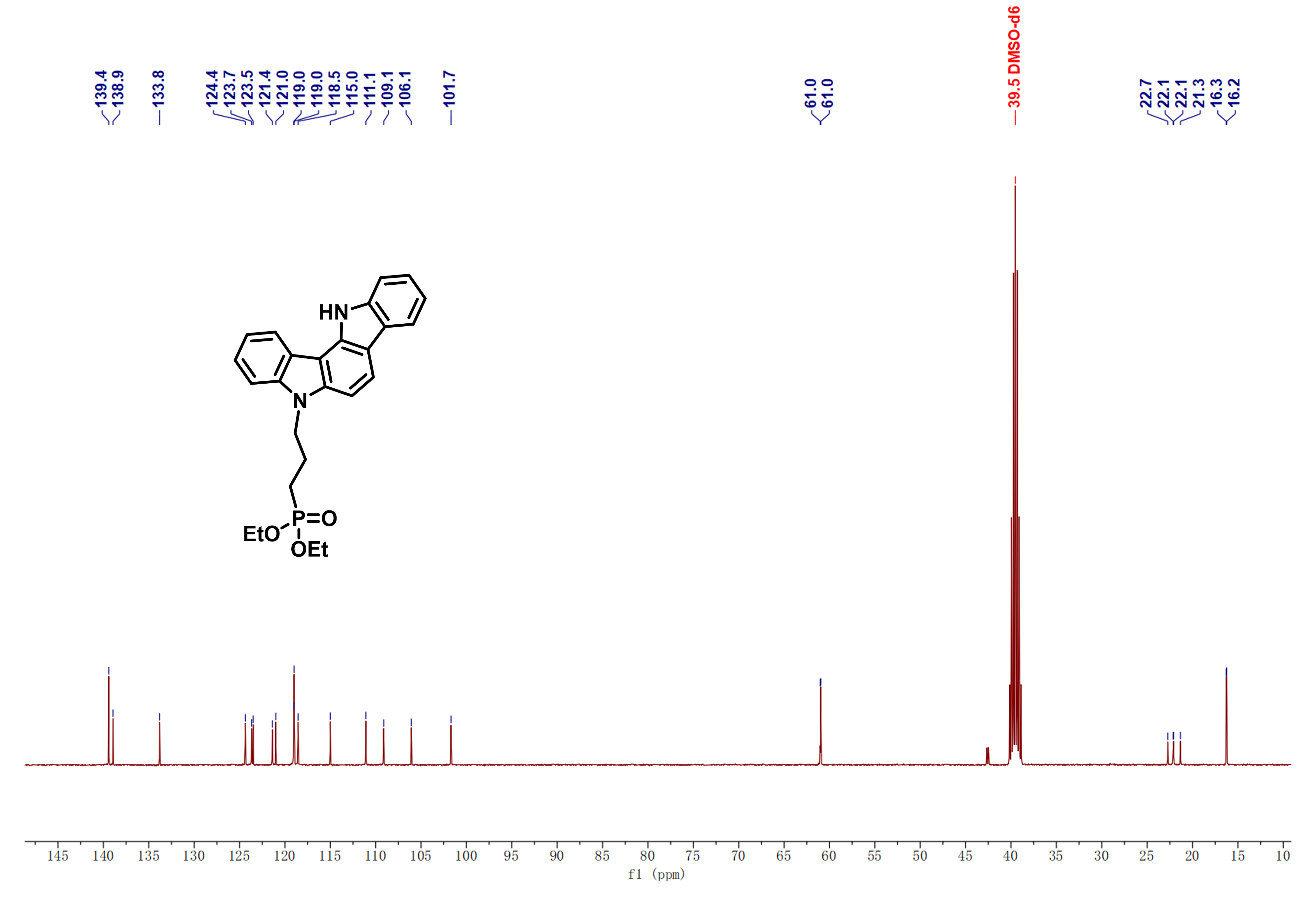


**Figure S9.** ^13^C NMR spectrum of compound **2a** in DMSO-*d*_6_.


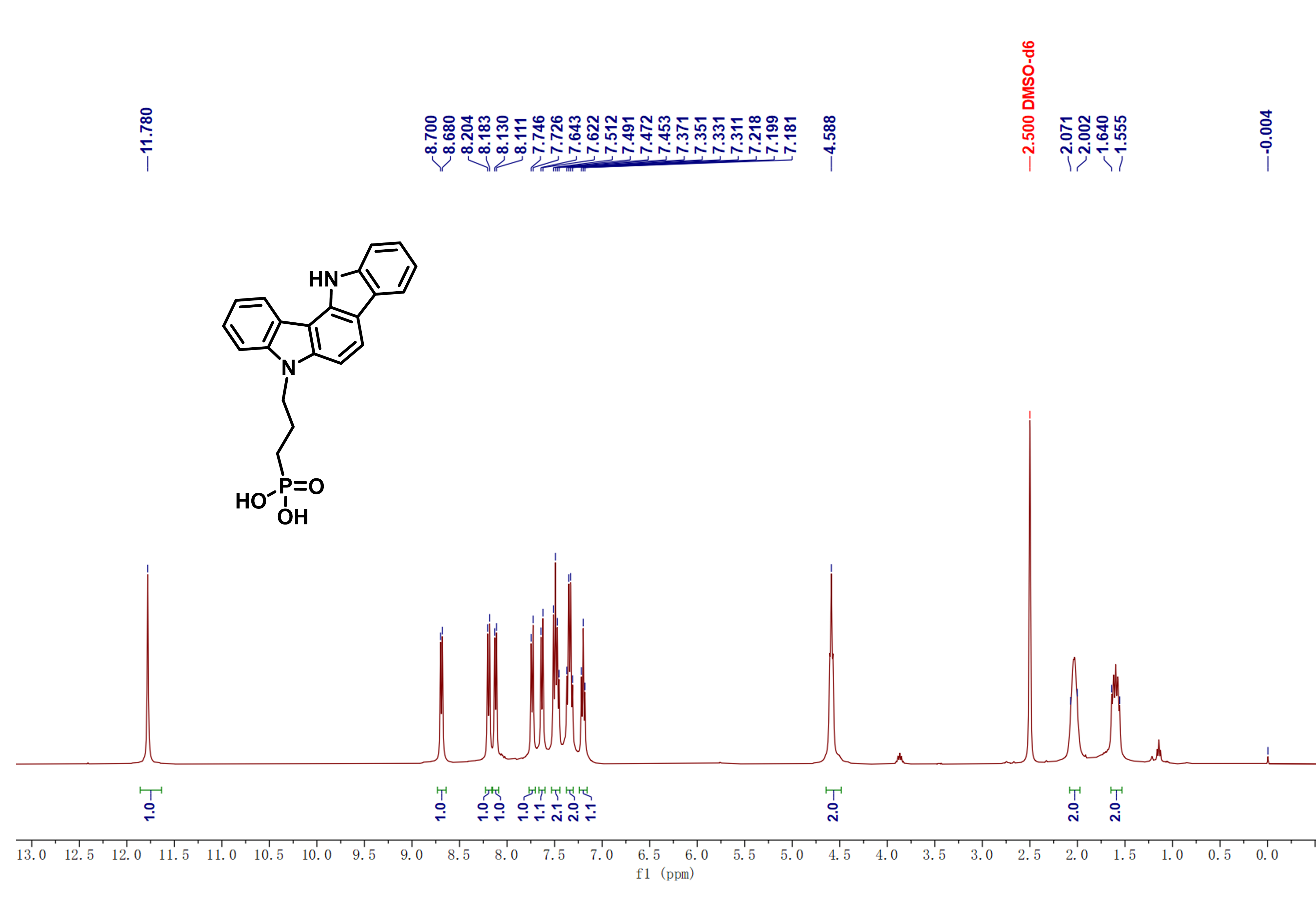


**Figure S10.** ^1^HNMR spectrum of compound **M3PAICz-1** in DMSO-*d*_6_.


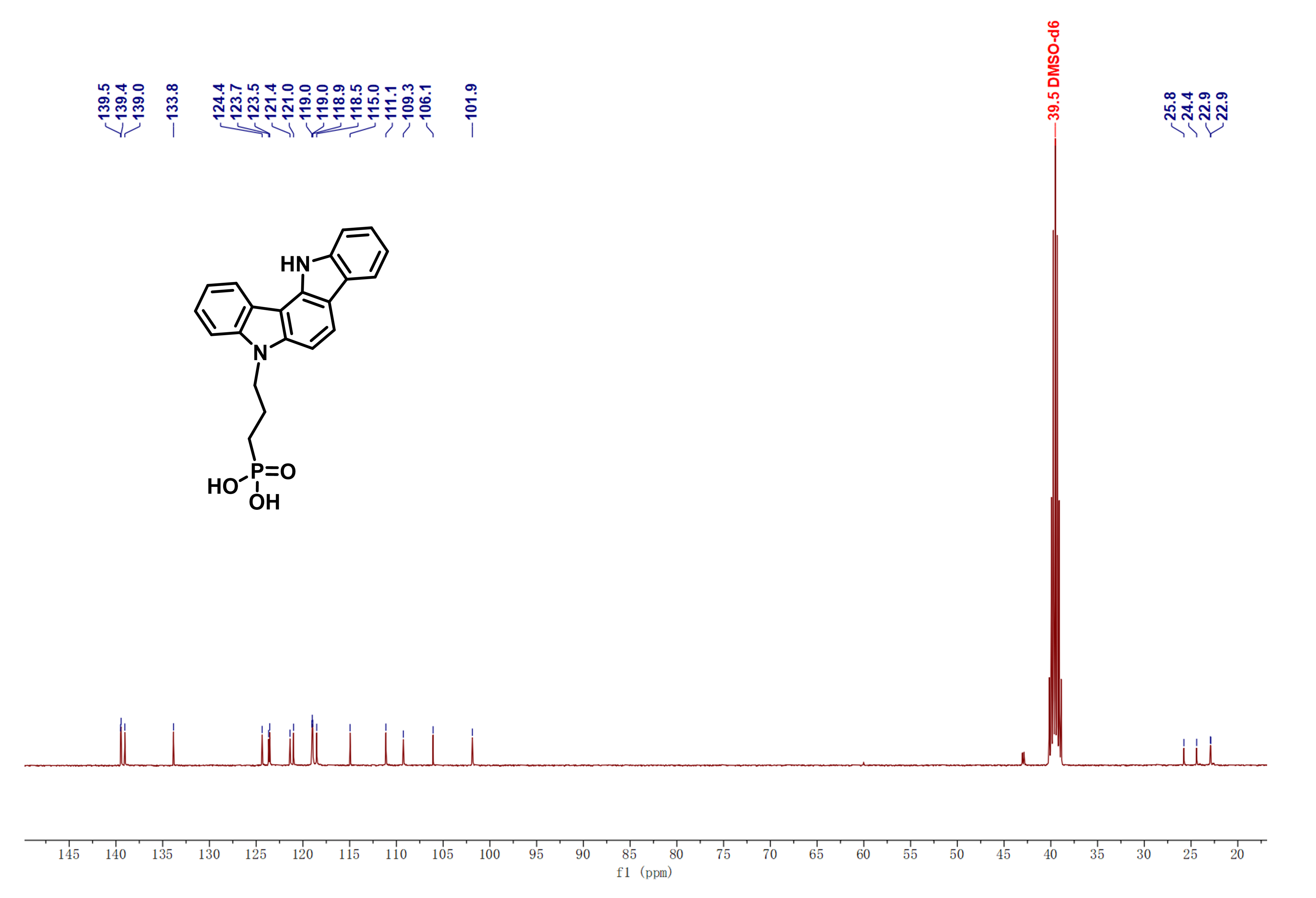


**Figure S11.** ^13^C NMR spectrum of compound **M3PAICz-1** in DMSO-*d*_6_.


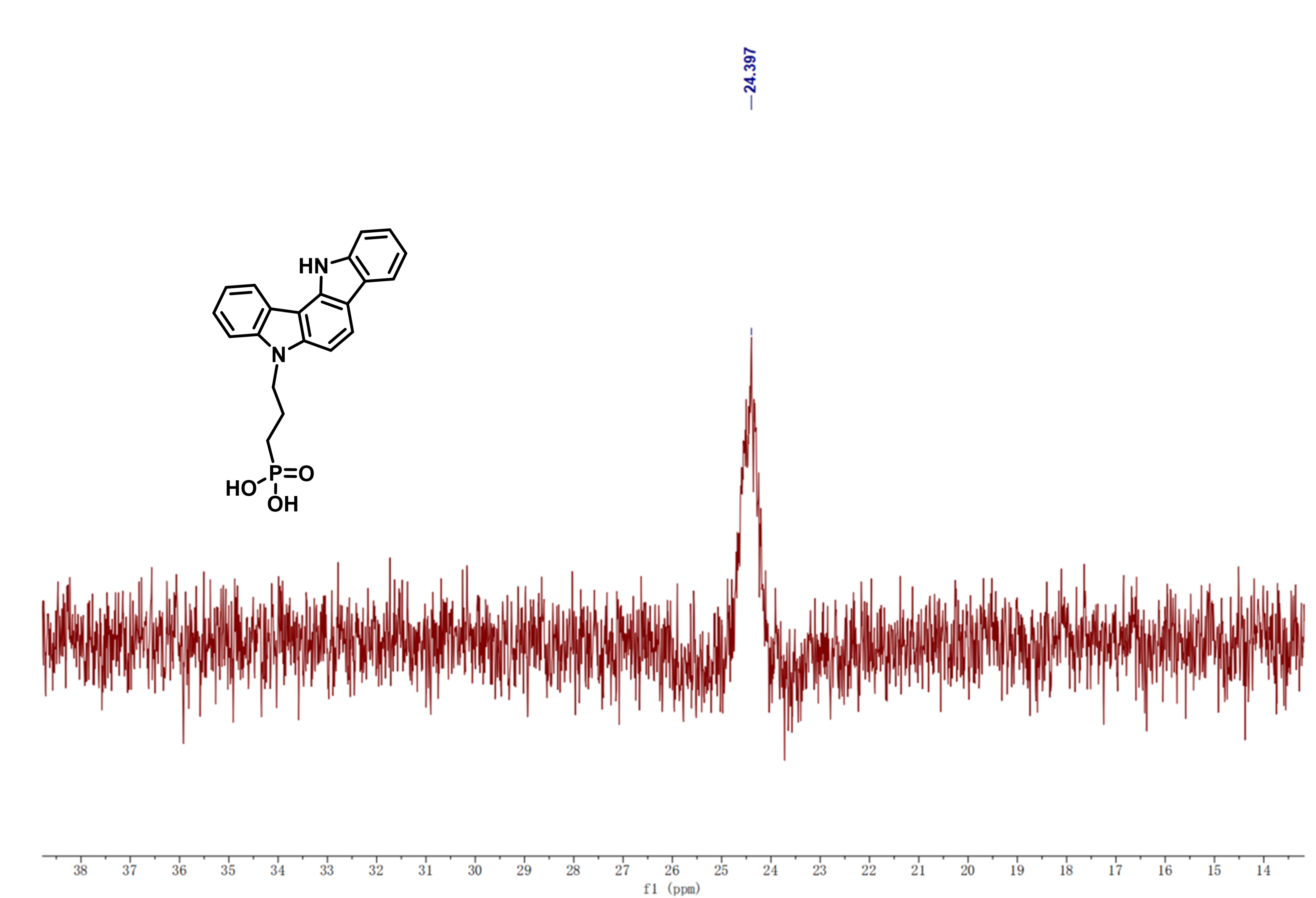


**Figure S12.** ^31^P NMR spectrum of compound **M3PAICz-1** in DMSO-*d*_6_.


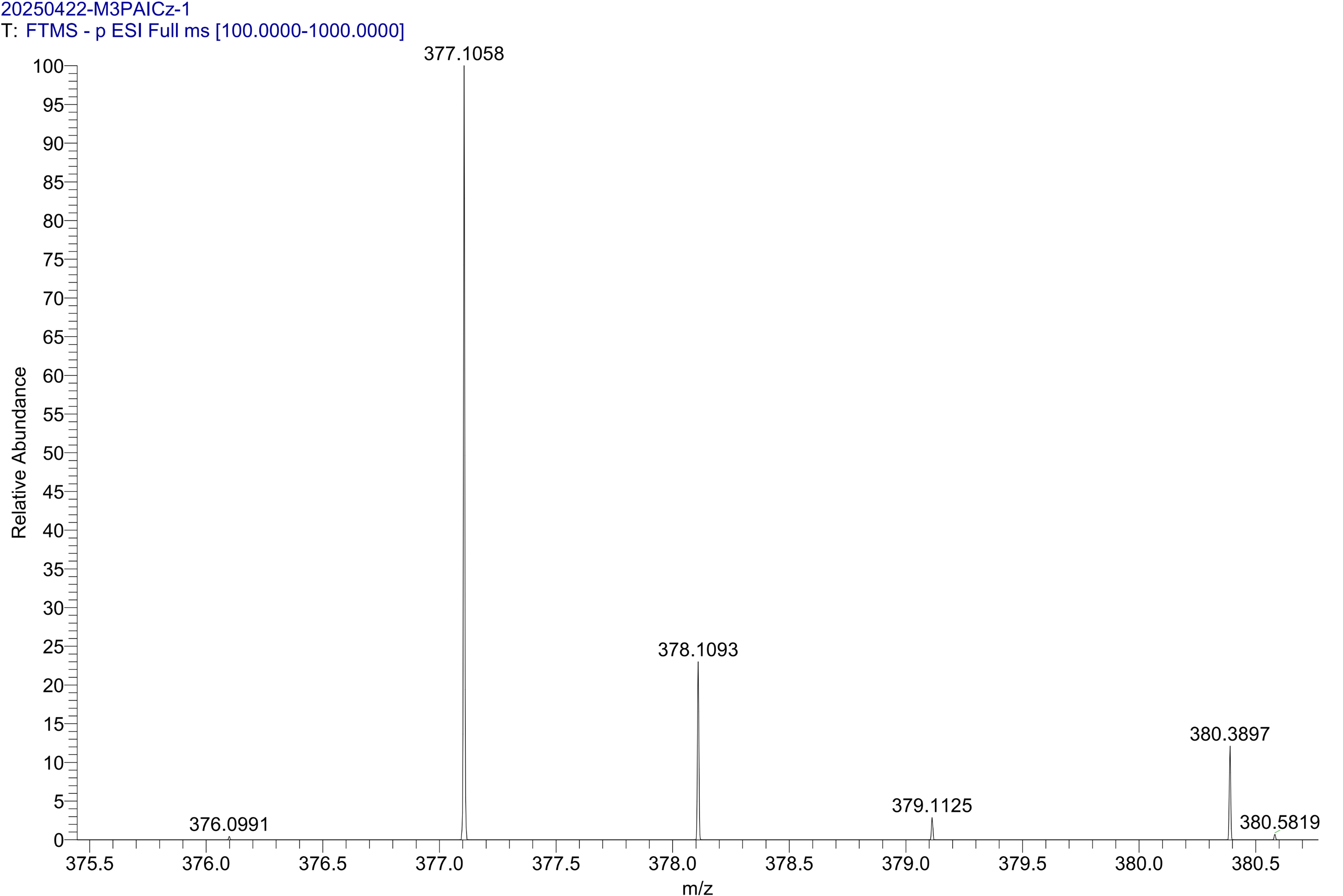


**Figure S13.** HRMS spectrum of **M3PAICz-1**


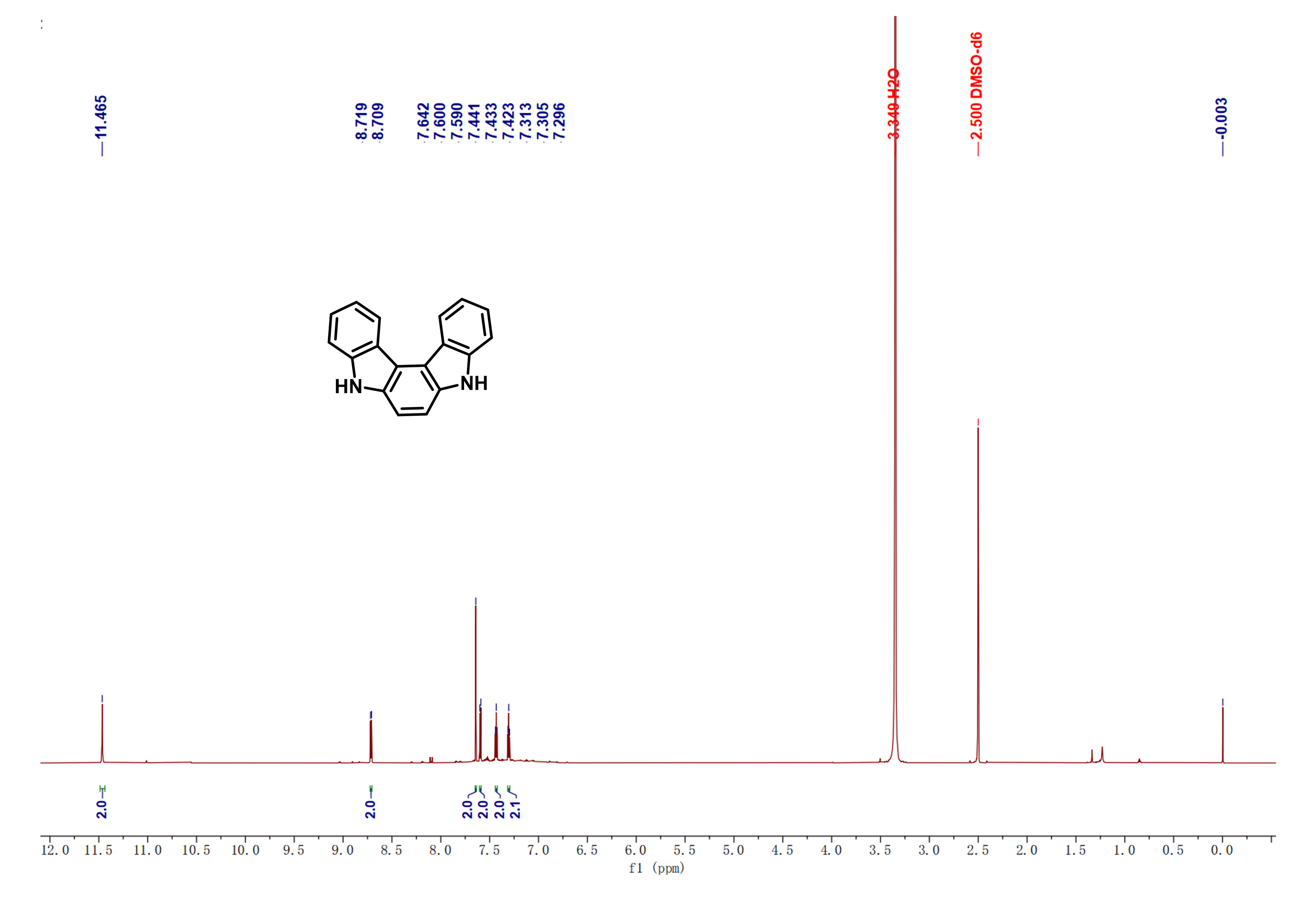


**Figure S14.** ^1^HNMR spectrum of compound **5,8-ICz** in DMSO-*d*_6_.


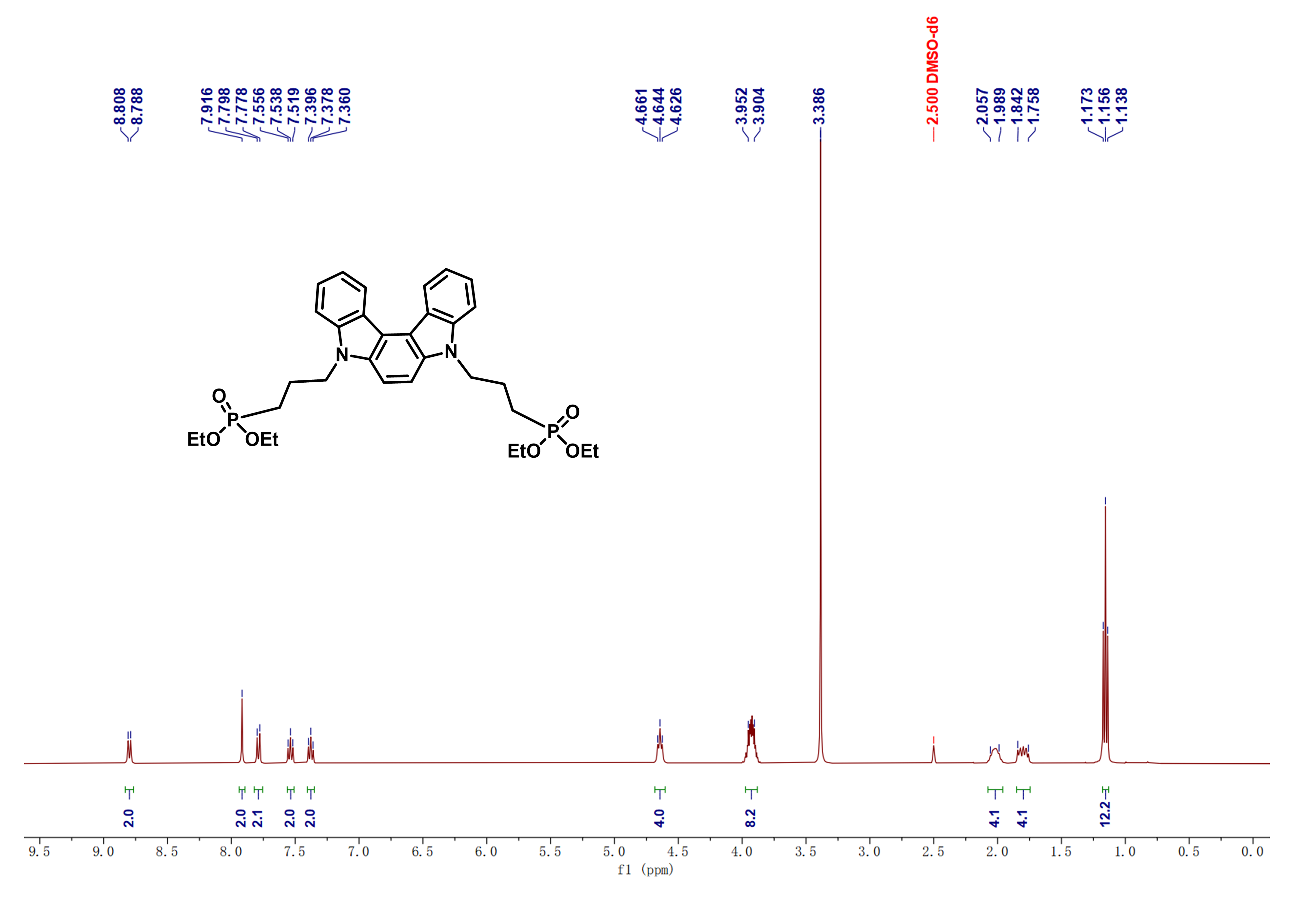


**Figure S15.** ^1^HNMR spectrum of compound **3a** in DMSO-*d*_6_.


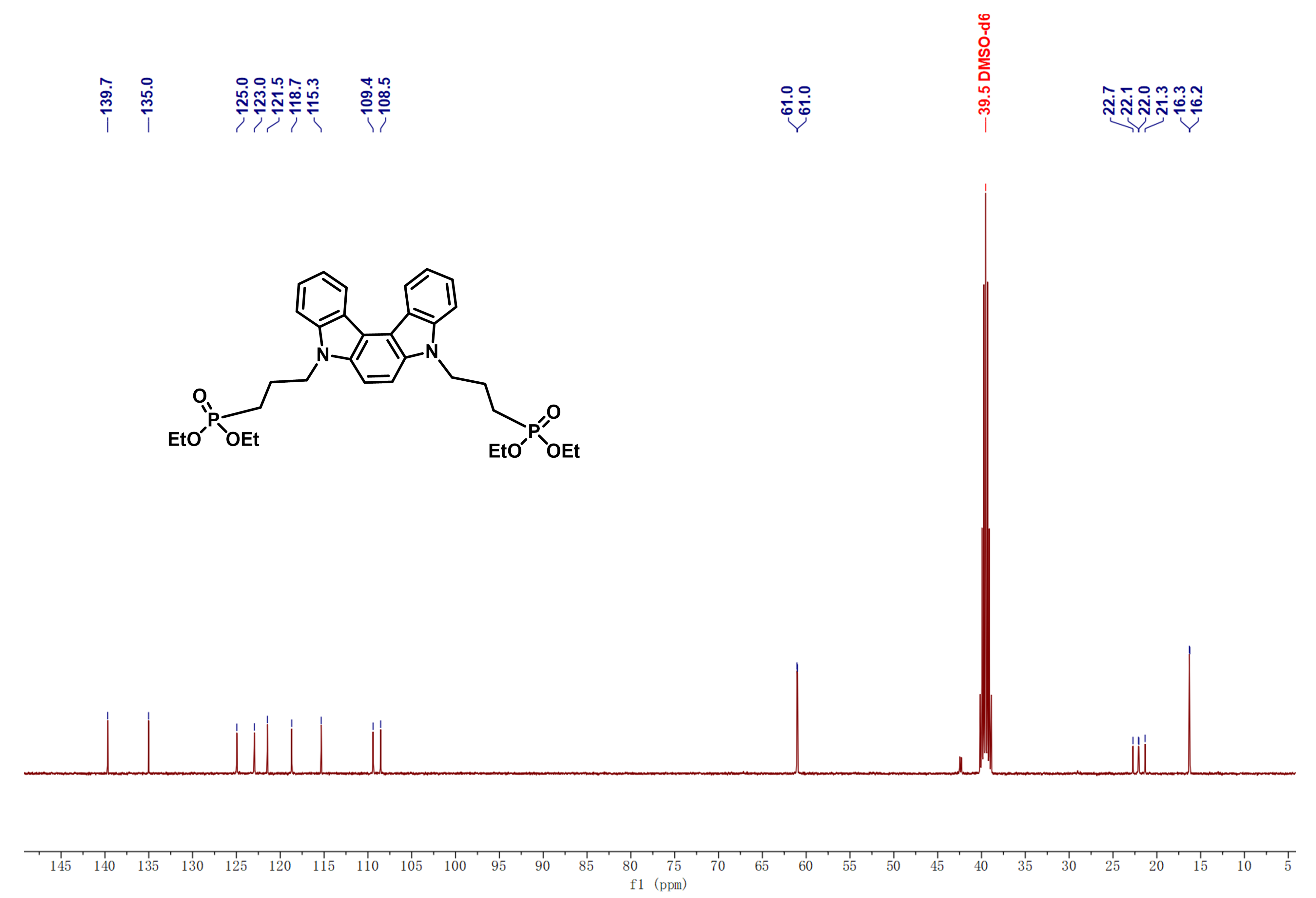


**Figure S16.** ^13^C NMR spectrum of compound **3a** in DMSO-*d*_6_.


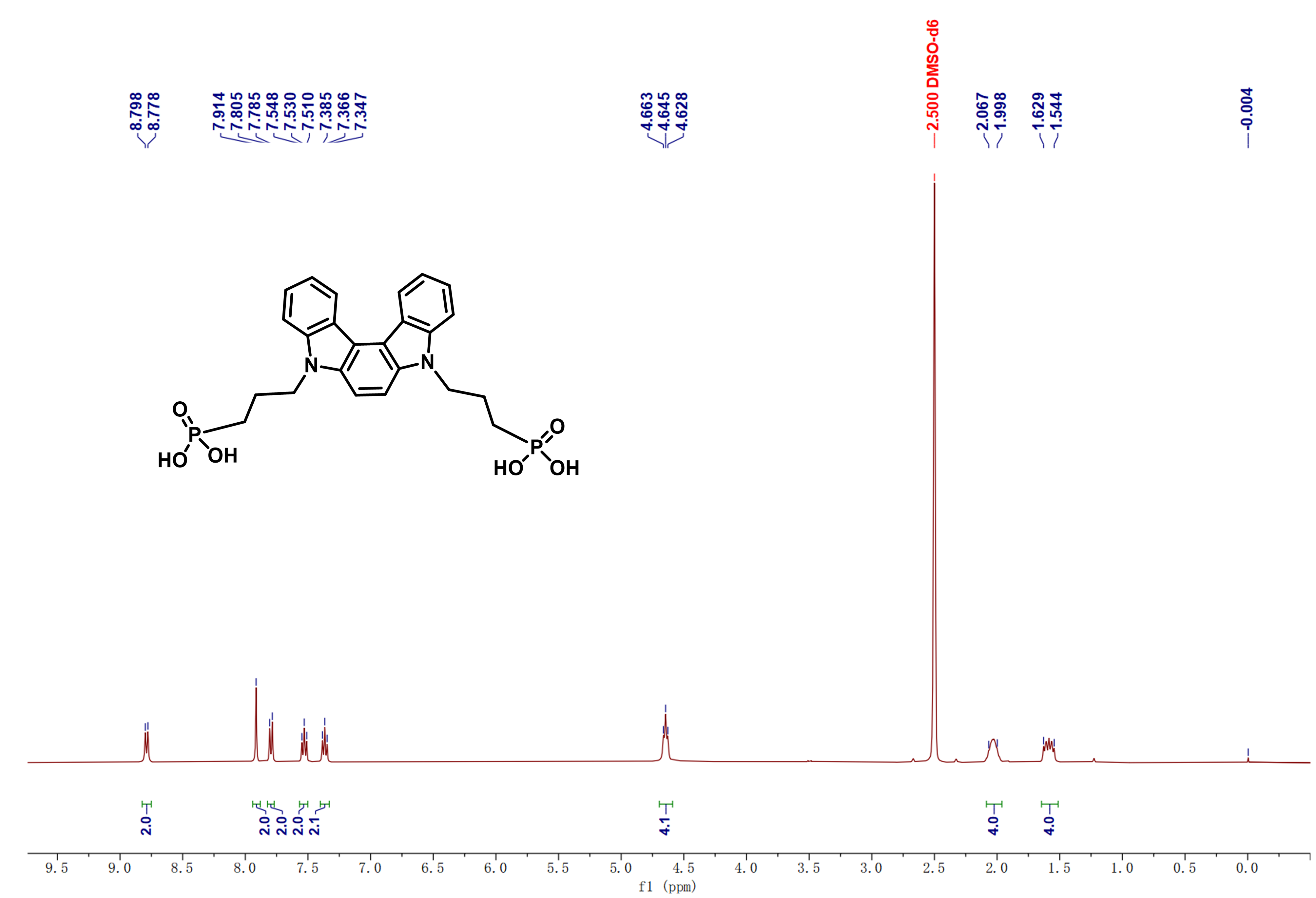


**Figure S17.** ^1^HNMR spectrum of compound **D3PAICz-2** in DMSO-*d*_6_.


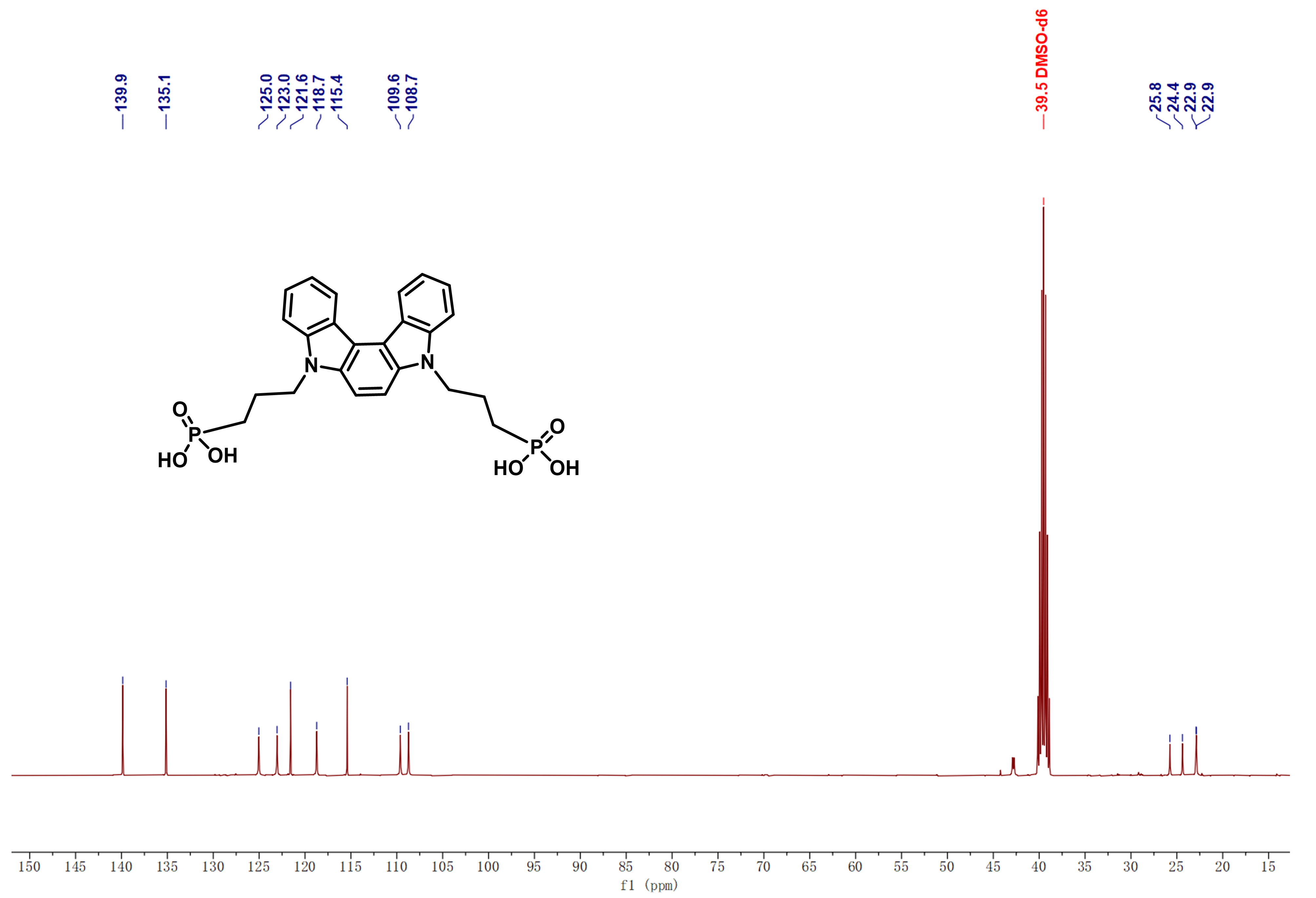


**Figure S18.** ^13^C NMR spectrum of compound **D3PAICz-2** in DMSO-*d*_6_.


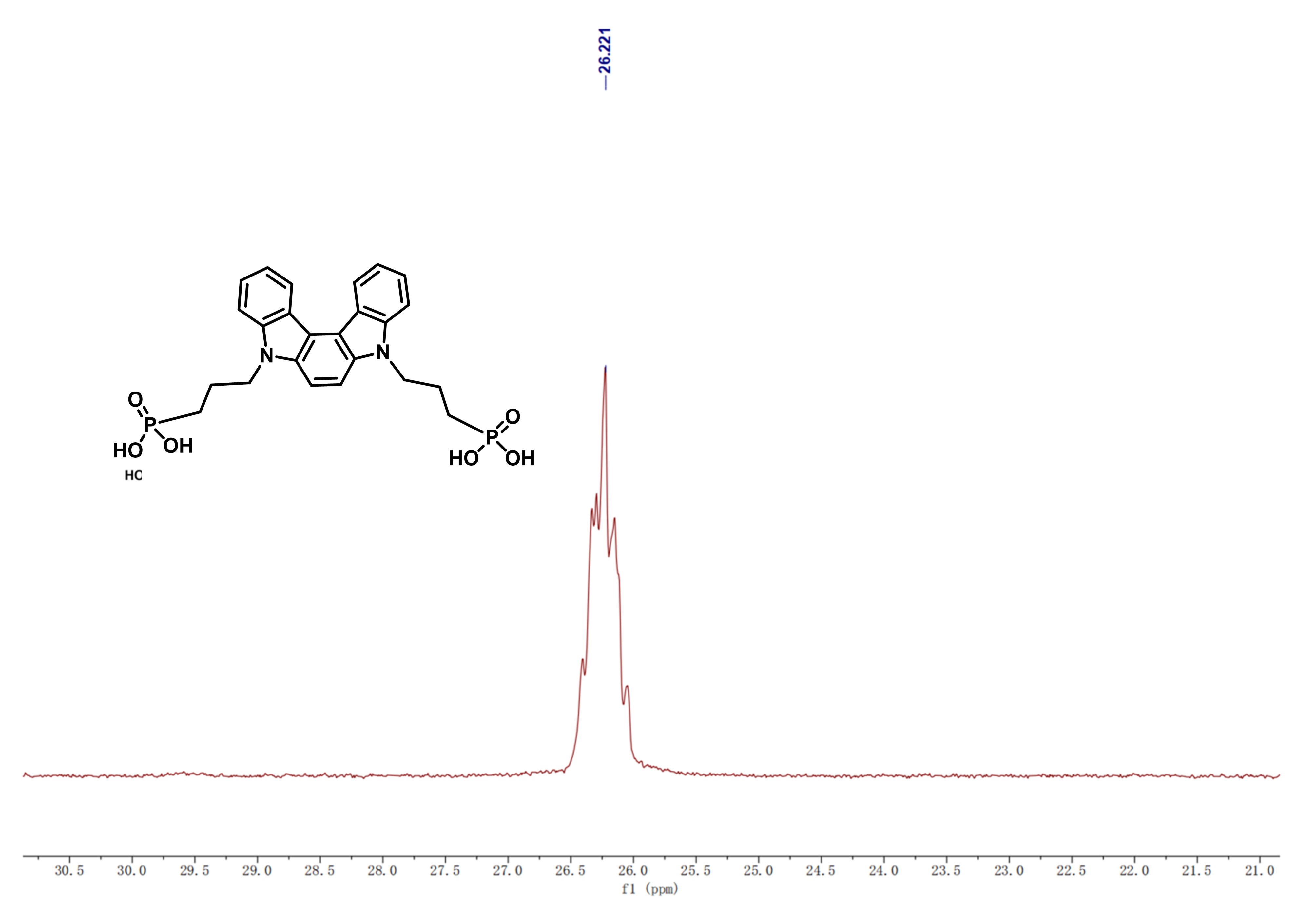


**Figure S19.** ^31^P NMR spectrum of compound **D3PAICz-2** in DMSO-*d*_6_.


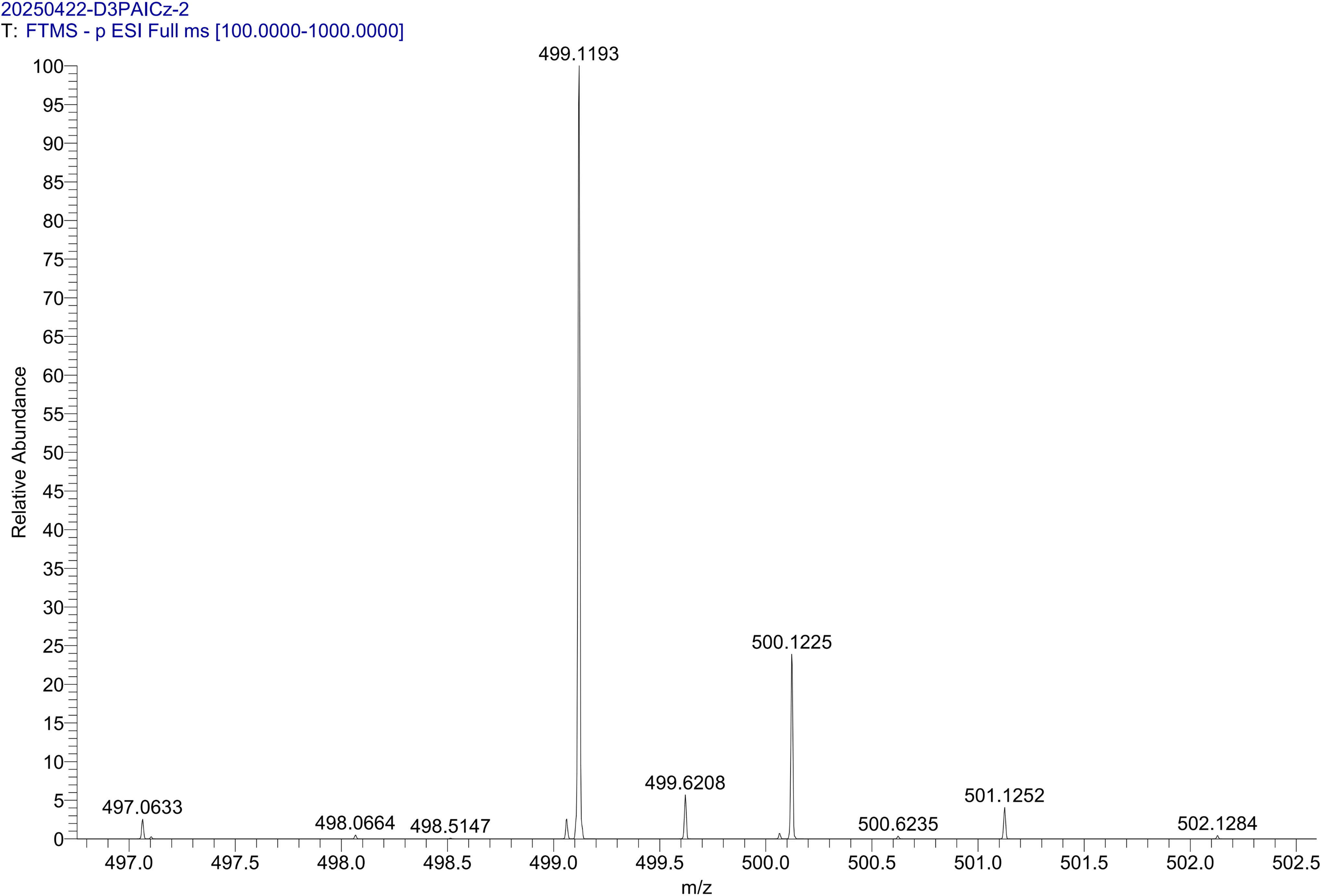


**Figure S20.** HRMS spectrum of **D3PAICz-2**


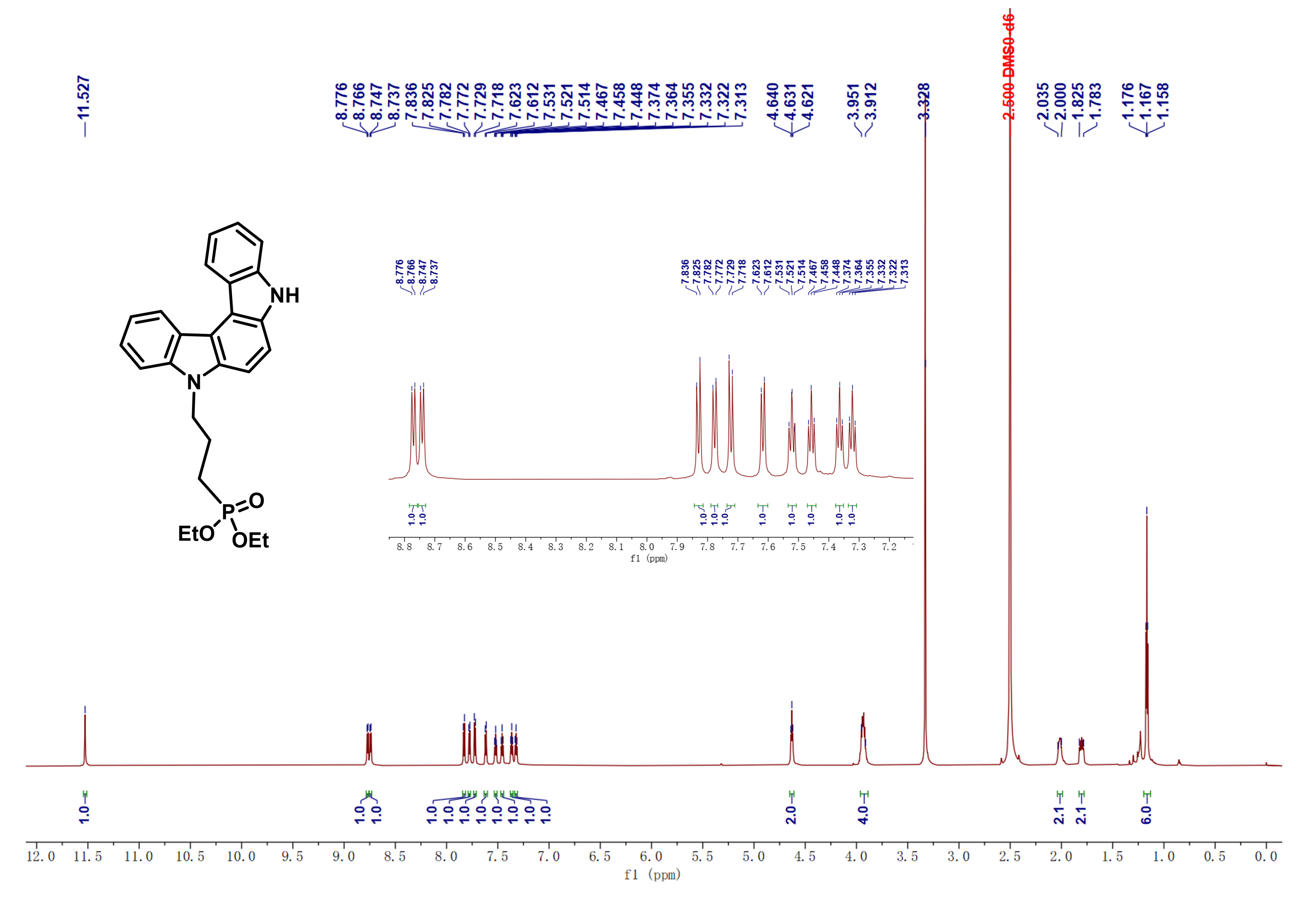


**Figure S21.** ^1^HNMR spectrum of compound **4a** in DMSO-*d*_6_.


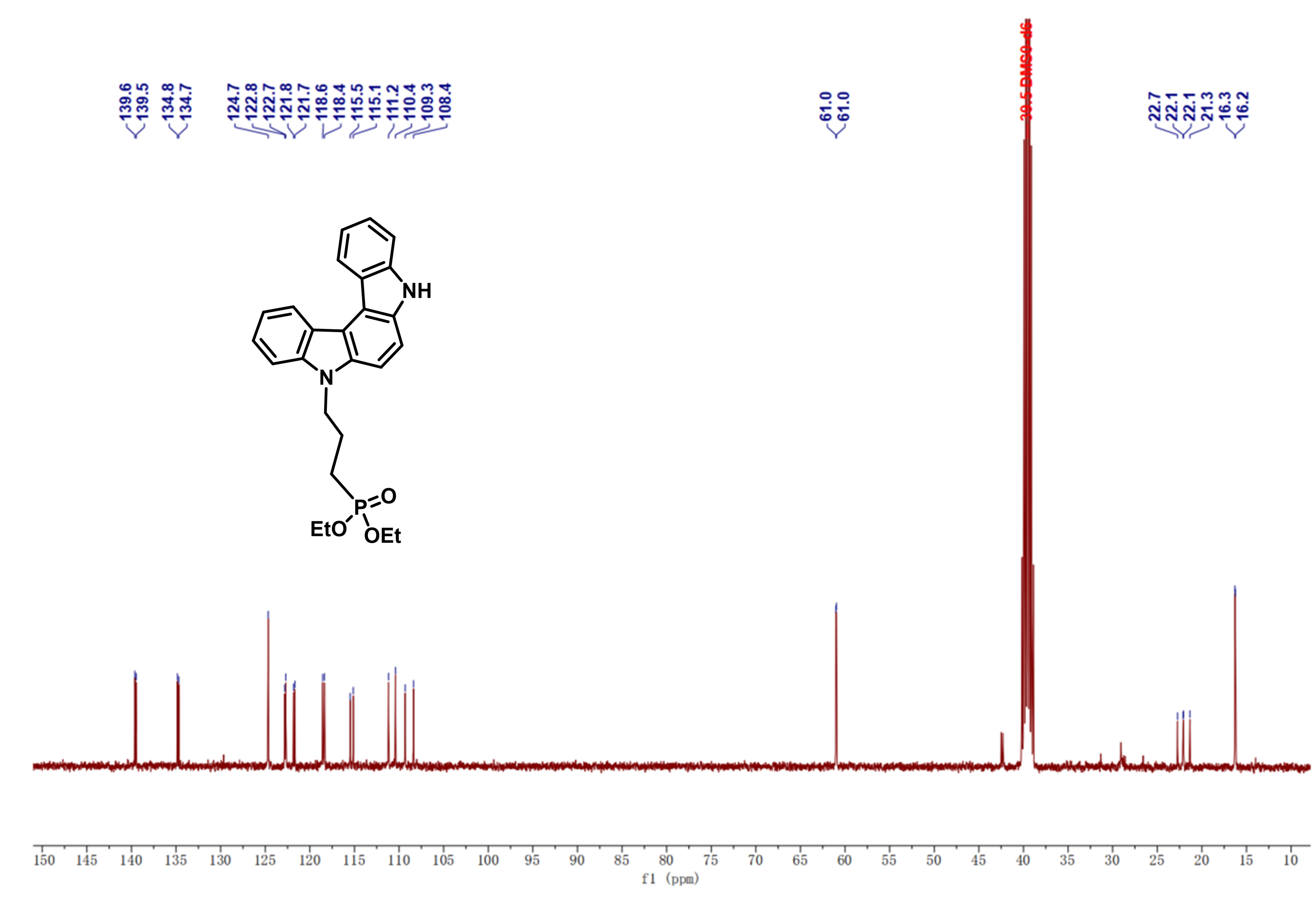


**Figure S22.** ^13^C NMR spectrum of compound **4a** in DMSO-*d*_6_.


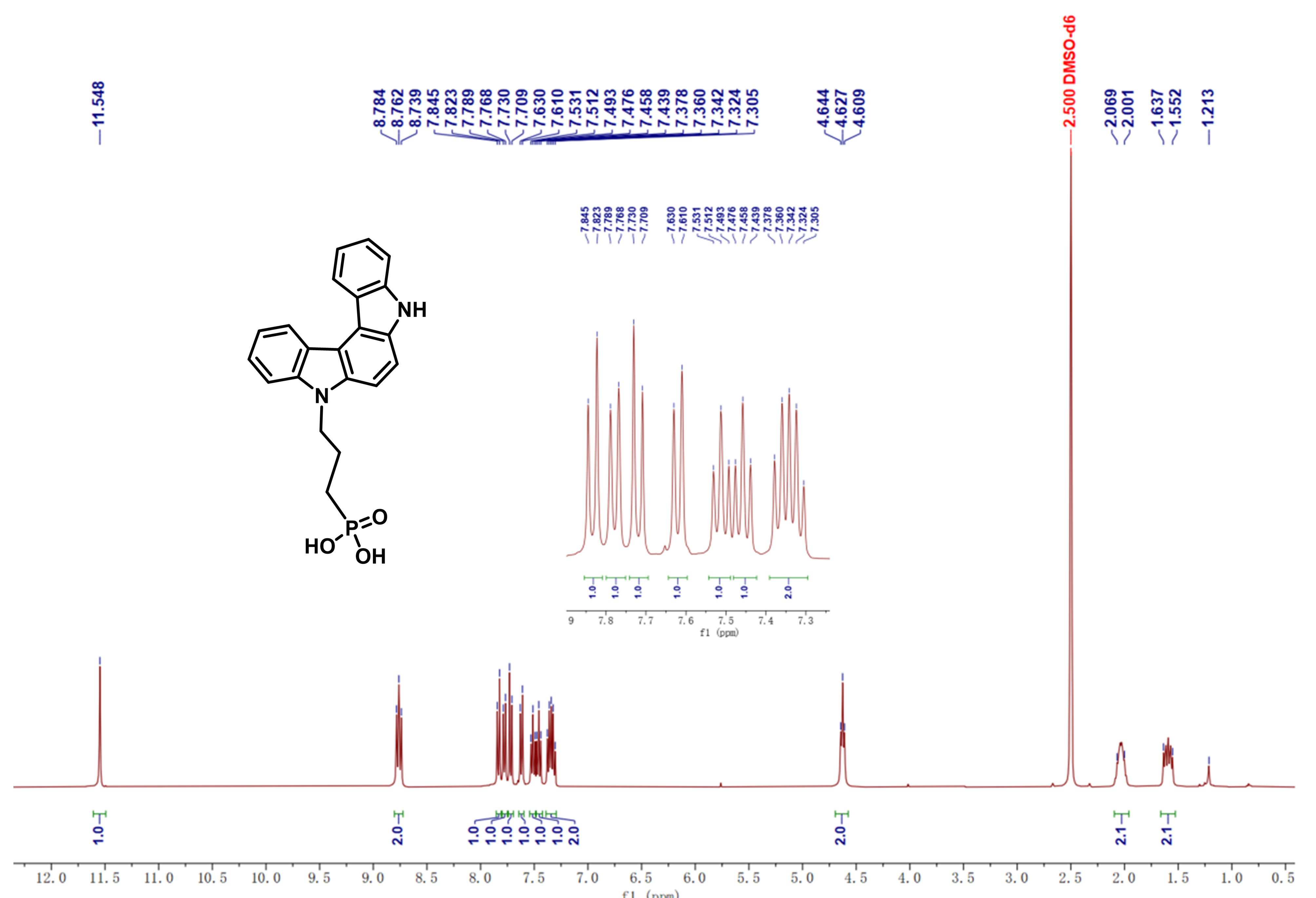


**Figure S23.** ^1^HNMR spectrum of compound **M3PAICz-2** in DMSO-*d*_6_.


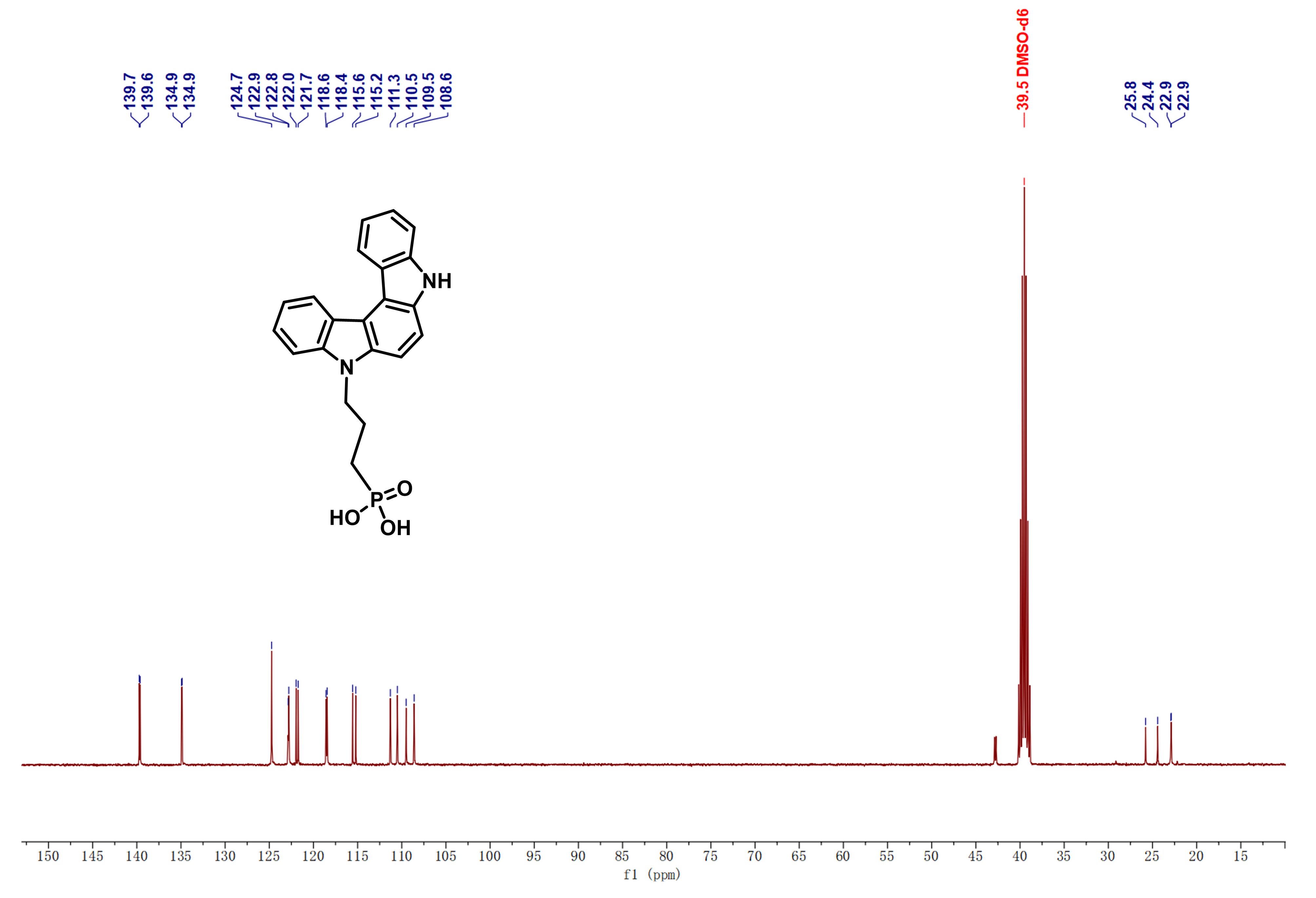


**Figure S24.** ^13^C NMR spectrum of compound **M3PAICz-2** in DMSO-*d*_6_.


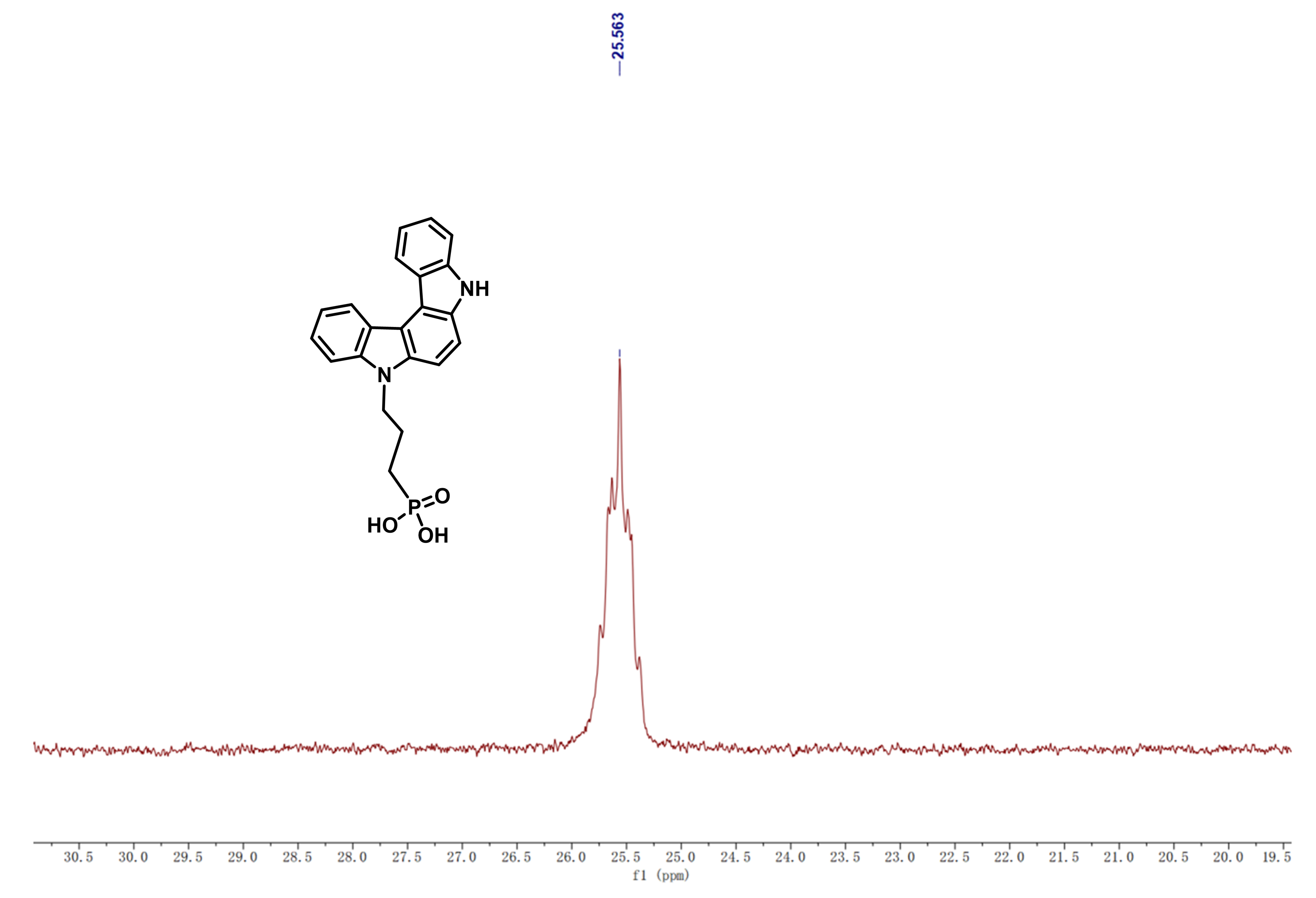


**Figure S25.** ^31^P NMR spectrum of compound **M3PAICz-2** in DMSO-*d*_6_.


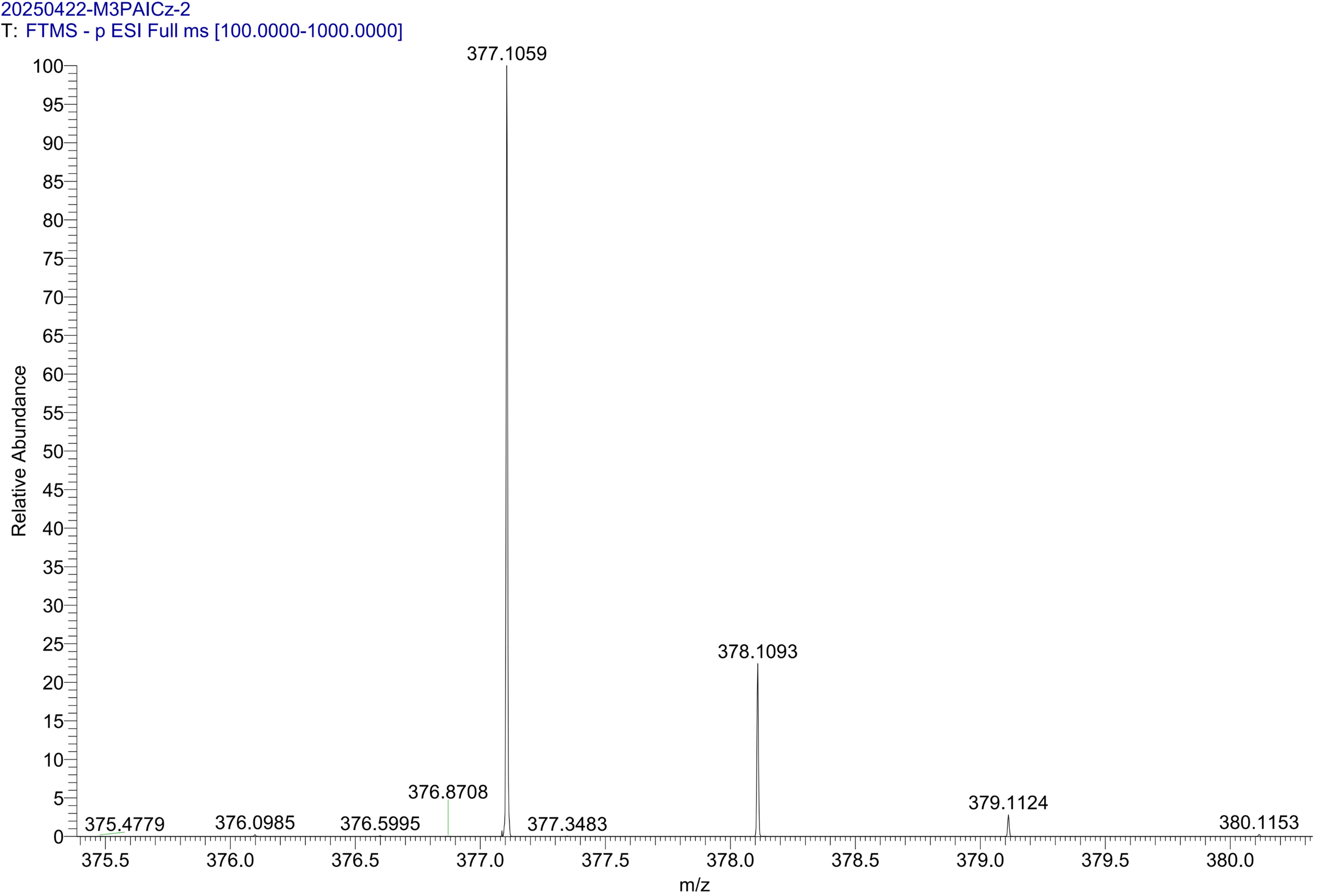


**Figure S26.** HRMS spectrum of **M3PAICz-2**


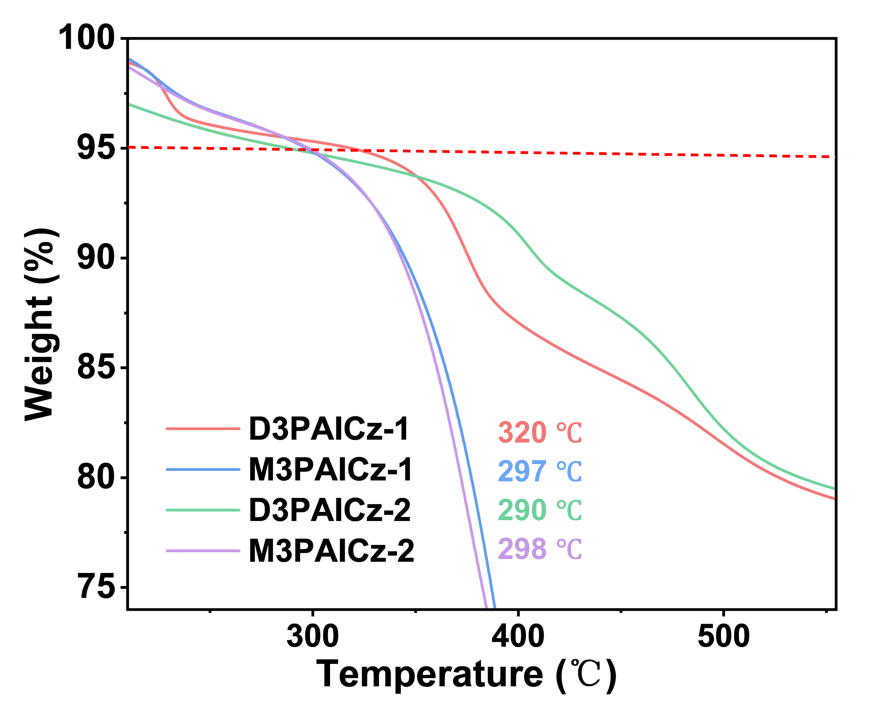


**Figure S27.** TGA curves of different SAMs.


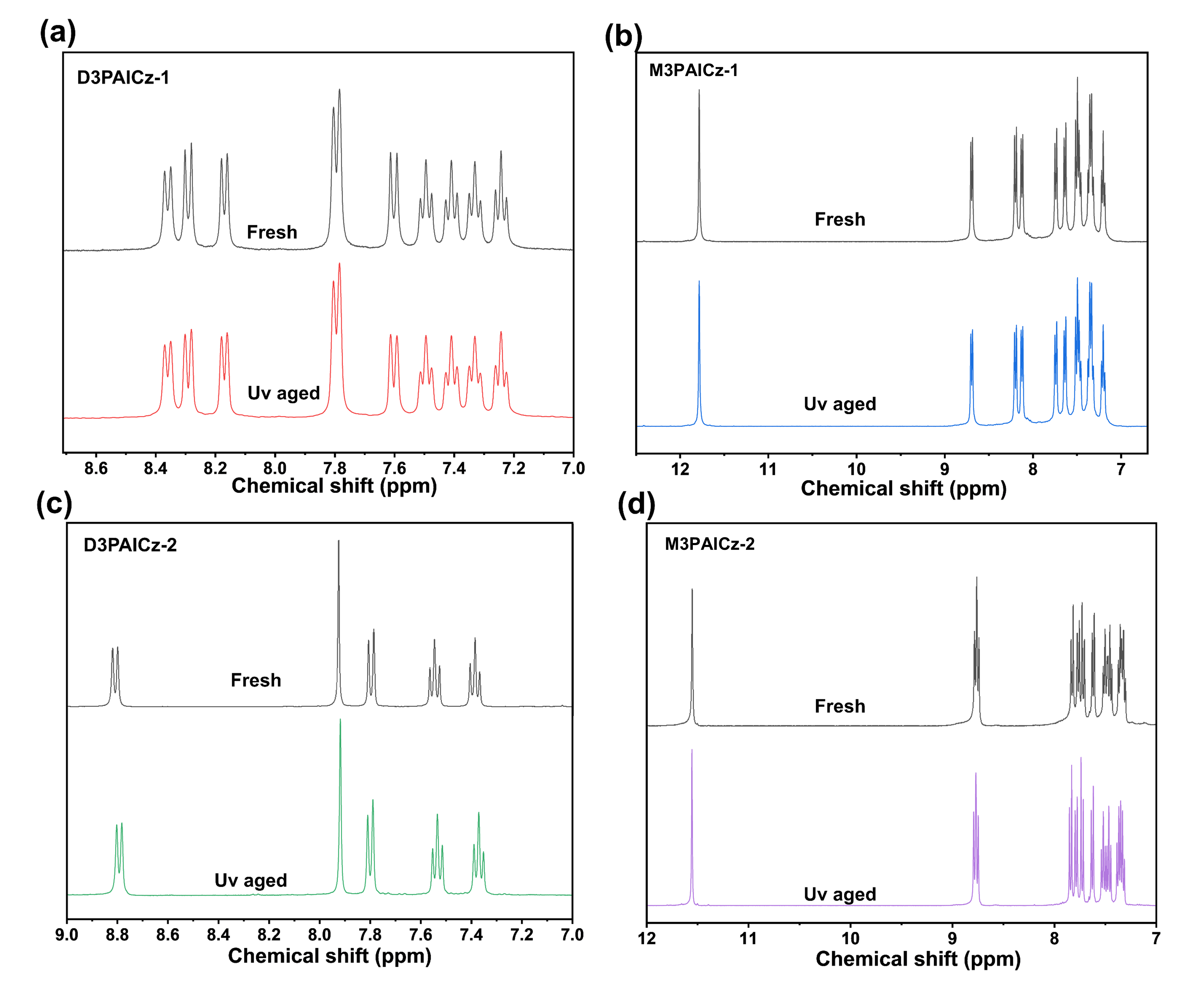


**Figure S28.** ^1^HNMR spectra of (a) D3PAICz-1, (b) M3PAICz-1, (c) D3PAICz-2 and (d) M3PAICz-2 before and after 24 hours of UV irradiation at 365nm.


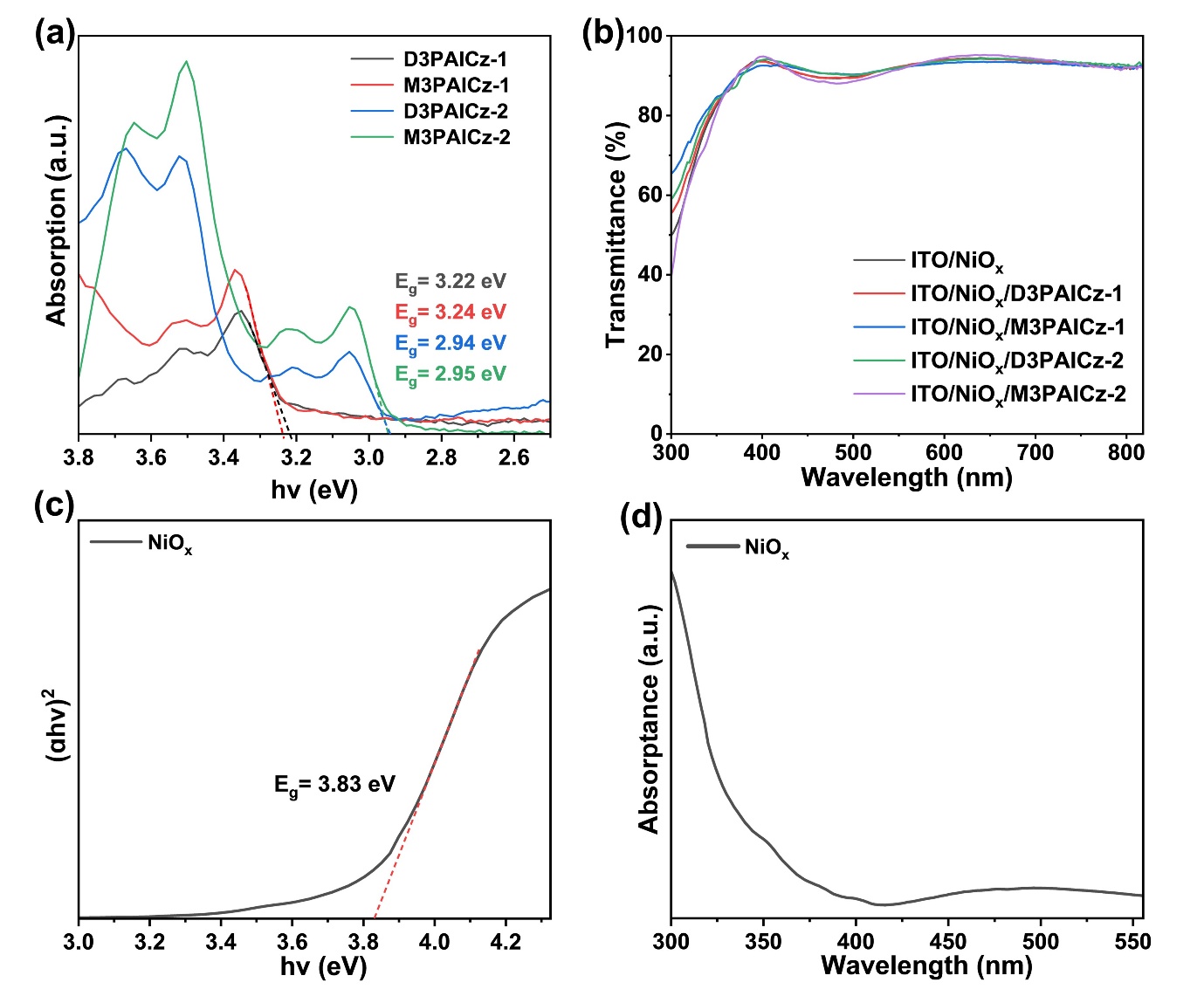


**Figure S29. (**a) UV-vis absorption spectra of the SAMs on NiO_x_. The absorbance of NiO_x_ was subtracted by using NiO_x_ substrate as reference when measuring. (b) UV-vis transmittance spectra of ITO/NiO_x_ and ITO/NiO_x_ with different SAMs on it; (c) The corresponding Tauc plot and (d) UV-vis absorption spectrum of the NiO_x_ film.


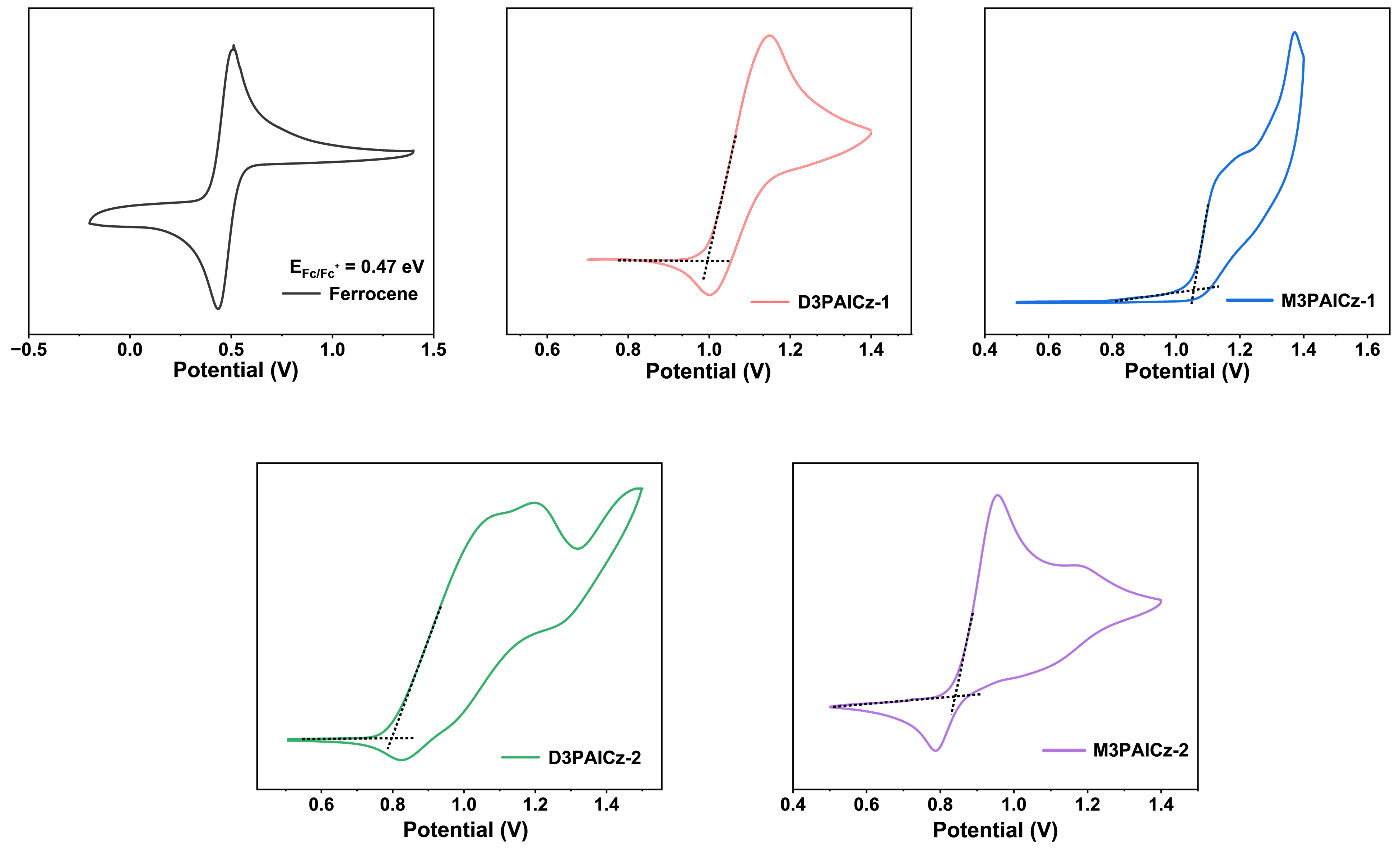


**Figure S30.** Cyclic voltammetry characterized the oxidation potential of SAMs (5×10^-4^ M) in DMF at a scan rate of 0.05V/s with 0.1M NBu_4_ClO_4_ as supporting electrolyte, glassy carbon and platinum wire as working and counter electrodes, and Ag/AgCl as reference electrode. Ferrocene was used as an internal reference.


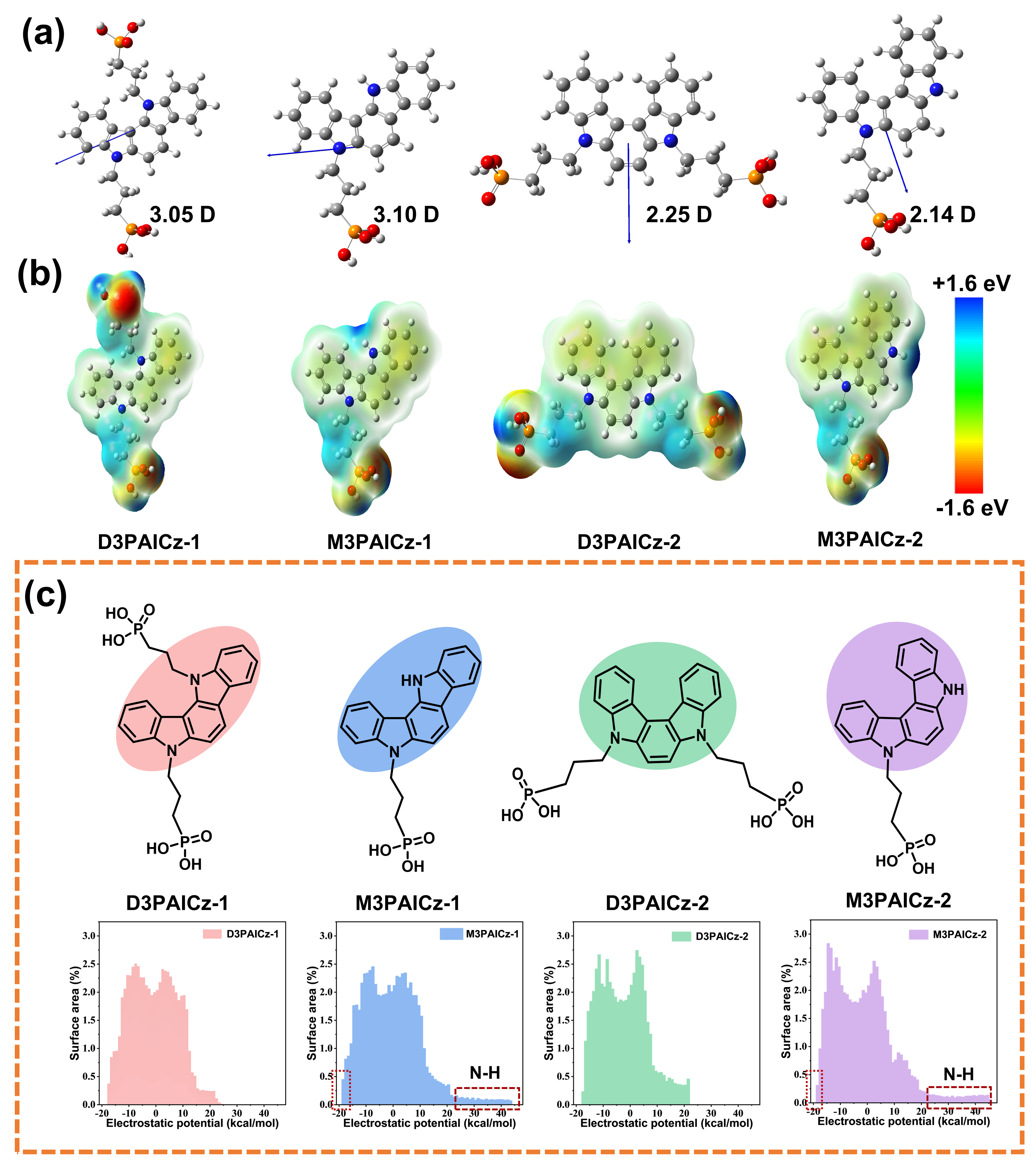


**Figure S31.** Calculation of ESP charge distribution in the conjugated indolocarbazole region.


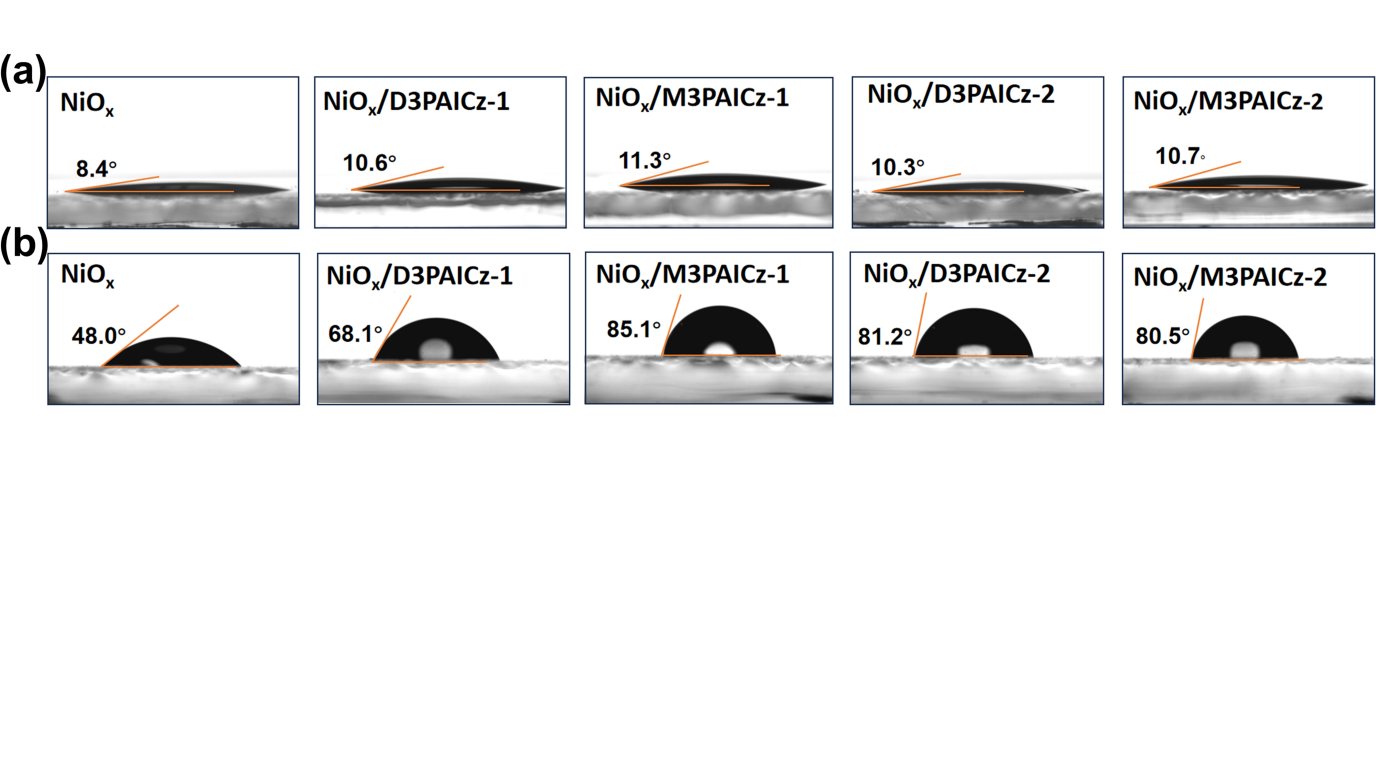


**Figure S32**. Contact angles of a) perovskite precursor solution and (b) water on the NiO_x_ films with and without modifications by different SAMs. The samples were with additional washing.


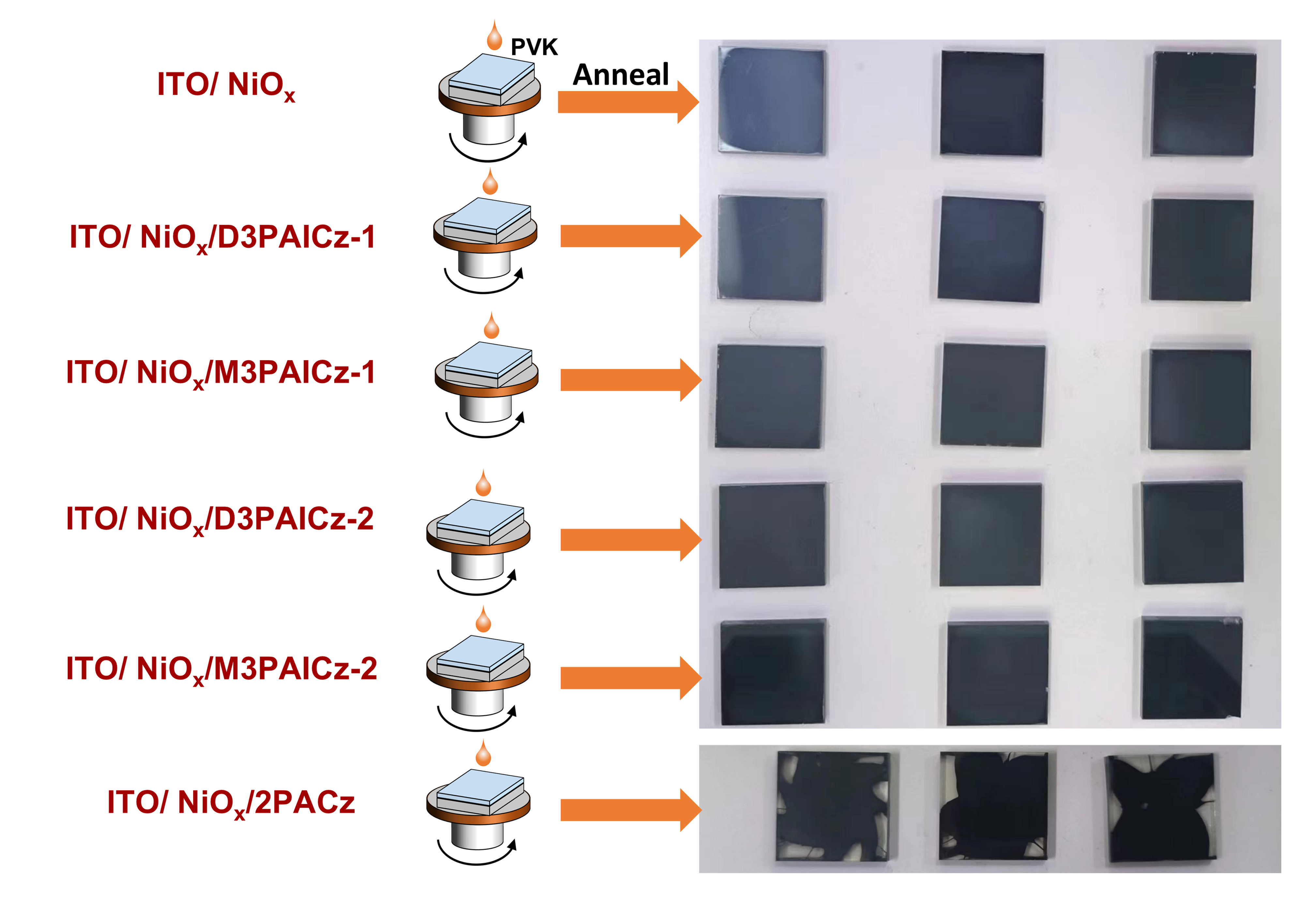


**Figure S33.** Images of perovskite films deposited on larger area (25×25 mm^2^) substrates with different HTLs.


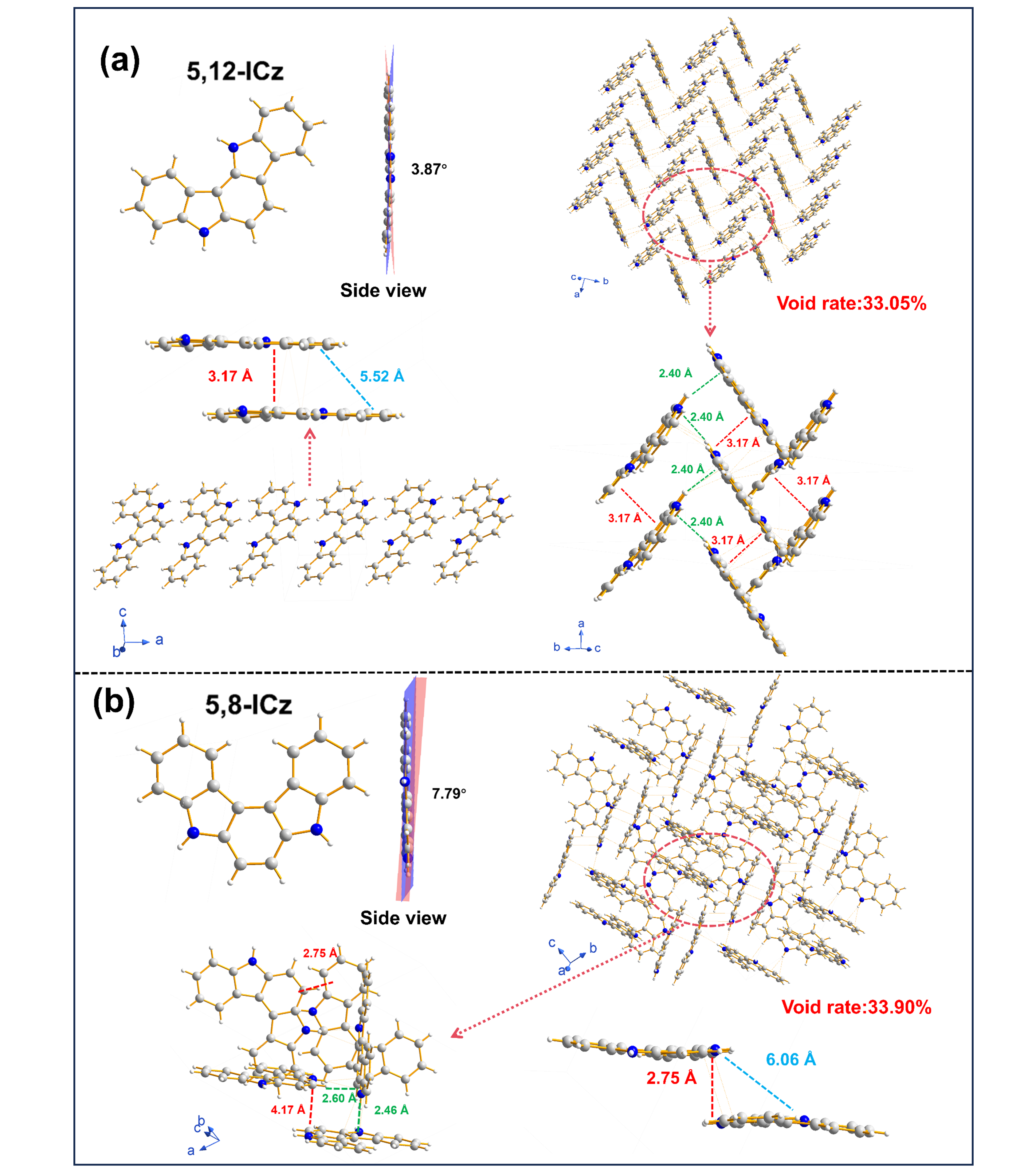


**Figure S34.** Analysis of the Single Crystal Structures of (a) 5,12-ICz and (b) 5,8-ICz
