# Supplementary material for: Defect‐Passivating and Dense Indolocarbazole‐Based Self‐Assembled Monolayers for Efficient Inverted Perovskite Solar Cells With over 26.1% Efficiency
Source: Small. 2026 Jan 19;22(16):e12942. doi: 10.1002/smll.202512942 (PMC12994557; doi:10.1002/smll.202512942)
Supplement: Supplementary file 2 — Supporting file: smll72463‐sup‐0002‐SuppMat.docx [file SMLL-22-e12942-s002.docx]

**Supporting Information**

# Defect-Passivating and dense Indolocarbazole-Based Self-Assembled Monolayers for Efficient Inverted Perovskite Solar Cells with Over 26.1% Efficiency

Xu Fu,^1, 3^ Yuxuan Yang,^1, 4^ Dingqian He,^1,3^ Peng Zhao,^1,3^ Huixin Gao,^1,3^ Zhen Zhu,^2^ Yi Zhang,^1, 4,*^ Bao Zhang^1,3,*^, and Mohammad Khaja Nazeeruddin^2,*^

^1^School of Chemical Engineering and Technology, Tianjin University, Tianjin 300350, P.R. China

^2^School of Integrated Circuits, Southeast University, Wuxi, 214026 Jiangsu, P. R. China

^3^Collaborative Innovation Center of Chemical Science and Engineering, Tianjin University, Tianjin 300072, P.R. China

^4^Institute of Molecular Plus, School of Chemical Engineering and Technology, Tianjin University, Tianjin 300072, P.R. China

E-mail: yi_zhang@tju.edu.cn, baozhang@tju.edu.cn, [mdkhaja.nazeeruddin@epfl.ch](mailto:mdkhaja.nazeeruddin@epfl.ch)

**Table S1.** Single crystal data for these indolocarbazoles.

| Compounds | **5,12-ICZ** | **5,8-ICZ** |
| --- | --- | --- |
| Empirical formula | C_18_H_12_N_2_ | C_18_H_12_N_2_ |
| Molecular weight | 256.30 | 256.30 |
| Temperature (K) | 298.15 | 298.15 |
| Wavelength (Å) | 0.71073 | 0.71073 |
| Crystal system | orthorhombic | triclinic |
| Space group | P2ac2ab | -P1 |
| a (Å) | 5.6425(14) | 11.4804(18) |
| b (Å) | 9.539(3) | 12.291(2) |
| c (Å) | 23.486(7) | 15.196(3) |
| α (°) | 90 | 76.736(6) |
| β (°) | 90 | 69.918(5) |
| γ (°) | 90 | 74.909(6) |
| Volume (Å^3^) | 1264.2(6) | 1921.0(6) |
| Z | 4 | 6 |
| D_calc_(g cm^-3^) | 1.347 | 1.329 |
| F(000) | 536.0 | 804.0 |
| Void rate | 33.05% | 33.90% |
| CCDC No. | 2077291 | 2077290 |


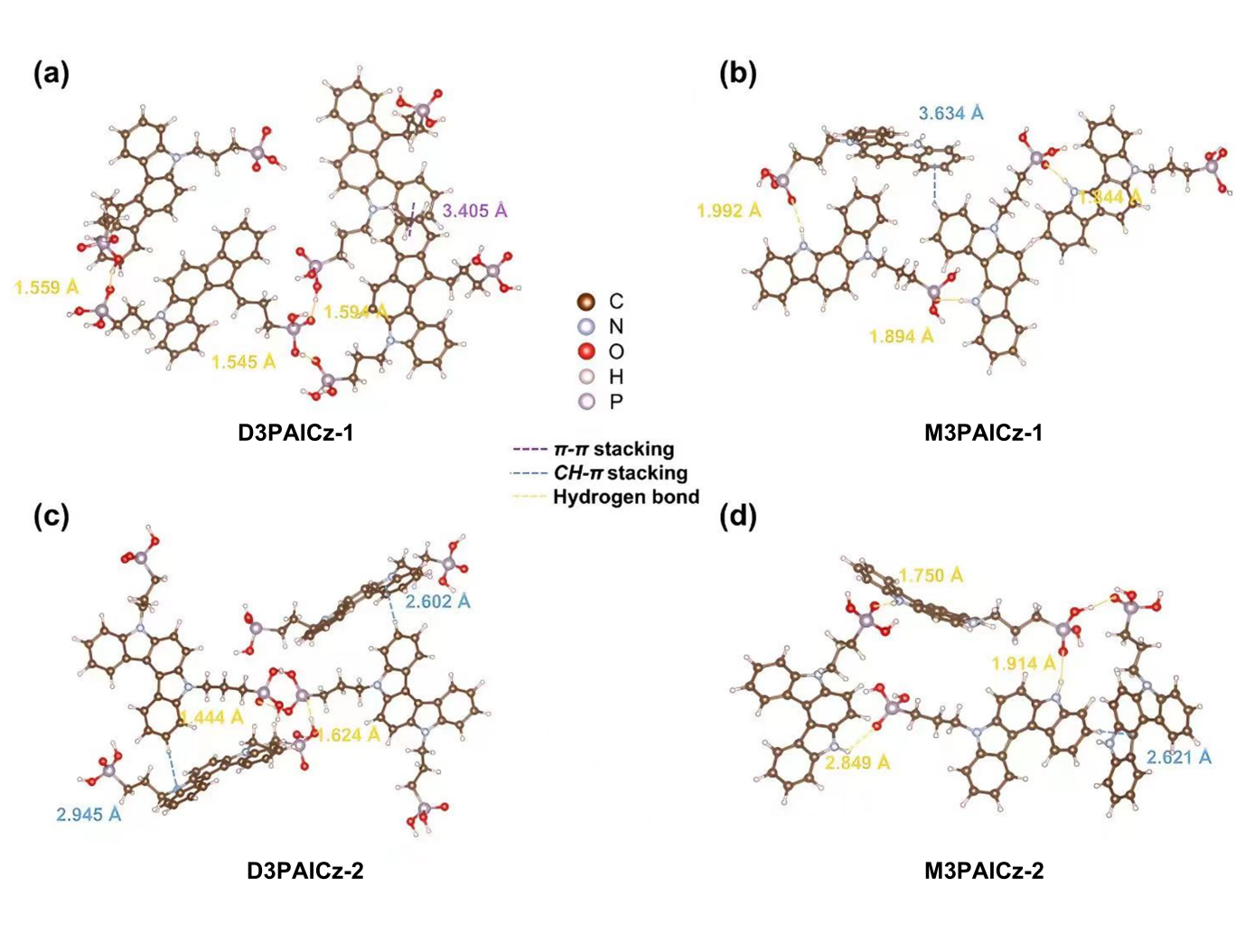


**Figure S35.** Simulated intermolecular stacking after structure optimization using DFT calculations for (a) D3PAICz-1, (b) M3PAICz-1, (c) D3PAICz-2 and (d) M3PAICz-2. Different colored numbers indicate specific interaction distances: yellow represents hydrogen bonding interactions, blue denotes CH-π interactions, and purple corresponds to π-π interactions


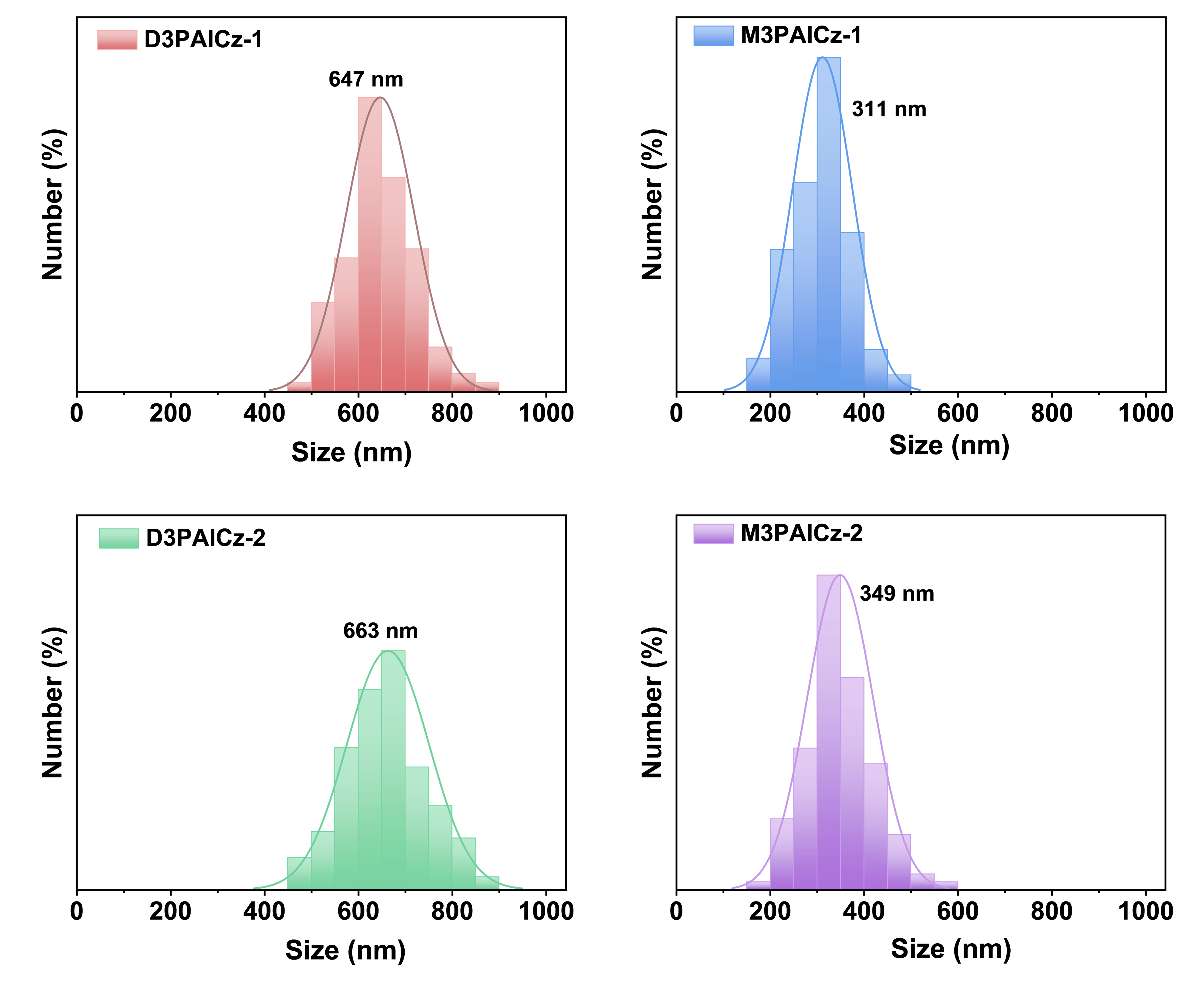


**Figure S36.** DLS analysis for different SAMs in ethanol.


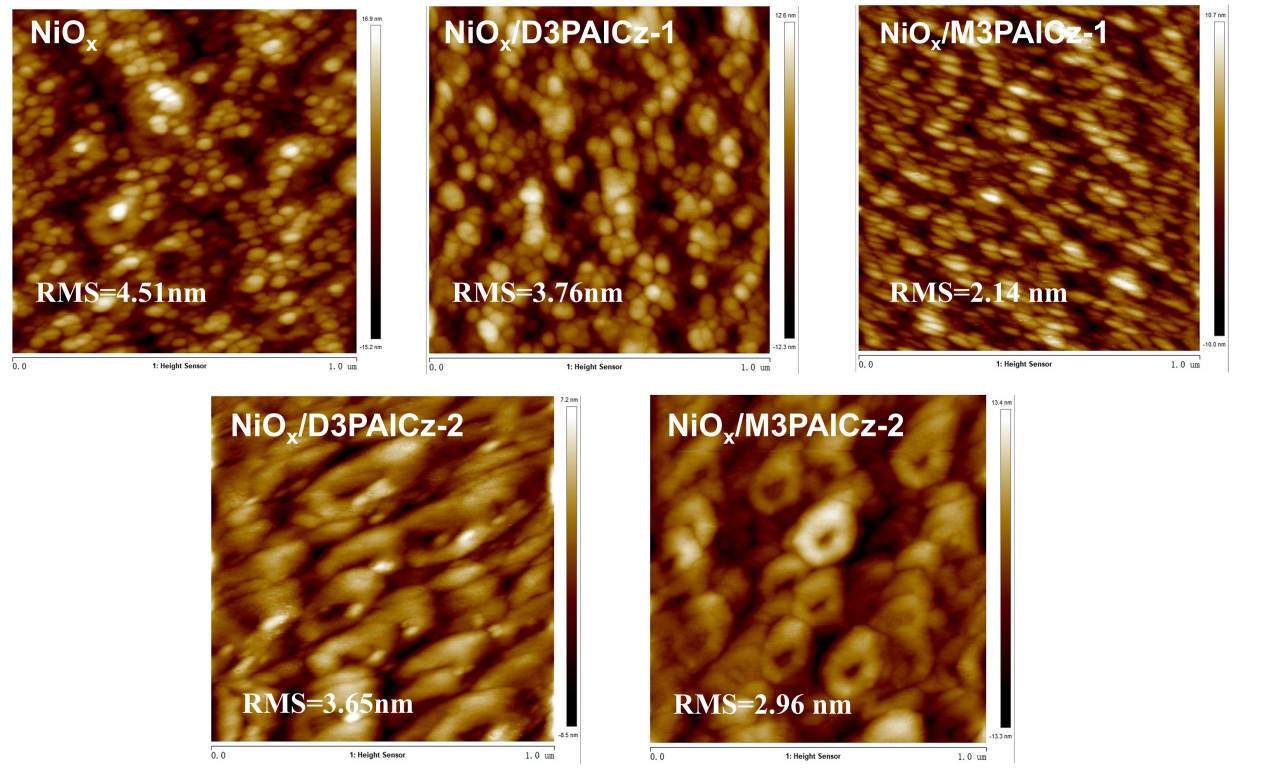


**Figure S37.** AFM images NiOx and NiOx/SAMs substrate.


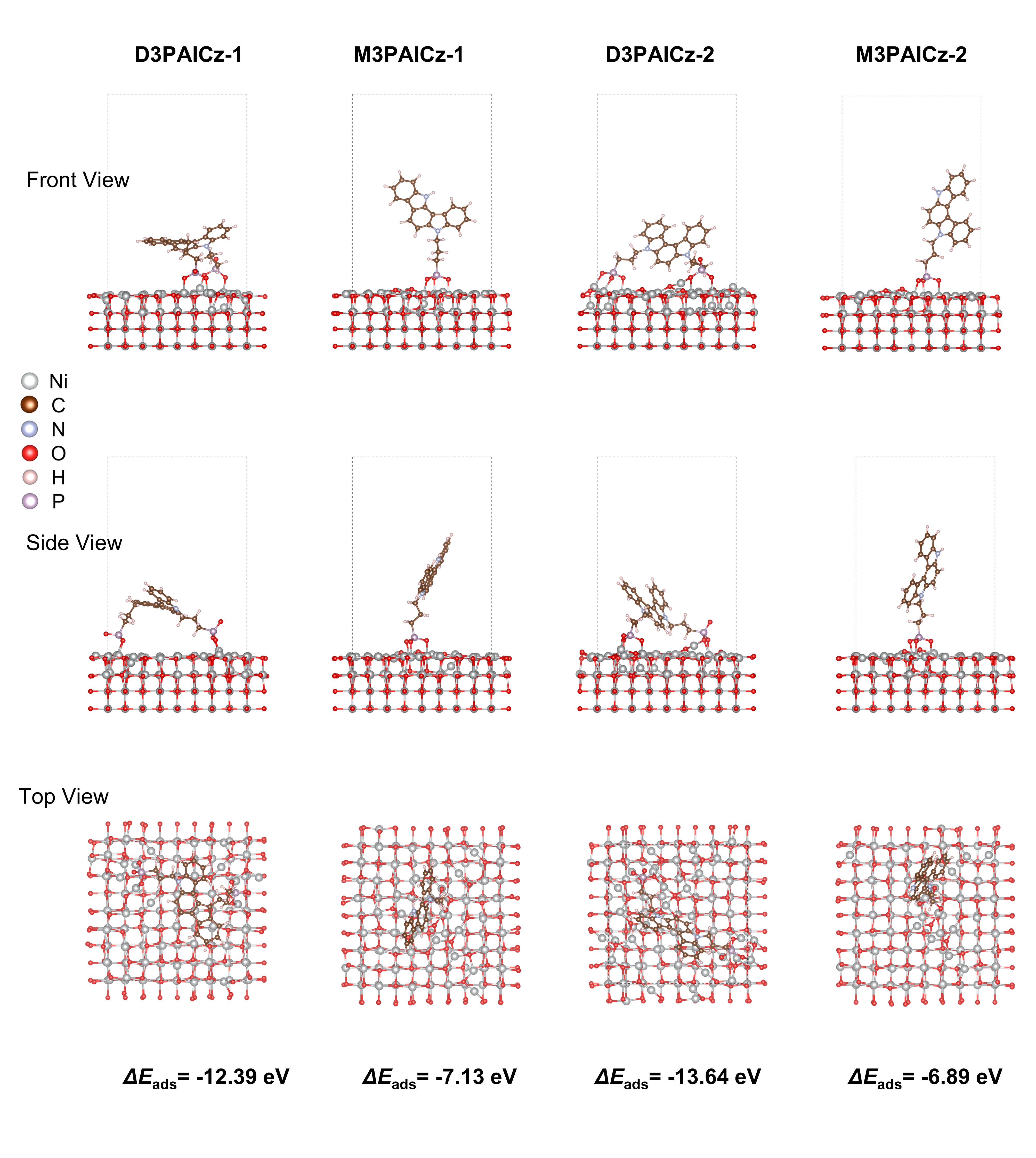


**Figure S38**. DFT-calculated binding configurations of four SAM molecules with NiOx (after structural optimization)

**
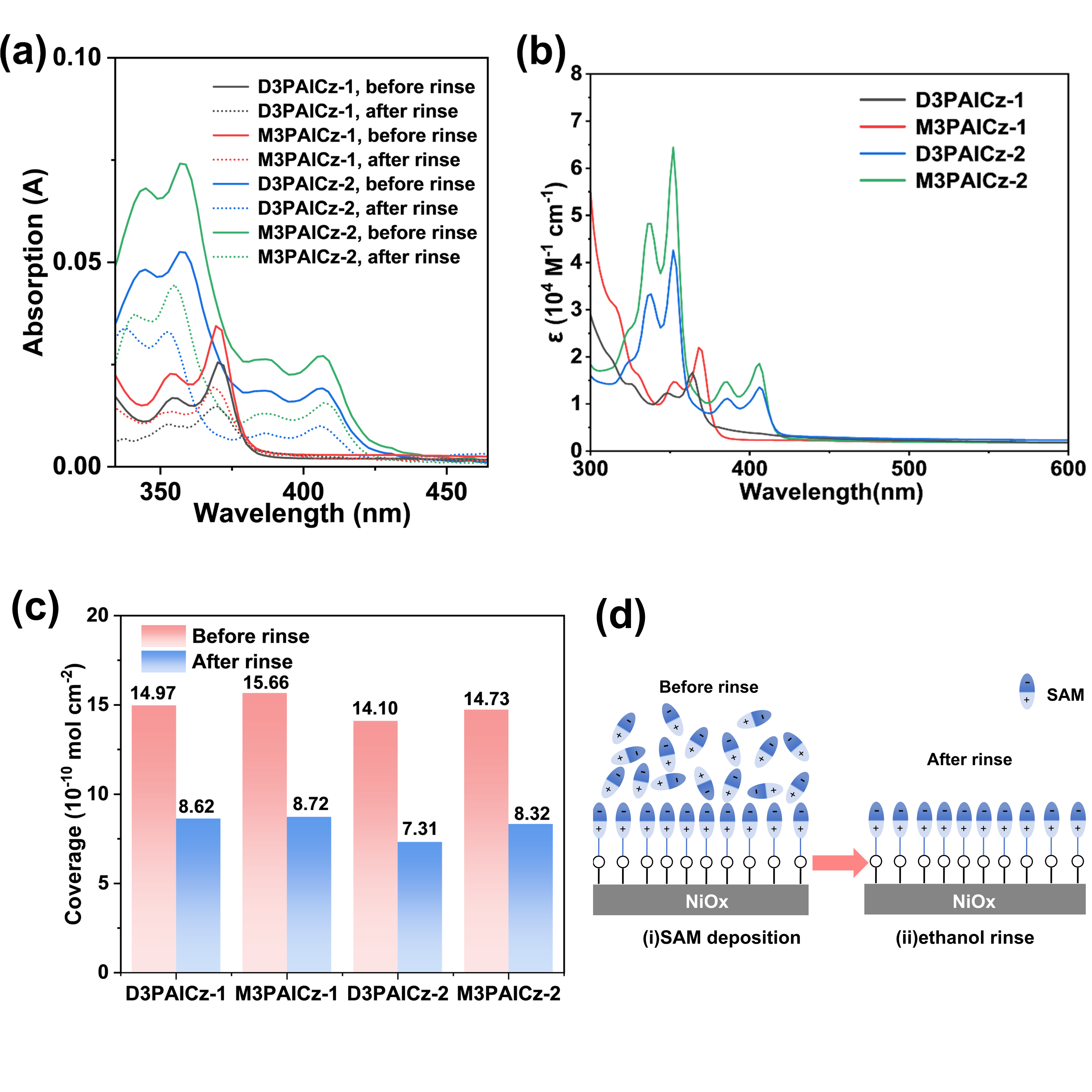
**

**Figure S39.** (a) UV-visible absorption spectra of the SAMs on NiO_x_ before and after rinsing with ethanol. The absorbance of NiO_x_ was subtracted by using NiO_x_ substrate as reference when measuring. (b) UV-visible absorption spectra of the SAM molecules in dimethylformamide (10^-5^ M). (c) The surface coverage of different SAMs on NiOx before and after rinsing with ethanol. (d) Schematic depiction of deposition and desorption of SAM on NiO_x_ surface.

**Table S2.** Calculation of surface coverage.

| **SAMs** | ***A*(*λ*)**  **[10^-2^]** | | ***ε*(*λ*)**  **[10^4^ M^-1^ cm^-1^]** | ***Г*^(a)^**  **[10^–10^ mol cm^–2^]** | |
| --- | --- | --- | --- | --- | --- |
|  | **Before rinse** | **After rinse** |  | **Before rinse** | **After rinse** |
| D3PAICz-1 | 2.55 | 1.44 | 1.67 | 14.97 | 8.62 |
| M3PAICz-1 | 3.36 | 1.91 | 2.19 | 15.66 | 8.72 |
| D3PAICz-2 | 1.89 | 0.98 | 1.34 | 14.10 | 7.31 |
| M3PAICz-2 | 1.53 | 1.53 | 1.84 | 14.73 | 8.42 |

1. *Г =* (*A*(*λ*)/*ε(λ)*/1000)*,* where *Г* is the surface coverage (mol cm^-2^), *A*(*λ*) is the absorbance of SAM at the absorption maximum and ε(λ) is the molar extinction coefficient of SAM molecules.

**Note S1.** Estimating the surface density of SAM molecules adsorbed on NiO_x_ using Cyclic Voltammetry.

The number of SAM molecules adsorbed on NiO_x_ surface can be estimated from cyclic voltammetry by observing the dependency of the oxidative peak intensity on the scan rate as follows:


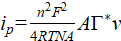


In the above equation, *i_p_* (A) is the oxidative peak current, υ (V s^–1^) is the voltage scan rate, n is the number of electrons transferred, F (96,485.33 C mol^–1^) is the Faraday constant, R (8.3144 J K^–1^ mol^–1^) is the universal gas constant, T (K) is the temperature, NA (6.022 × 1023 mol^–^1) is the Avogadro constant, A (cm^2^) is the electrode surface area, and Γ* (molecules cm^–2^) is the surface density. The number of the adsorbed molecules per unit area can be determined experimentally by measuring the slope of *i_p_* vs υ. Using ITO/NiOx/SAM substrates as working electrodes, CV measurements were performed at different scan rates in 1,2-dichlorobenzene (o-DCB) solutions with 0.1 M tetrabutylammonium hexafluorophosphate (^n^Bu_4_N^+^PF_6_^–^) as a supporting electrolyte. Finally, the surface density of D3PAICz-1, M3PAICz-1, 3PAICz-2 and M3PAICz-1 was determined to be 2.92 × 10^13^, 3.15 × 10^13^, 2.78 × 10^13^, and 2.86 × 10^13^ molecules cm^–2^, respectively.


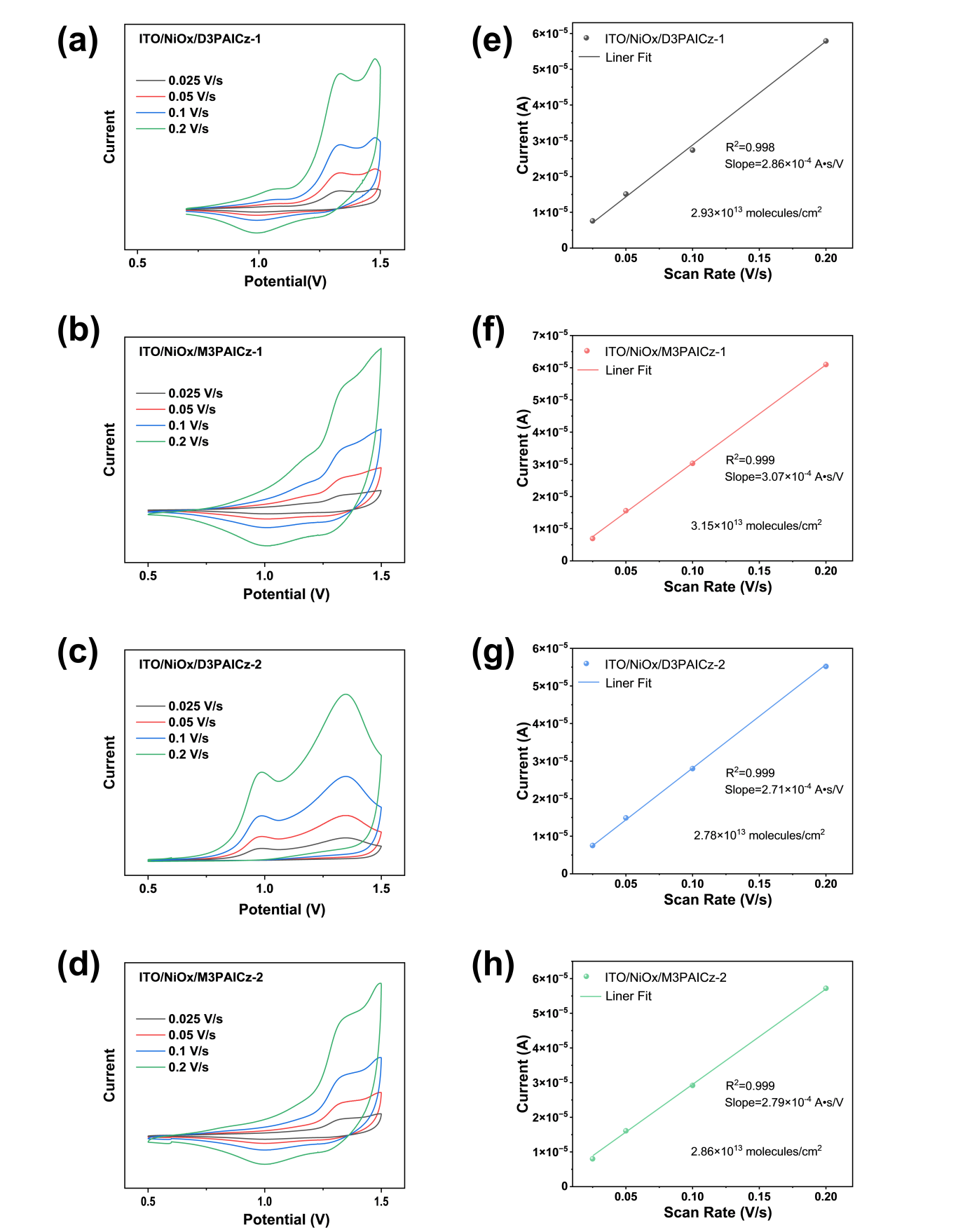


**Figure S40.** Cyclic voltammograms of the four SAMs adsorbed on NiOx substrates measured in o-DCB solution under different scan rate (a–d) and their corresponding peak current vs scan rate chart (e–h)


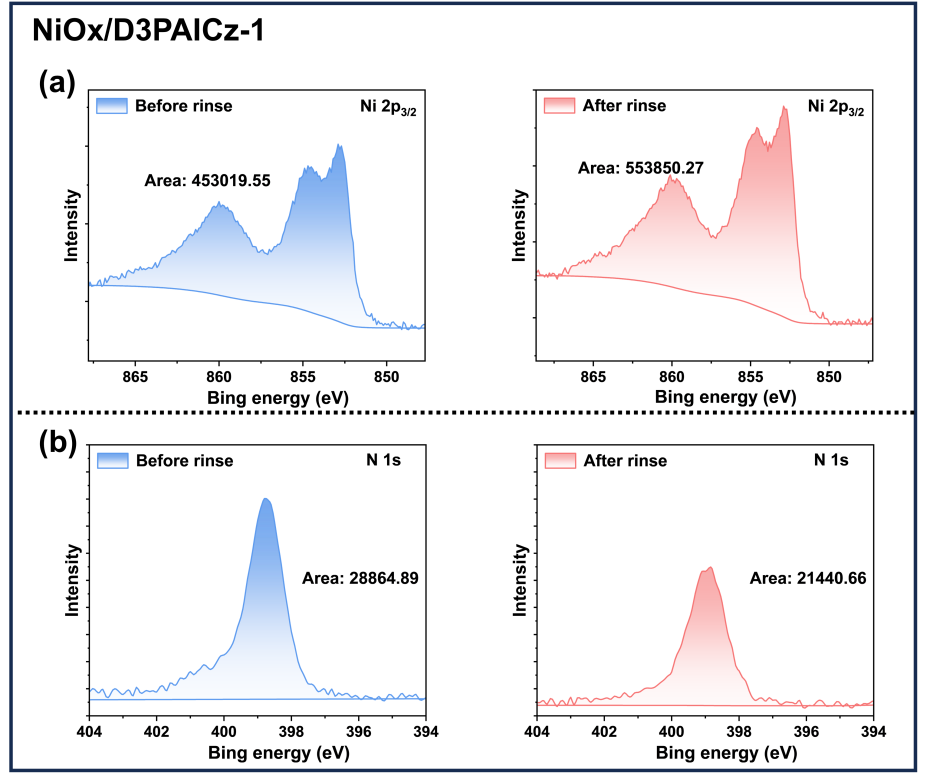


**Figure S41.** X-ray photoelectron spectra (XPS) of D3PAICz-1 on NiOx substrate before and after ethanol rinsing (a) Ni 2p_3/2_ and (b) N 1s.


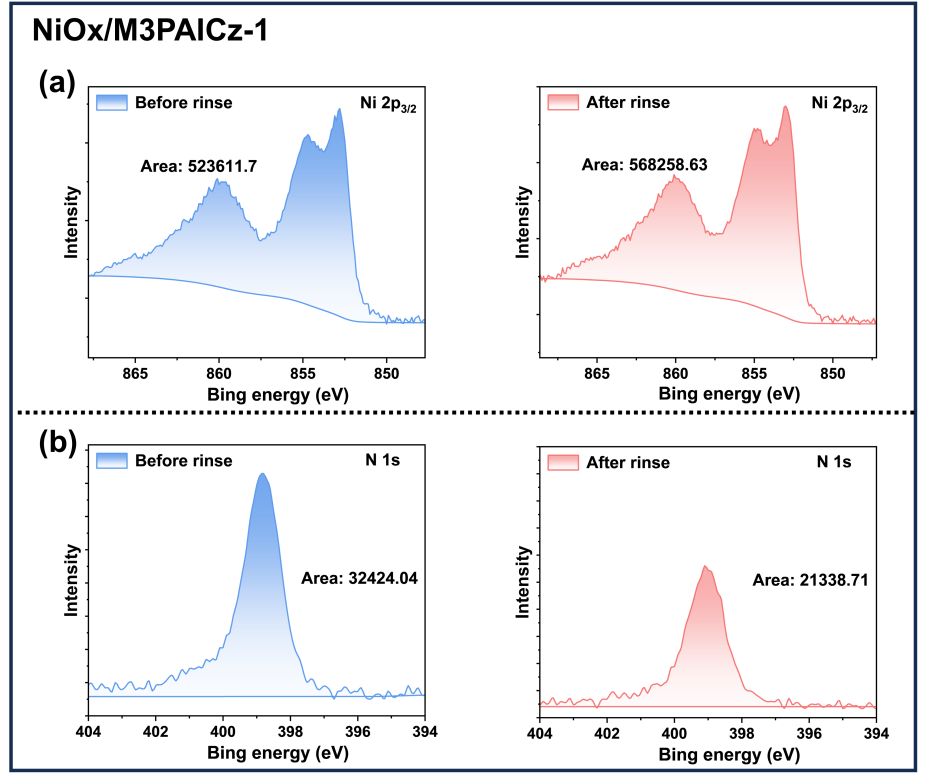


**Figure S42.** X-ray photoelectron spectra (XPS) of M3PAICz-1 on NiOx substrate before and after ethanol rinsing (a) Ni 2p3/2 and (b) N 1s.


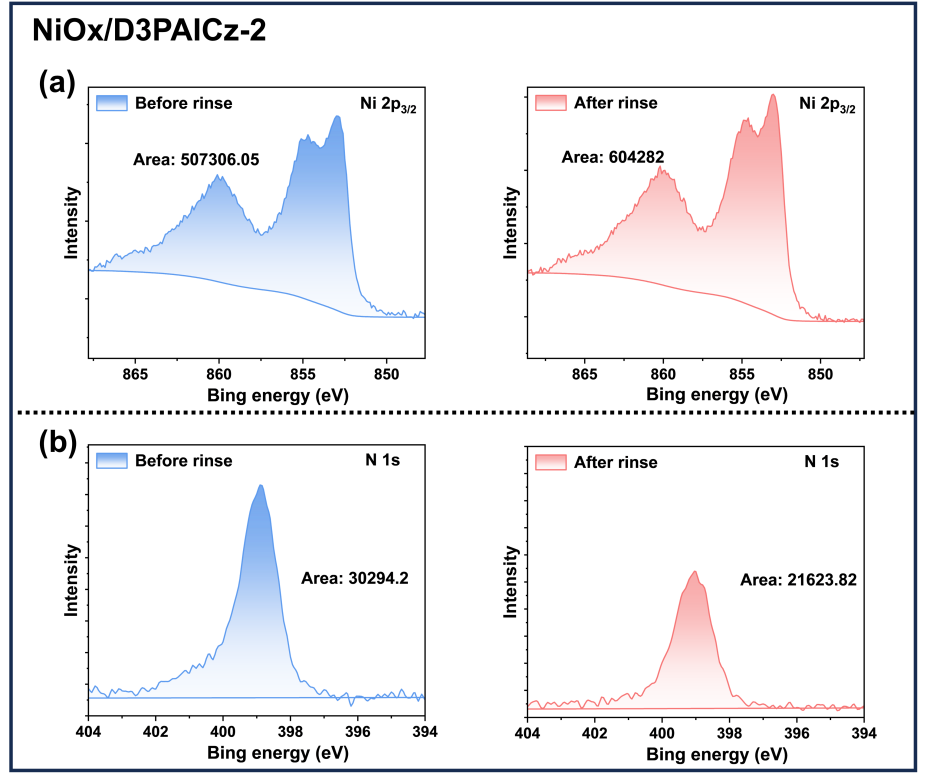


**Figure S43.** X-ray photoelectron spectra (XPS) of D3PAICz-2 on NiOx substrate before and after ethanol rinsing (a) Ni 2p3/2 and (b) N 1s.


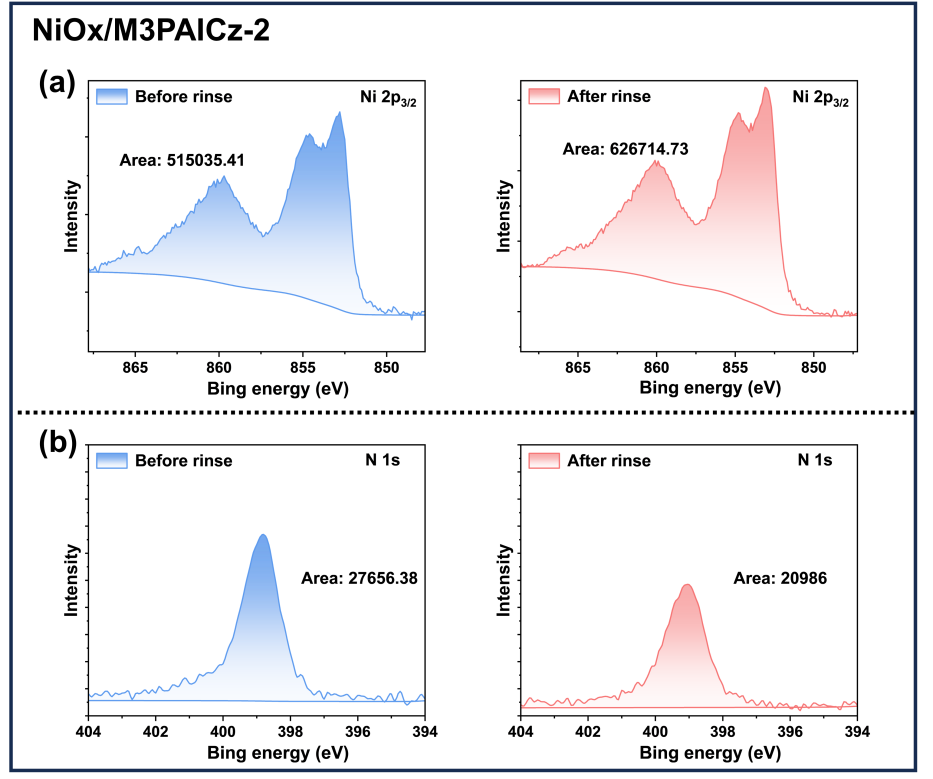


**Figure S44.** X-ray photoelectron spectra (XPS) of M3PAICz-2 on NiOx substrate before and after ethanol rinsing (a) Ni 2p3/2 and (b) N 1s.


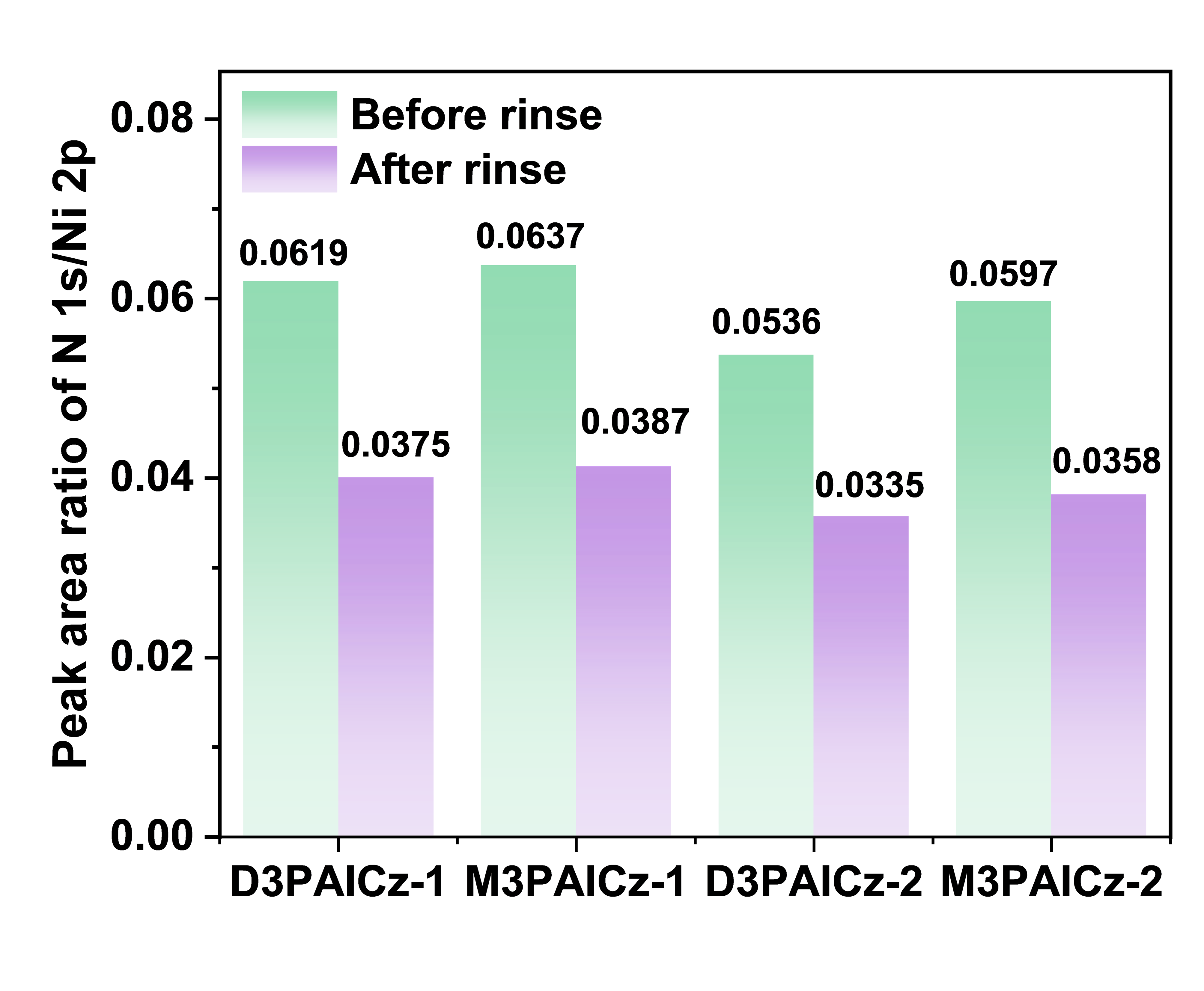


**Figure S45.** Peak area ratio (XPS) of N/Ni for NiO_x_/SAMs before and after rinsing ing with ethanol.


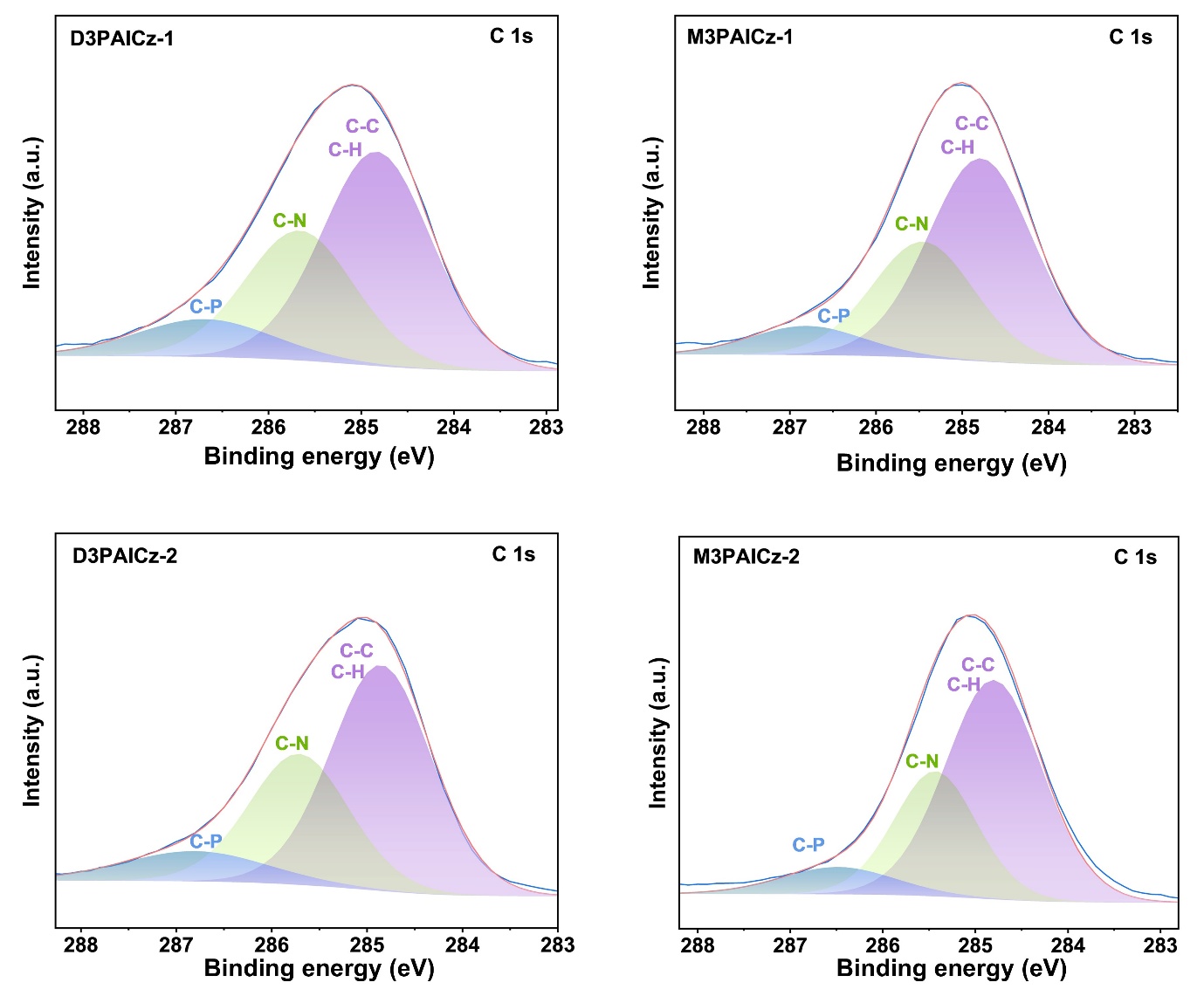


**Figure S46.** XPS spectra of C 1s spectra of ITO/NiO_x_ substrates modified with different SAMs.


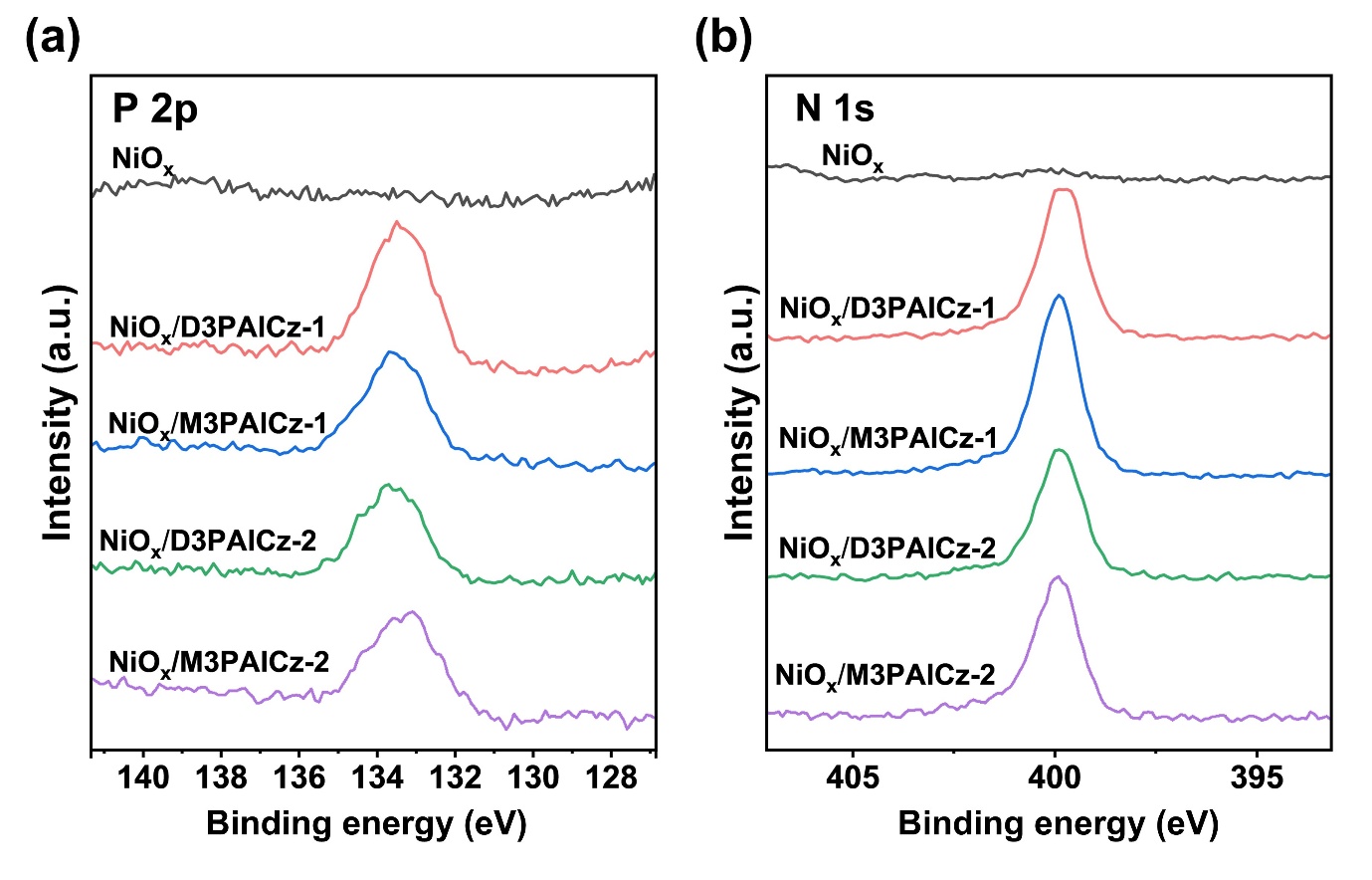


**Figure S47.** XPS spectra of (a) P 2p and (b) N 1s spectra of bare NiO_x_ and SAM-modified NiO_x_ substrates.

**Table S3.** Summary of the Ni^3+^/Ni^2+^ ratios in NiO_x_ without and with SAM modification from peak fitting of the nickel 2p3/2 core level spectra.

| **Samples** | **Ni species** | **BE**  **[eV]** | **Peak area** | **Ratio**  **[Ni^3+^/Ni^2+^]** |
| --- | --- | --- | --- | --- |
| NiO_x_ | Ni^3+^ | 856.08 | 60877.7 | 2.19 |
|  | Ni^2+^ | 854.04 | 27798.1 |  |
| NiO_x_/D3PAICz-1 | Ni^3+^ | 855.78 | 60787.6 | 2.85 |
|  | Ni^2+^ | 853.80 | 21390.4 |  |
| NiO_x_/M3PAICz-1 | Ni^3+^ | 855.40 | 46152.7 | 3.30 |
|  | Ni^2+^ | 853.55 | 21390.4 |  |
| NiO_x_/D3PAICz-2 | Ni^3+^ | 855.96 | 56055.2 | 2.87 |
|  | Ni^2+^ | 854.08 | 19601.4 |  |
| NiO_x_/M3PAICz-2 | Ni^3+^ | 855.87 | 63429.5 | 2.95 |
|  | Ni^2+^ | 853.92 | 21497.2 |  |

**
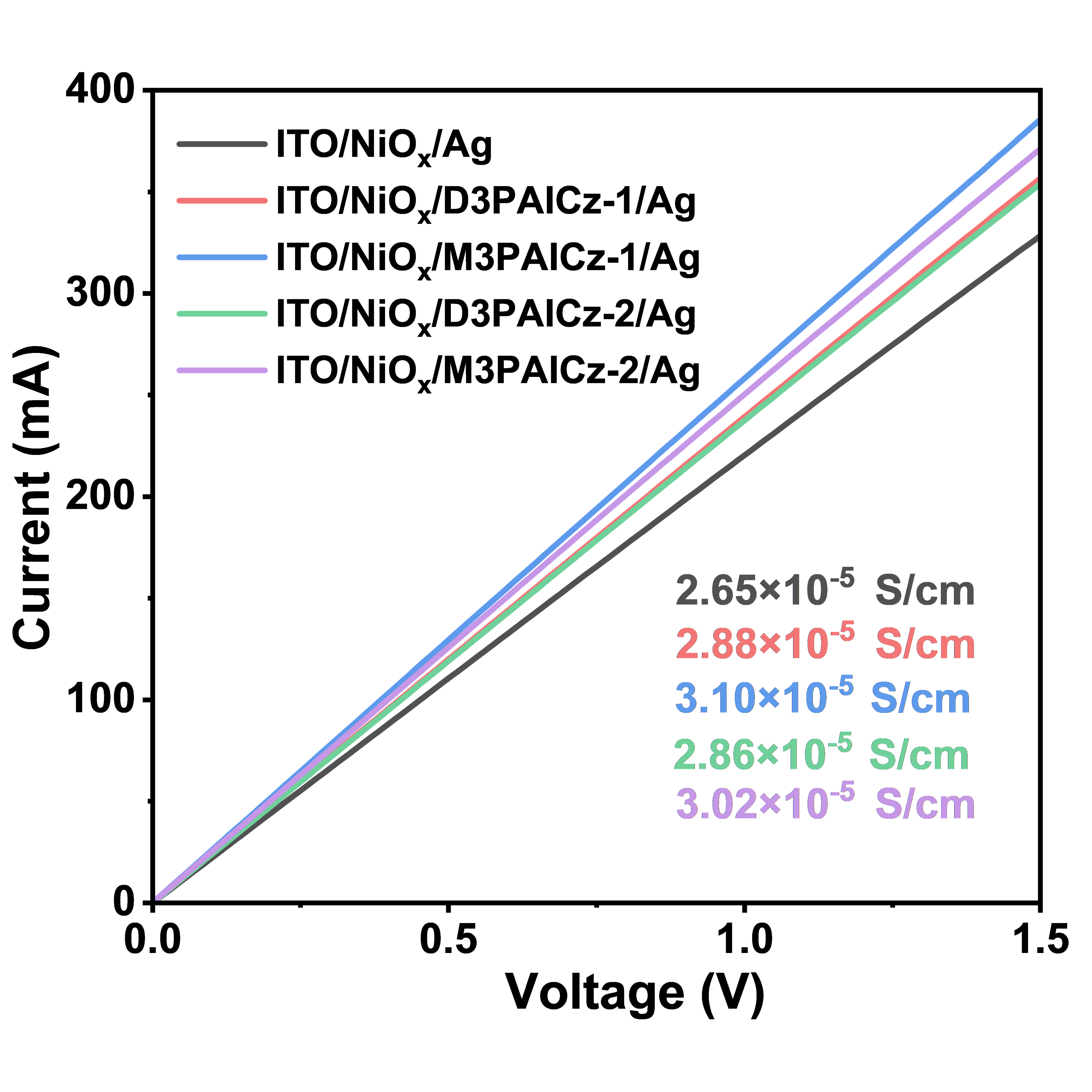
**

**Figure S48.** The conductivity of NiO_x_ films without and with SAM modification.


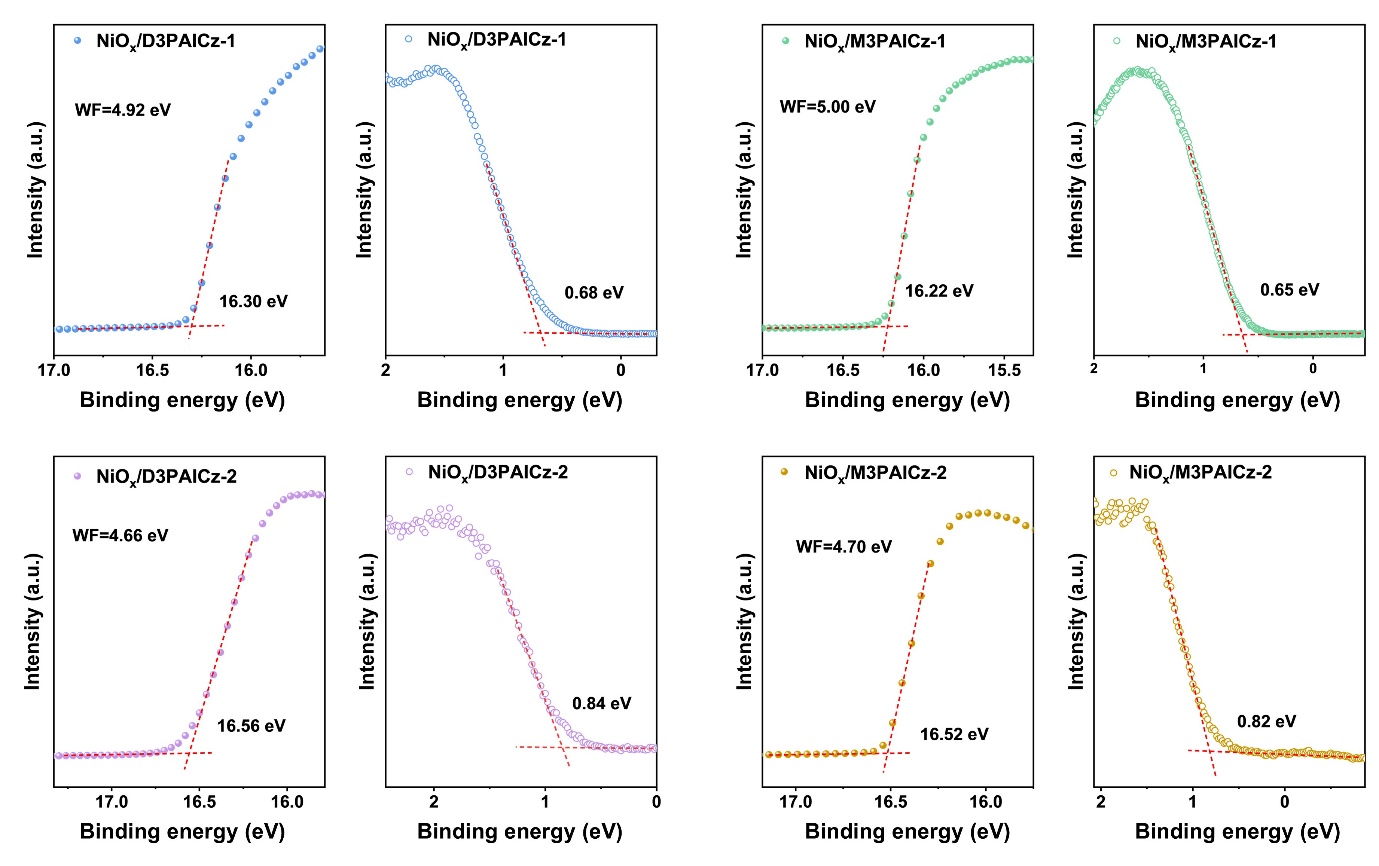


**Figure S49.** UPS spectra of different SAMs-modified NiO_x_

_
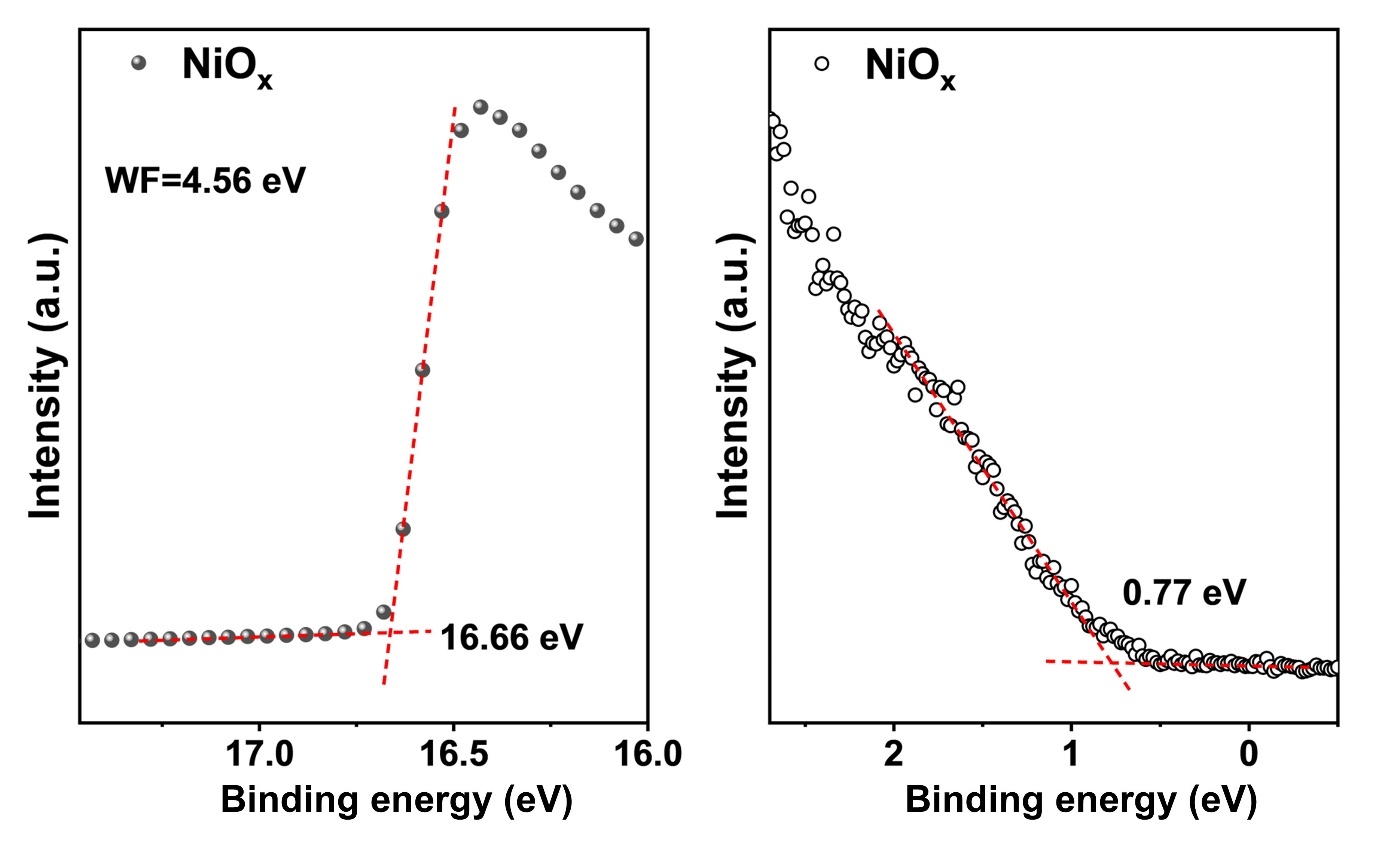
_

**Figure S50.** UPS spectra of bare NiO_x_.


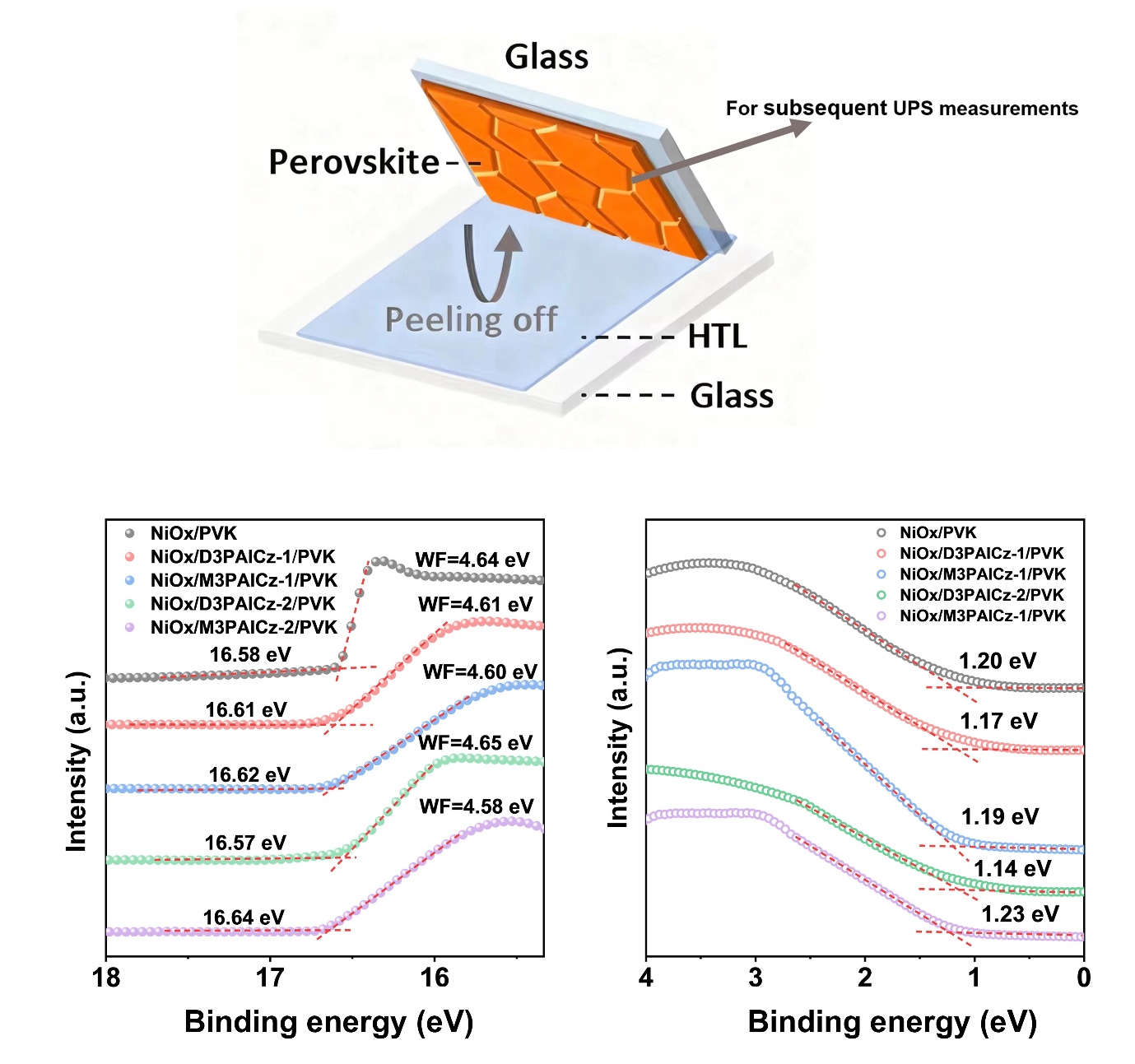


**Figure S51**. UPS spectra of the bottom interfaces of perovskite deposited on different HTLs.

**Table S4.** Experimentally measured energy levels of the SAMs.

| **SAMs** | **HOMO (eV)** | **HOMO (eV)** | **LUMO^(a)^** | ***E_g_*^opt^** |
| --- | --- | --- | --- | --- |
|  | **Measured by CV** | **Measured by UPS** | **[eV]** | **[eV]** |
| D3PAICz-1 | -5.32 | -5.60 | -2.10 | 3.22 |
| D3PAICz-1 | -5.39 | -5.65 | -2.15 | 3.24 |
| D3PAICz-2 | -5.13 | -5.50 | -2.19 | 2.94 |
| M3PAICz-2 | -5.18 | -5.52 | -2.23 | 2.95 |

^(a)^ LUMO =*E_g_*^opt^ (eV)+HOMO (CV)

**Table S5.** The surface CPD value of bare NiO_x_ along with those with different SAMs and the corresponding calculated WF values.

| **HTLs** | **CPD^(a)^ [mV]** | **WF ^(b)^ [eV]** |
| --- | --- | --- |
| NiO_x_/D3PAICz-1 | -794 | 5.24 |
| NiO_x_/M3PAICz-1 | -798 | 5.25 |
| NiO_x_/D3PAICz-2 | -564 | 5.01 |
| NiO_x_/M3PAICz-2 | -592 | 5.04 |
| Bare NiO_x_ | -523 | 4.97 |

1. The WF of tip is calibrated to be 4.75eV by the highly oriented pyrolytic graphite (HOPG) with fresh cleavage whose WF is 4.60 eV.
2. $\text{W}\text{F}^{\text{sample}}\text{=}{\text{WF}^{\text{tip}}\text{–e×CPD}}_{\text{sample}}$, where *e* is the charge of the electron.


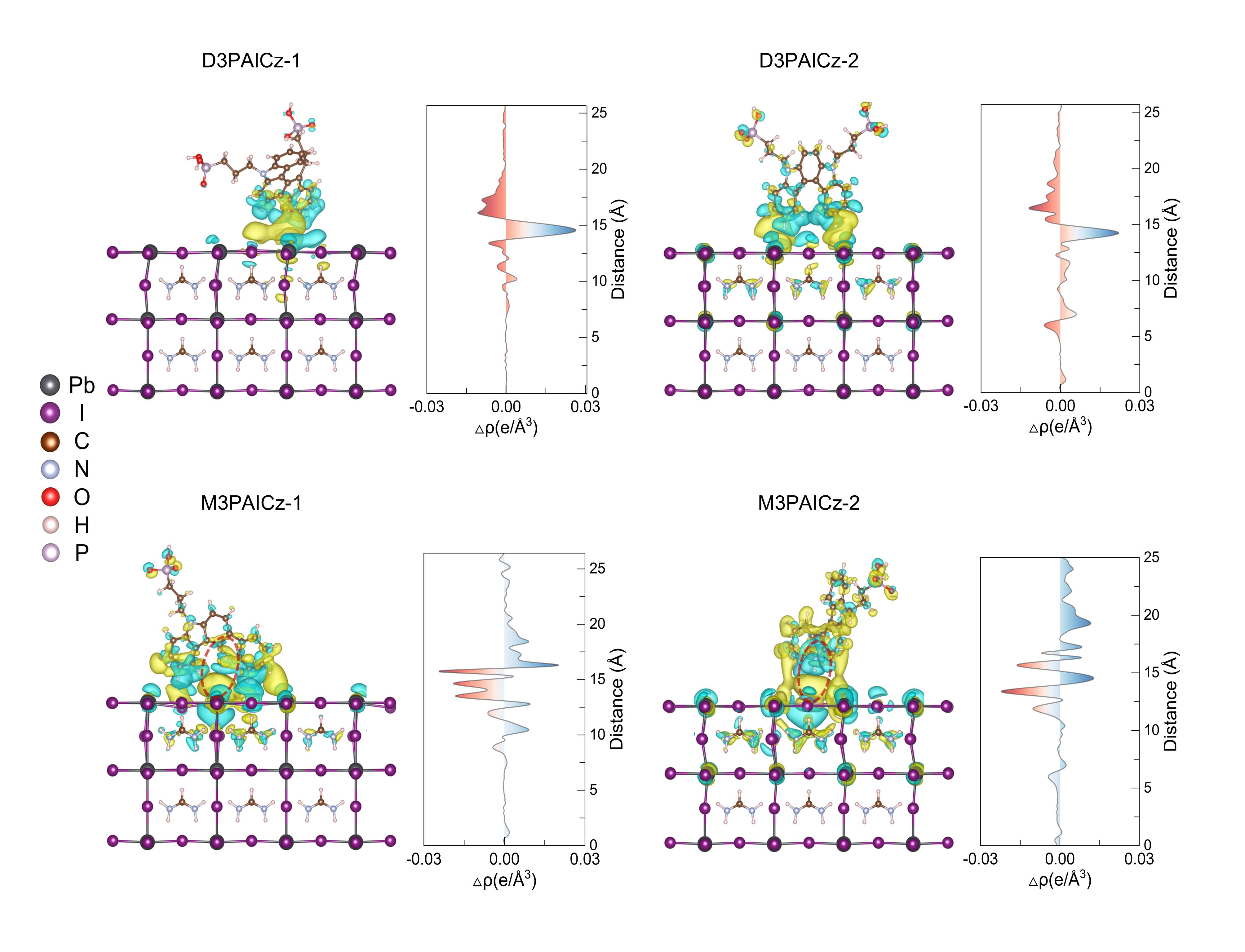


**Figure S52.** Differential charge density and its longitudinal integral curve between the four SAM molecules and PVK, as calculated by DFT. Blue iso-surface signifies electron depletion, while yellow represents electron accumulation. The red circles indicate charge transfer at the N-H…I hydrogen bond sites


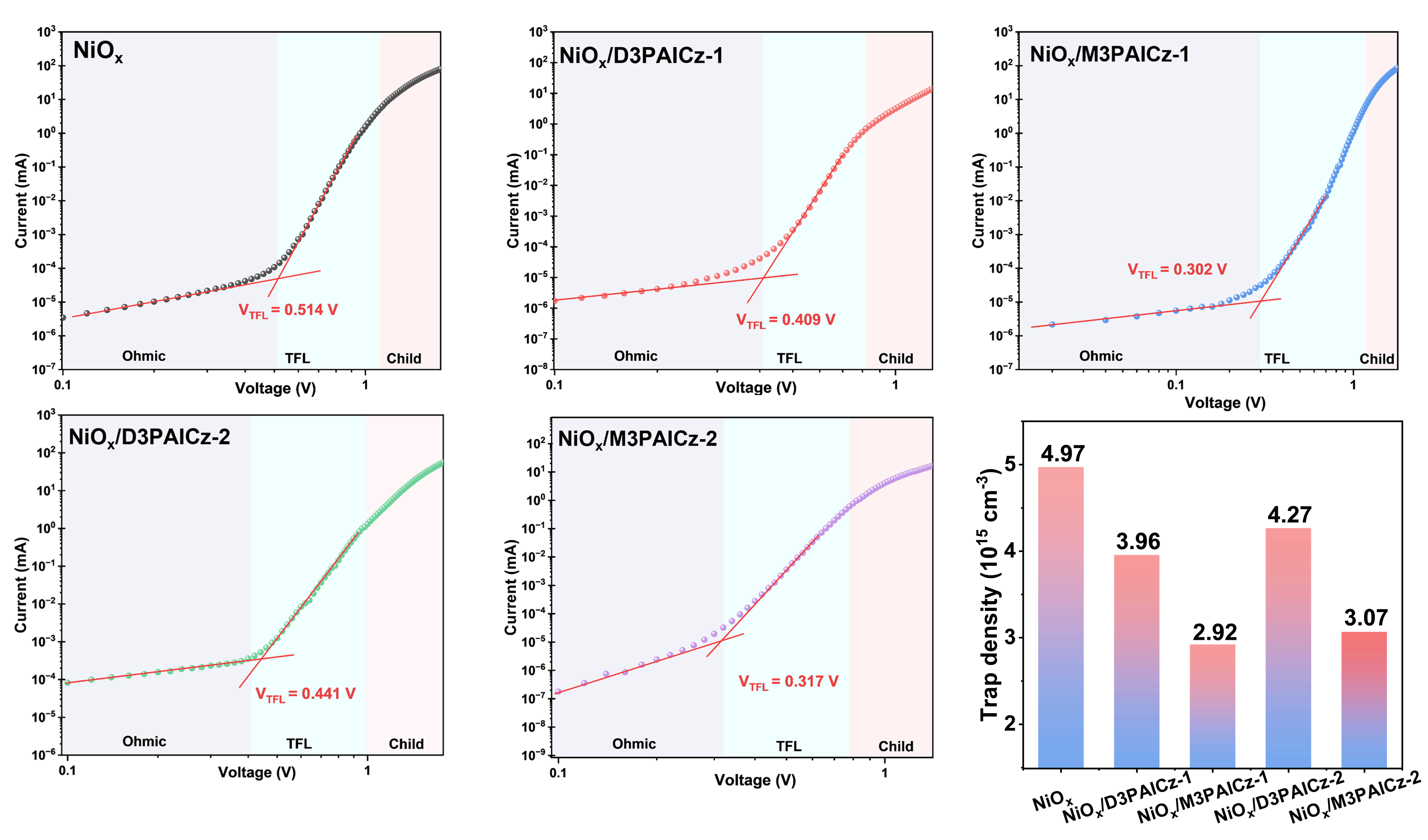


**Figure S53.** Trap densities in hole-only devices (ITO/HTLs/perovskite/spiro-OMeTAD/Ag) quantified by SCLC measurements, with perovskite layer thicknesses verified via cross-sectional SEM image in **Figure S54**.


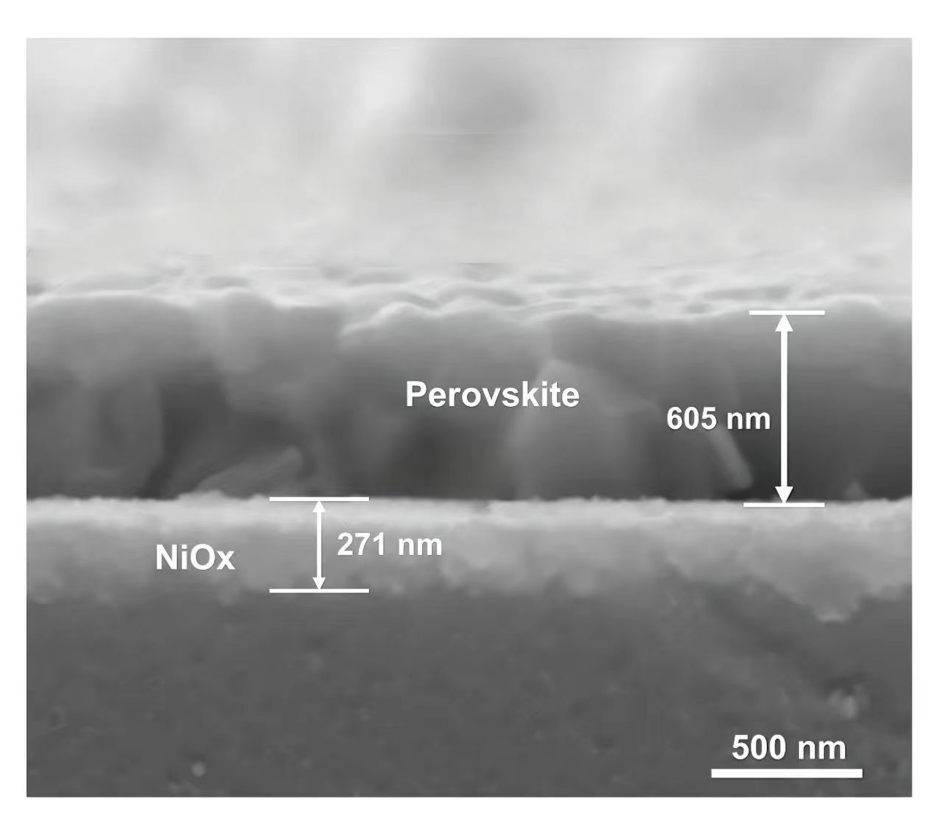


**Figure S54.** The cross-sectional SEM image of perovskite.


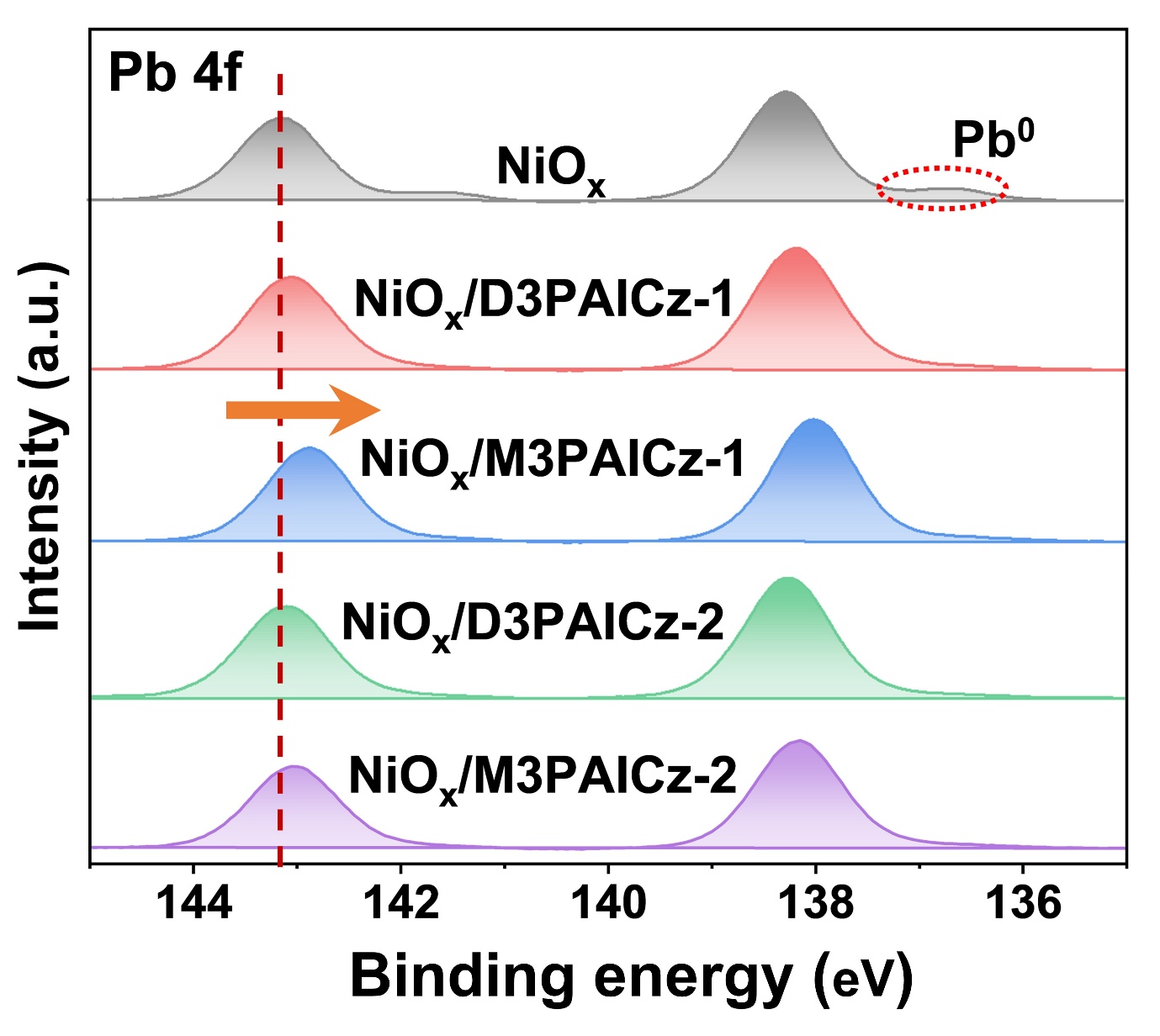


**Figure S55.** Pb 4f XPS for the perovskite films deposited on the different HTLs.


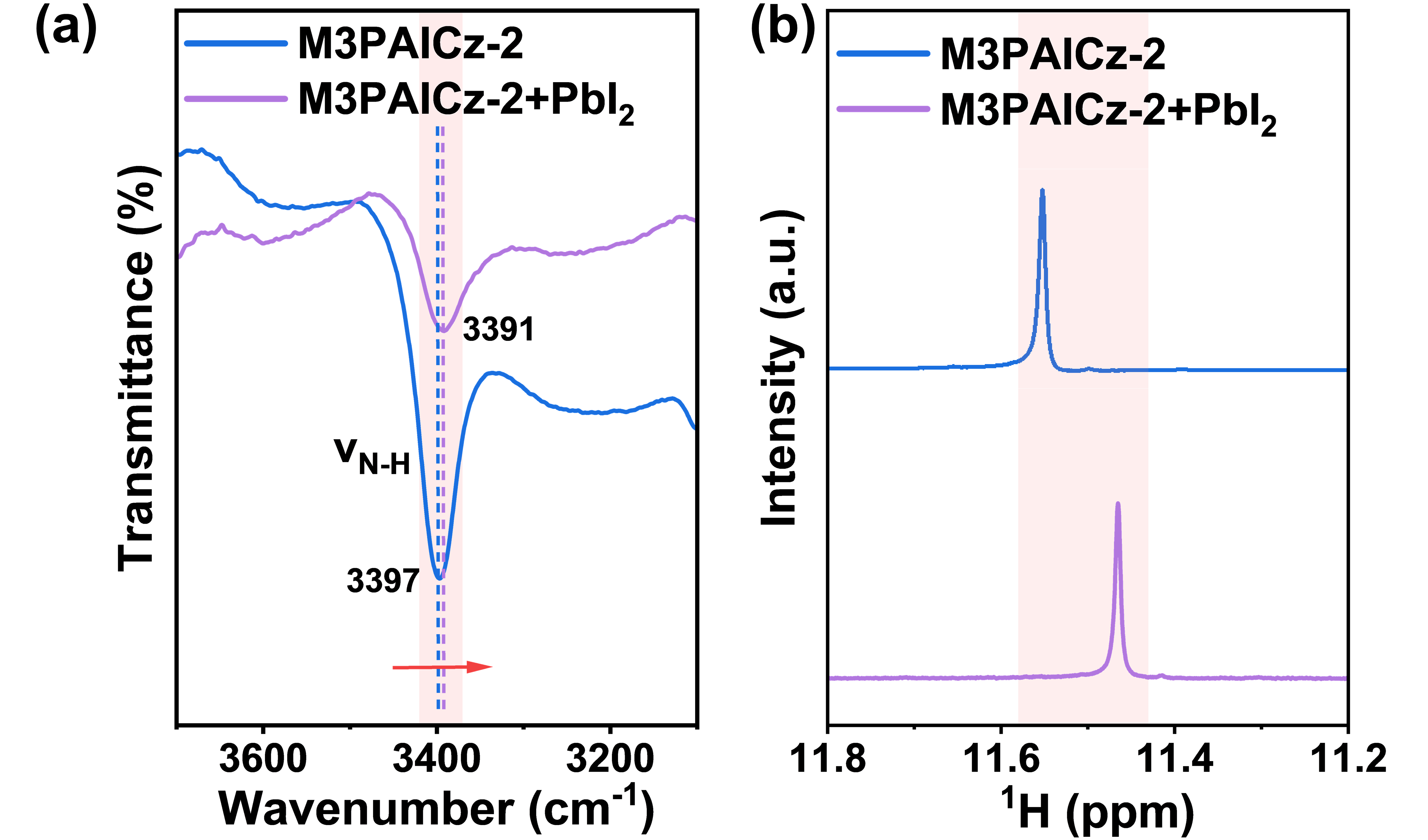


**Figure S56.** FTIR spectra of M3PAICz-1 and PbI_2_+M3PAICz-1 composite materials.


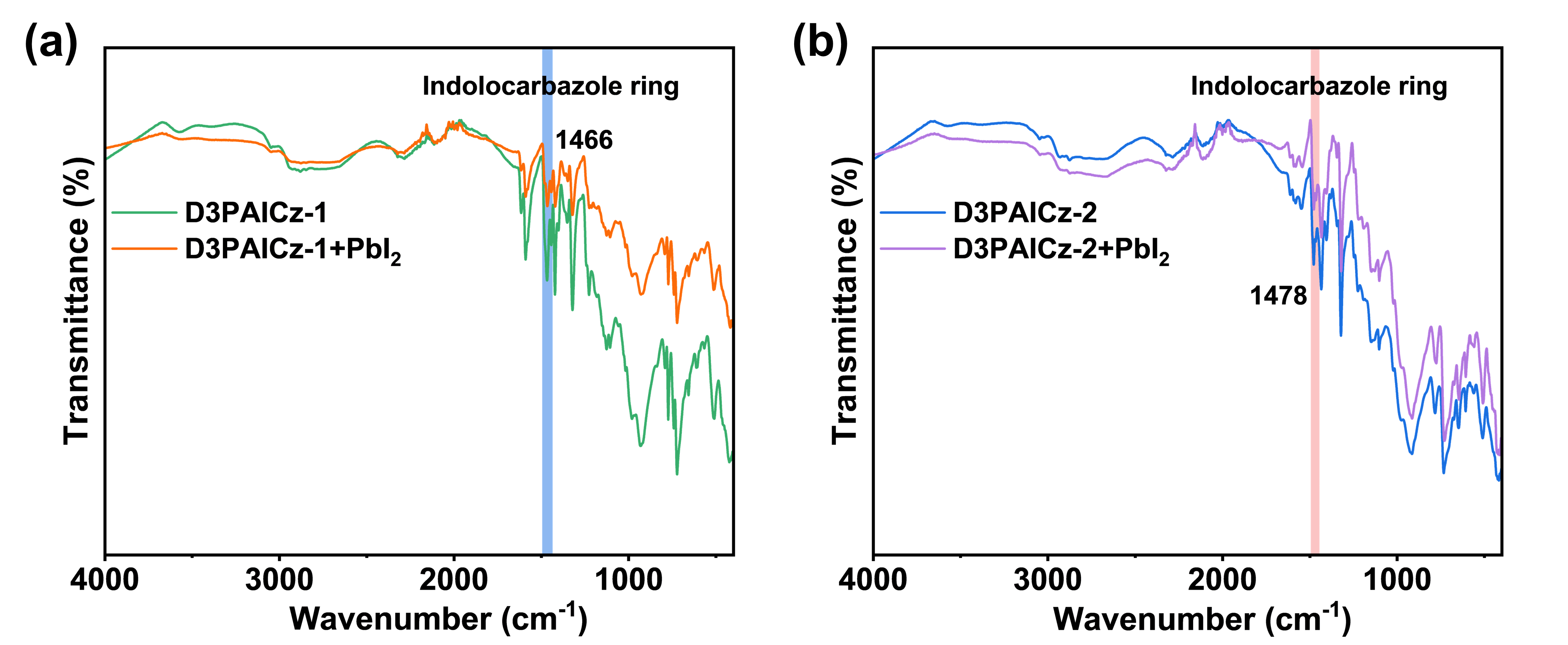


**Figure S57.** FTIR spectra of (a) D3PAICz-1 and PbI2+D3PAICz-1 composite materials or (b) D3PAICz-1 and PbI_2_+D3PAICz-2 composite materials.


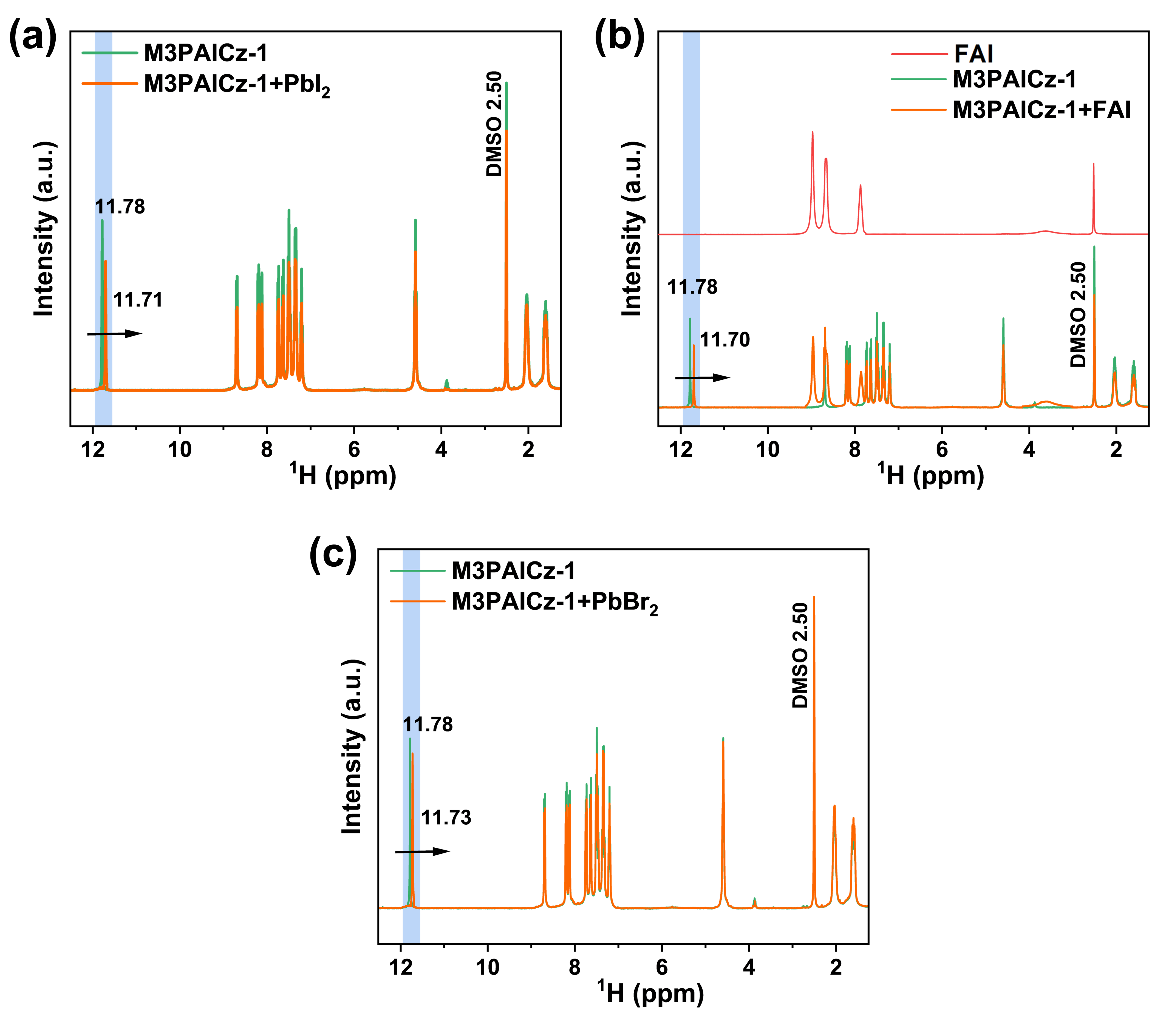


**Figure S58.** The ^1^H NMR spectra of M3PAICz-1 before and after interactions with (a) FAI, (b) PbI_2_ and (c) PbBr_2_.


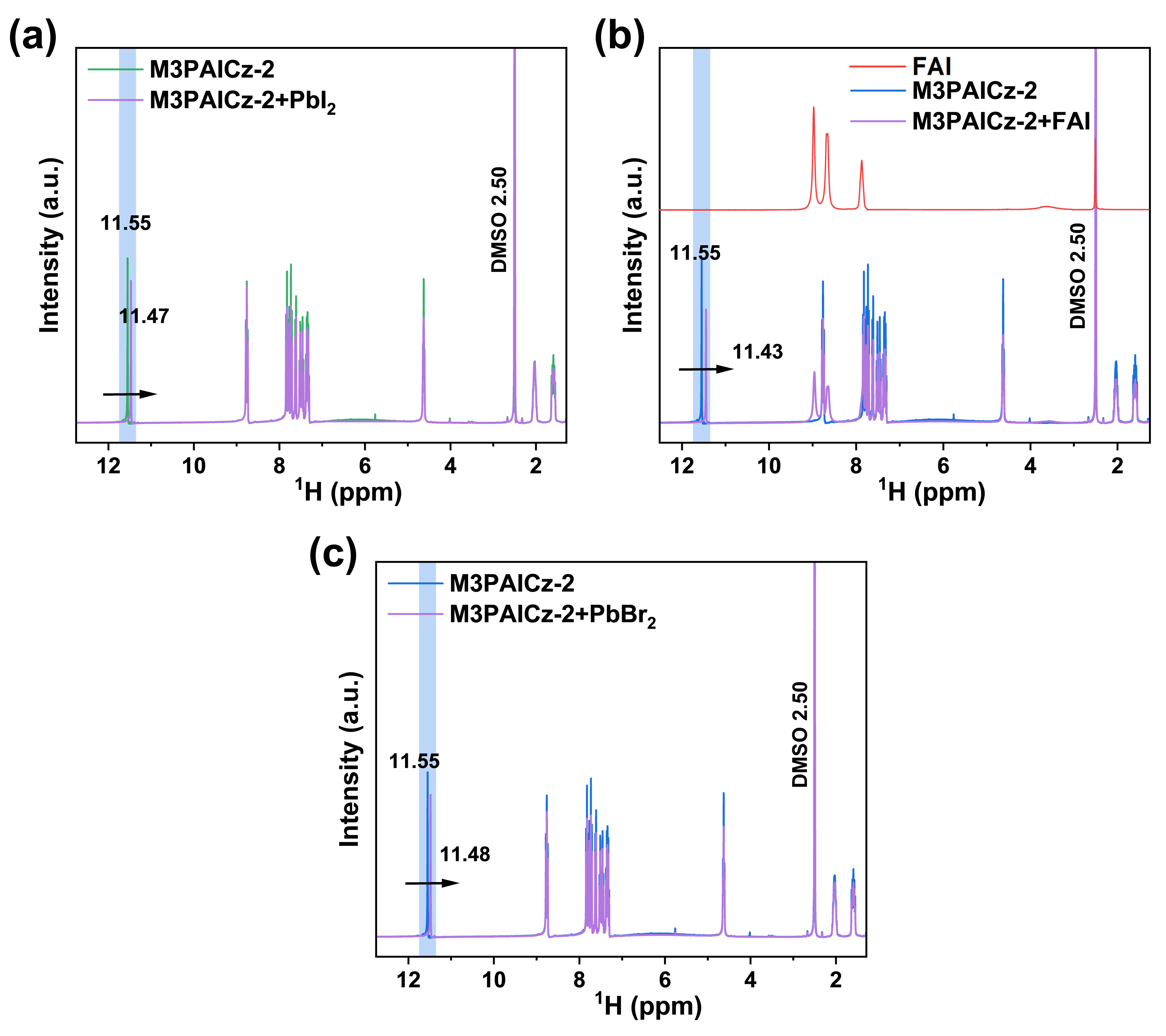


**Figure S59.** The ^1^H NMR spectra of M3PAICz-2 before and after interactions with (a) FAI, (b) PbI_2_ and (c) PbBr_2_.


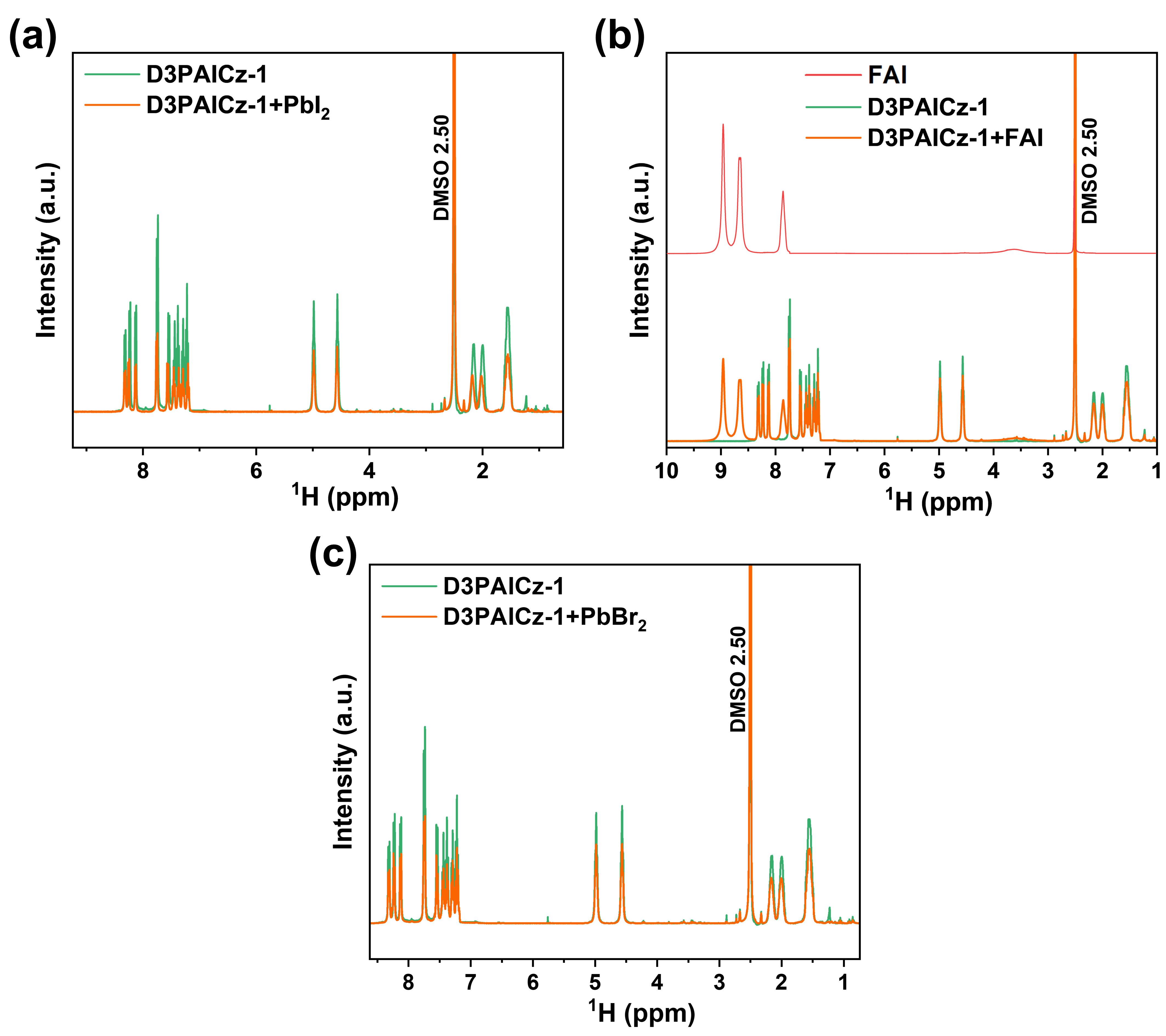


**Figure S60.** The ^1^H NMR spectra of D3PAICz-1 before and after interactions with (a) FAI, (b) PbI_2_ and (c) PbBr_2_.


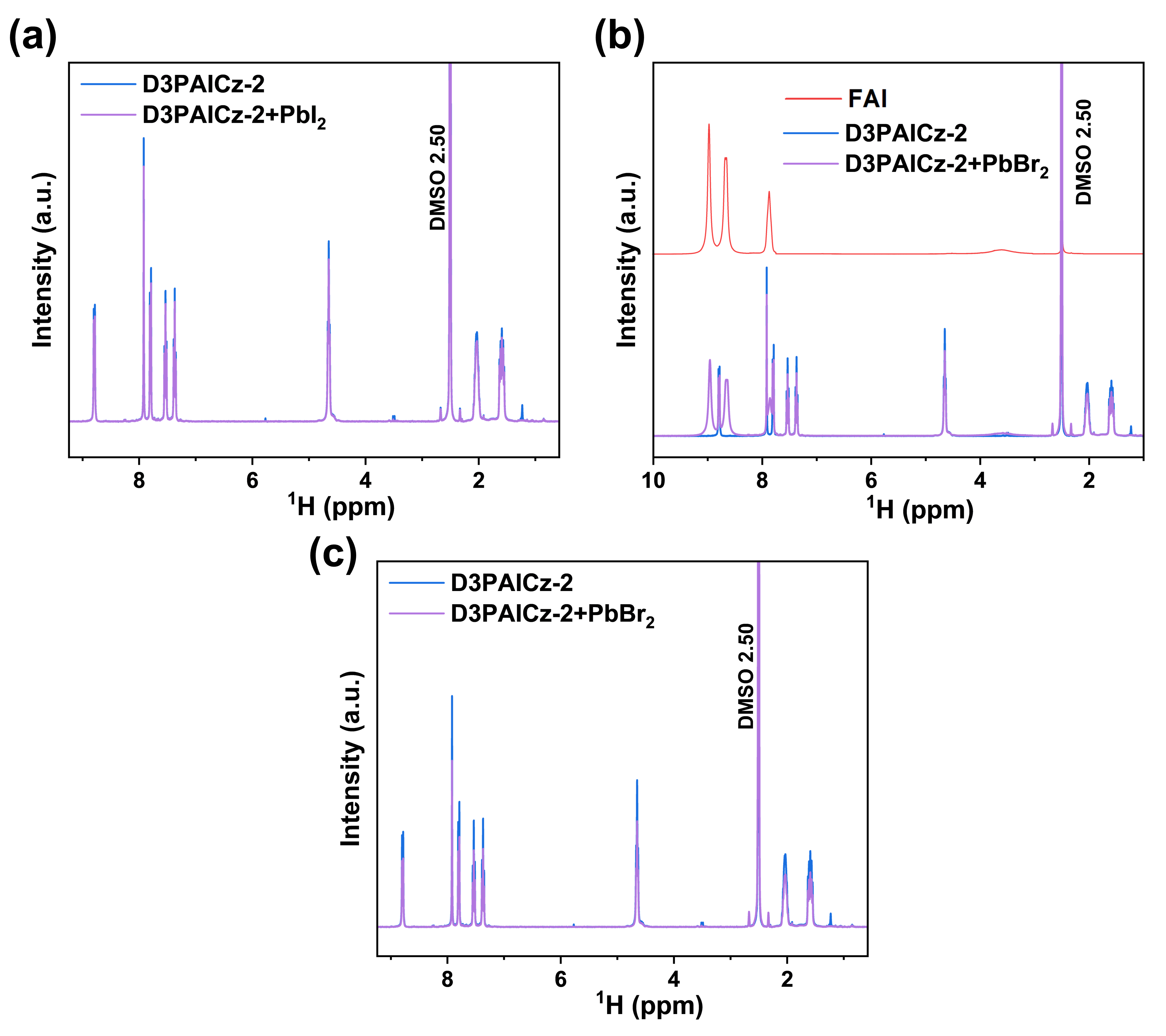


**Figure S61.** The ^1^H NMR spectra of D3PAICz-2 before and after interactions with (a) FAI, (b) PbI_2_ and (c) PbBr_2_.


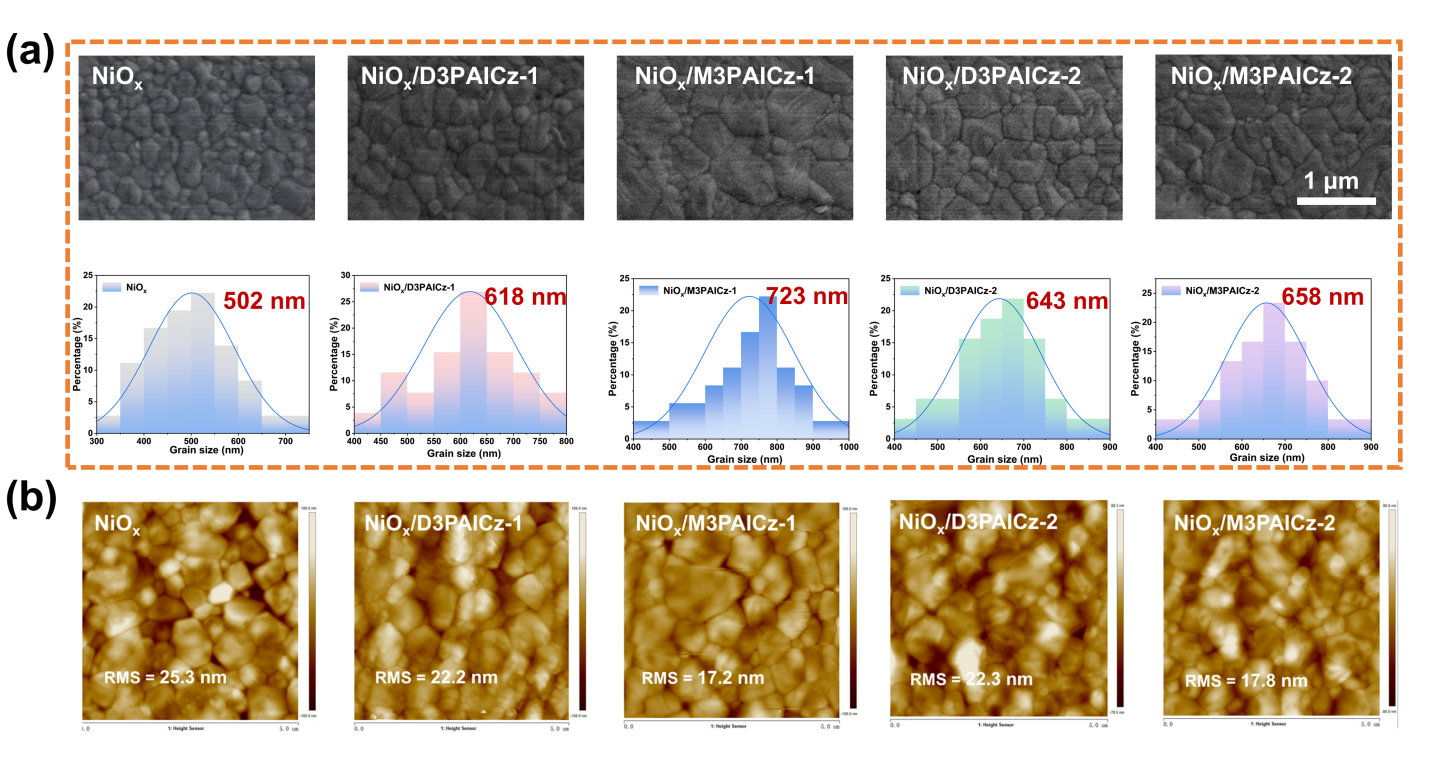


**Figure S62. (**a) Top-view SEM images with corresponding grain size distributions (mean values indicated) and (b) AFM images of perovskite films grown on various HTLs.


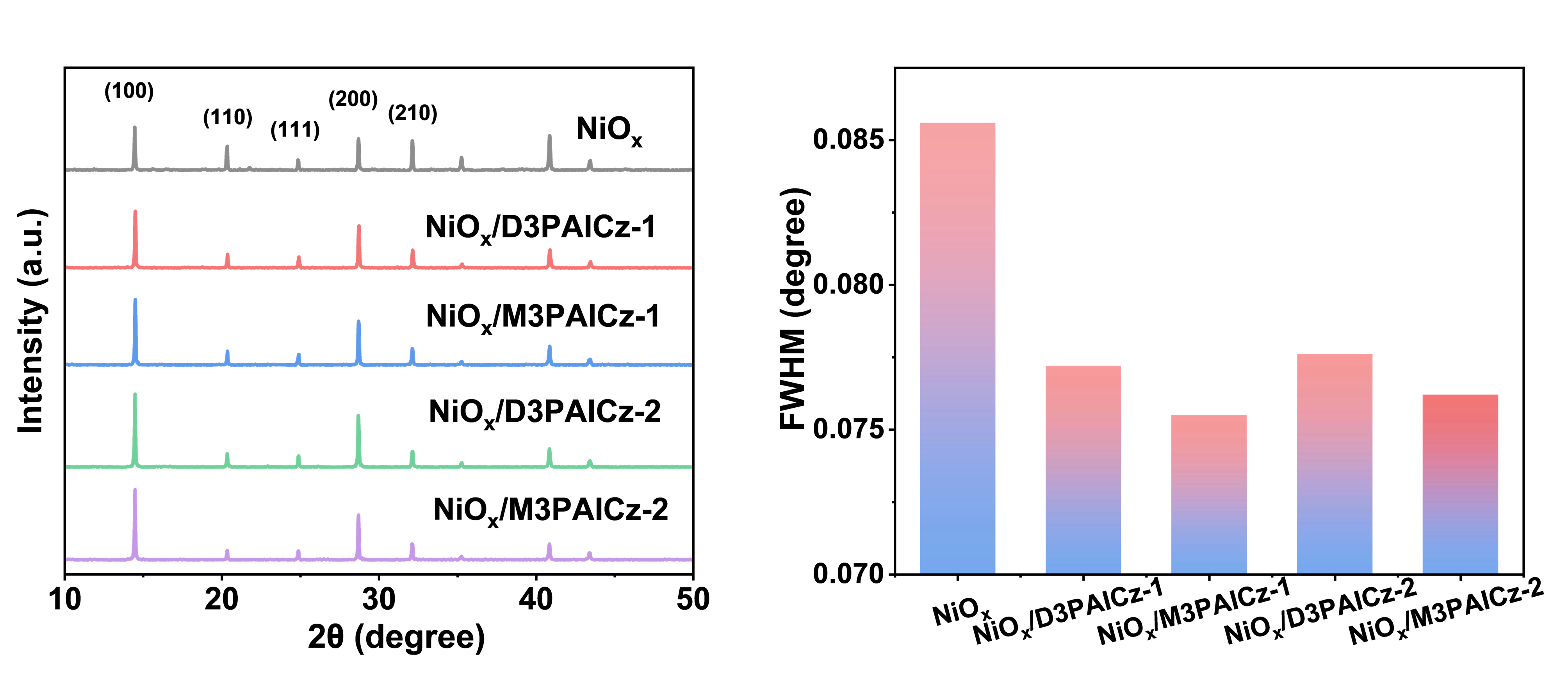


**Figure S63.** XRD patterns of perovskite films on different HTLs with the corresponding full width at half maximum (FWHM) values of the (100) diffraction peak.


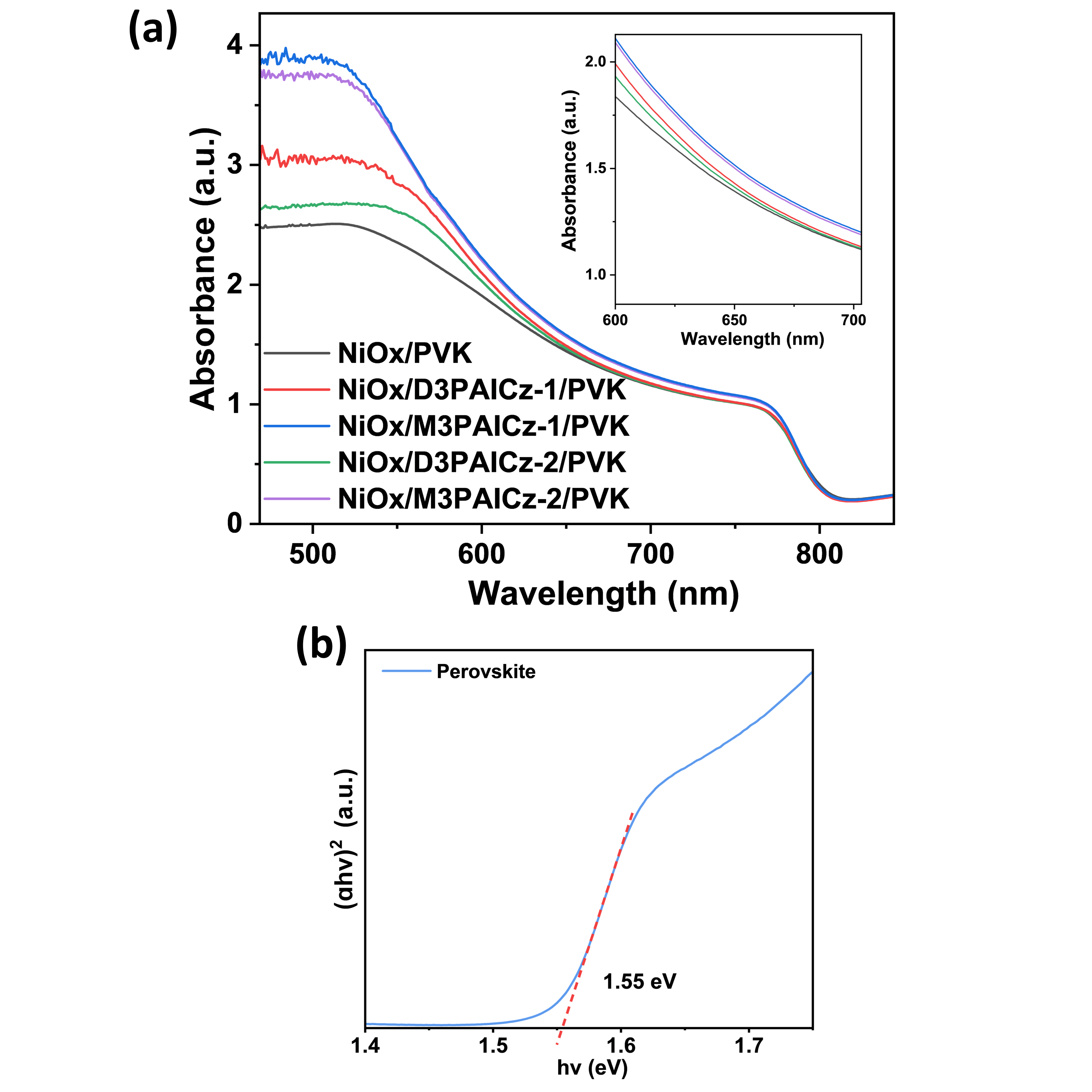


**Figure S64.** (a) UV-vis absorption spectra and (b) the corresponding Tauc plot of the perovskite Cs_0.05_(FA_0.95_MA_0.05_)_0.95_Pb(I_0.95_Br_0.05_)_3_ films on different HTLs.

**Table S6.** The fitted carrier lifetime of perovskite films obtained from TRPL measurements.

| **HTLs** | **A_1_**  **[%]** | **τ_1_**  **[ns]** | **A_2_**  **[%]** | **τ**  **[ns]** | **τ_avg_^(a)^**  **[ns]** |
| --- | --- | --- | --- | --- | --- |
| NiO_x_/D3PAICz-1 | 0.30 | 28.92 | 99.70 | 724.43 | 722.3 |
| NiO_x_/M3PAICz-1 | 9.62 | 23.02 | 90.38 | 172.68 | 158.3 |
| NiO_x_/D3PAICz-2 | 7.44 | 67.71 | 92.56 | 1002.78 | 933.2 |
| NiO_x_/M3PAICz-2 | 2.57 | 18.75 | 75.43 | 262.02 | 198.1 |
| Bare NiO_x_ | 11.96 | 131.28 | 88.04 | 1334.27 | 1190.44 |

^(a)^ $\text{τ}_{\text{avg}}\text{=(}\text{A}_{\text{1}}\text{×}\text{τ}_{\text{1}}^{\text{2}}\text{+}\text{A}_{\text{2}}\text{×}\text{τ}_{\text{2}}^{\text{2}}\text{)/(}\text{A}_{\text{1}}\text{×}\text{τ}_{\text{1}}\text{+}\text{A}_{\text{2}}\text{×}\text{τ}_{\text{2}}\text{)}$

**Table S7.** The summary of all fitted value for PSCs based on different SAMs from EIS results.

| **HTLs** | ***R_s_***  **[Ω]** | ***R_rec_***  **[Ω]** |
| --- | --- | --- |
| NiO_x_/D3PAICz-1 | 34.46 | 29372 |
| NiO_x_/M3PAICz-1 | 8.59 | 44125 |
| NiO_x_/D3PAICz-2 | 26.82 | 21509 |
| NiO_x_/M3PAICz-2 | 27.2 | 36200 |
| Bare NiO_x_ | 39.48 | 5275 |


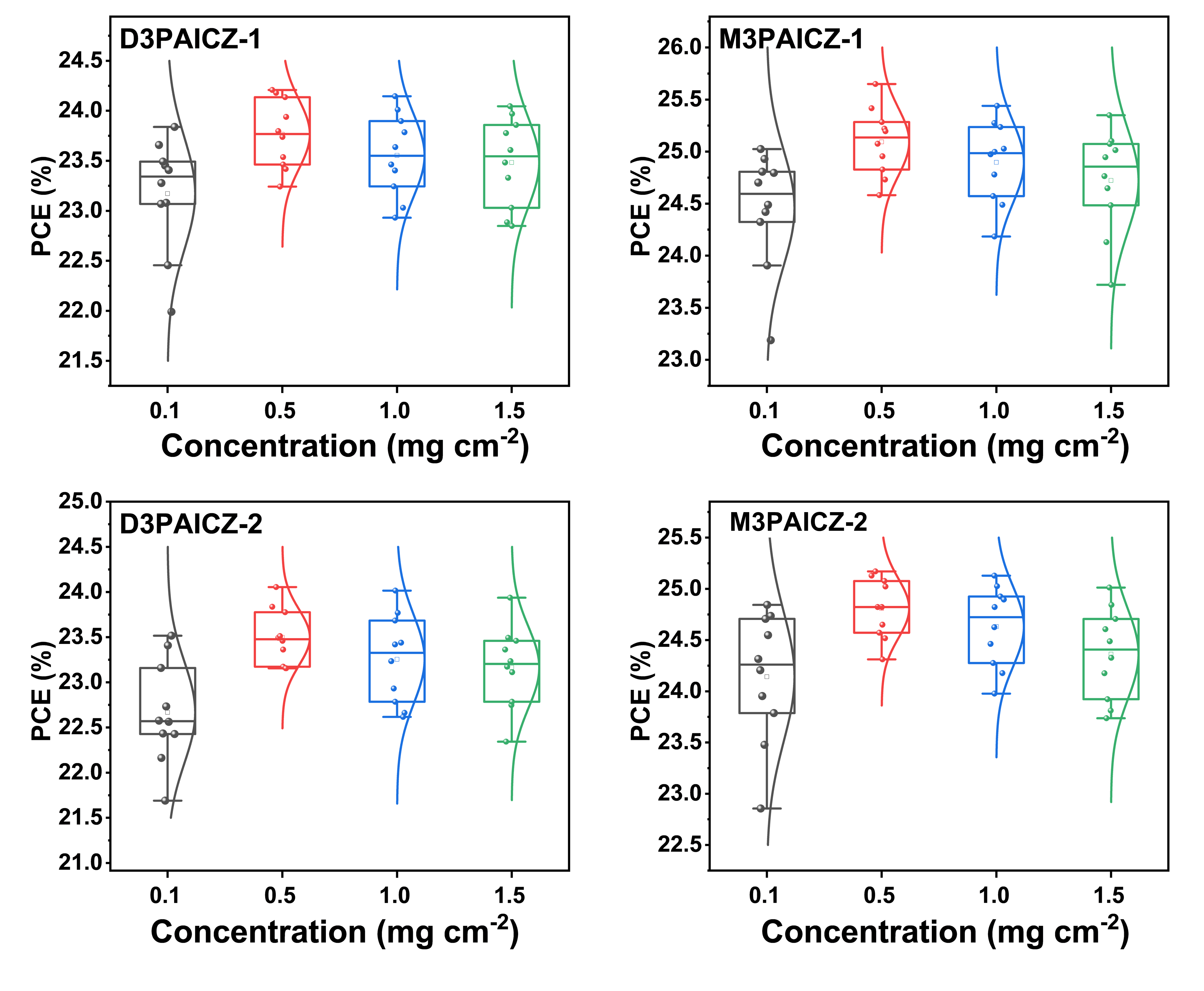


**Figure S65.** PCE statistical distribution of NiO_x_/SAM-based devices with different concentrations of these SAMs..

**Table S8.** Summary of the photovoltaic parameters of the champion PSCs based on different HTLs.

| **HTLs** | **Scan**  **direction** | ***V_OC_*** | ***J_SC_*** | **FF** | **PCE** | **Integrated *J_SC_*** |
| --- | --- | --- | --- | --- | --- | --- |
|  |  | **[V]** | **[mAcm-2]** | **[%]** | **[%]** | **[mA cm-2]** |
| NiO_x_ | Reverse | 1.078 | 25.65 | 80.29 | 22.20 | 25.22 |
|  | Forward | 1.033 | 25.59 | 74.03 | 19.57 |  |
| NiO_x_/D3PAICz-1 | Reverse | 1.114 | 26.01 | 84.03 | 24.34 | 25.44 |
|  | Forward | 1.113 | 25.78 | 79.44 | 22.79 |  |
| NiO_x_/M3PAICz-1 | Reverse | 1.163 | 26.11 | 84.41 | 25.64 | 25.71 |
|  | Forward | 1.149 | 26.10 | 81.61 | 24.48 |  |
| NiO_x_/M3PAICz-1^a)^ | Reverse | 1.180 | 26.18 | 84.60 | 26.12 |  |
|  | Forward | 1.157 | 26.19 | 81.42 | 24.67 |  |
| NiO_x_/D3PAICz-2 | Reverse | 1.097 | 26.00 | 84.15 | 24.01 | 25.39 |
|  | Forward | 1.110 | 25.69 | 80.05 | 22.61 |  |
| NiO_x_/M3PAICz-2 | Reverse | 1.151 | 26.05 | 84.19 | 25.24 | 25.69 |
|  | Forward | 1.141 | 26.03 | 82.00 | 24.35 |  |
| NiO_x_/2PACz | Reverse | 1.134 | 25.90 | 80.67 | 23.70 | 25.30 |
|  | Forward | 1.118 | 25.53 | 75.56 | 21.56 |  |

1. The NiO_x_/M3PAICz-1-based PSCs using pre-synthesized single crystals as solutes and passivated the interface with 2D perovskite.


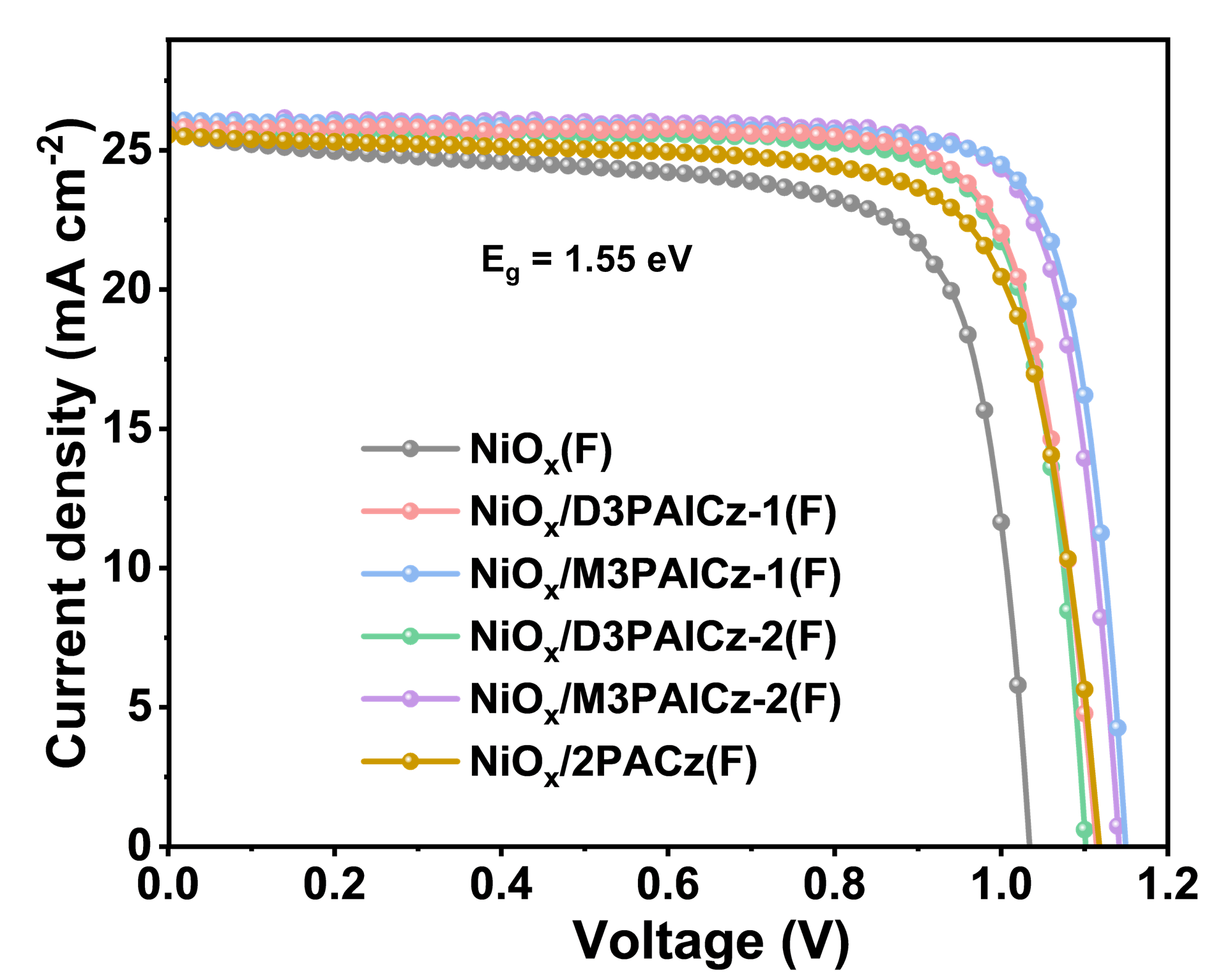


**Figure S66.** *J-V* curves of the best cells based on different HTLs in forward scan.


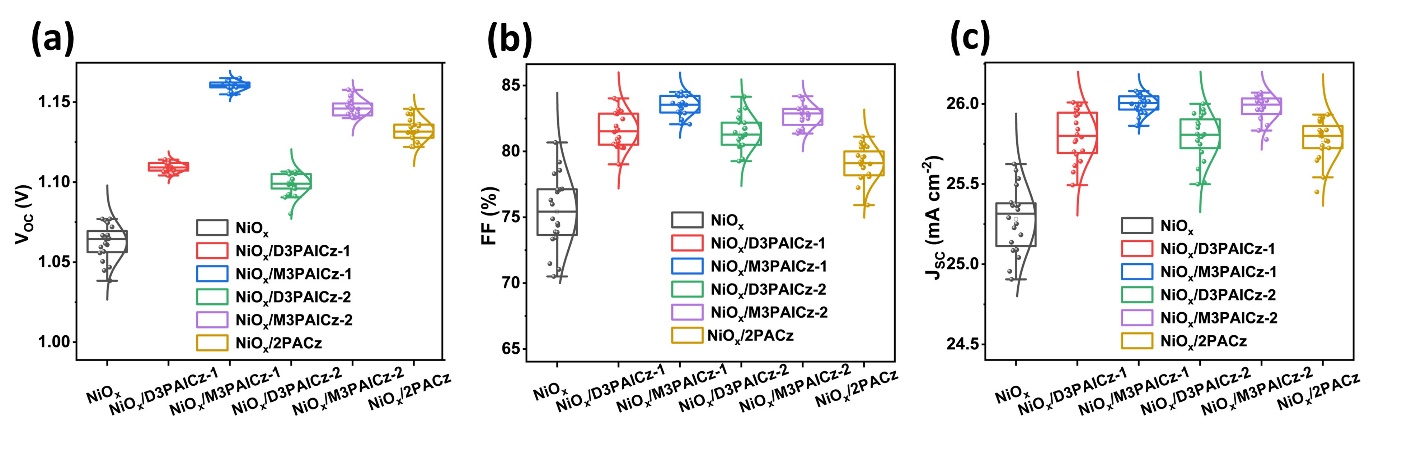


**Figure S67.** The statistics of (a) *V_OC_*, (b) FF, and (c) *J_SC_* values of the devices based on different HTLs.

**Note S2.** Synthesis and characterizations of single crystals

We synthesized MAPbBr_3_ single crystals by inverse temperature crystallization (ITC) method. Bright orange MAPbBr_3_ crystals are grown at 100 ℃ in the third hour of crystallization. The nanocubes are partly inlaid because of fast heating of solution and high solution concentration. Tetragonal MAPbBr_3_ crystals with the side-length of 3-6 mm are grown, dried and collected (**Figure S66a**).To verify the purity of products, we ground single crystals to powder and performed XRD measurements (**Figure S66b**), the XRD pattern of MAPbBr_3_ matches well with previous studies.


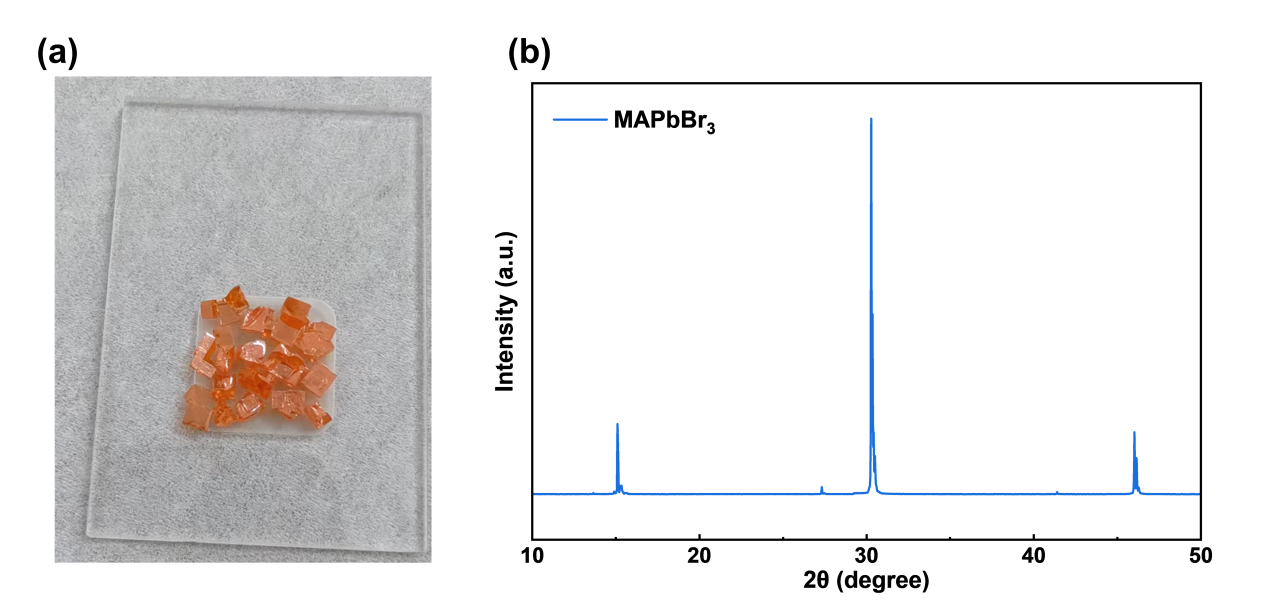


**Figure S68.** The (a) Image and (b) XRD pattern of monocrystalline MAPbBr_3_


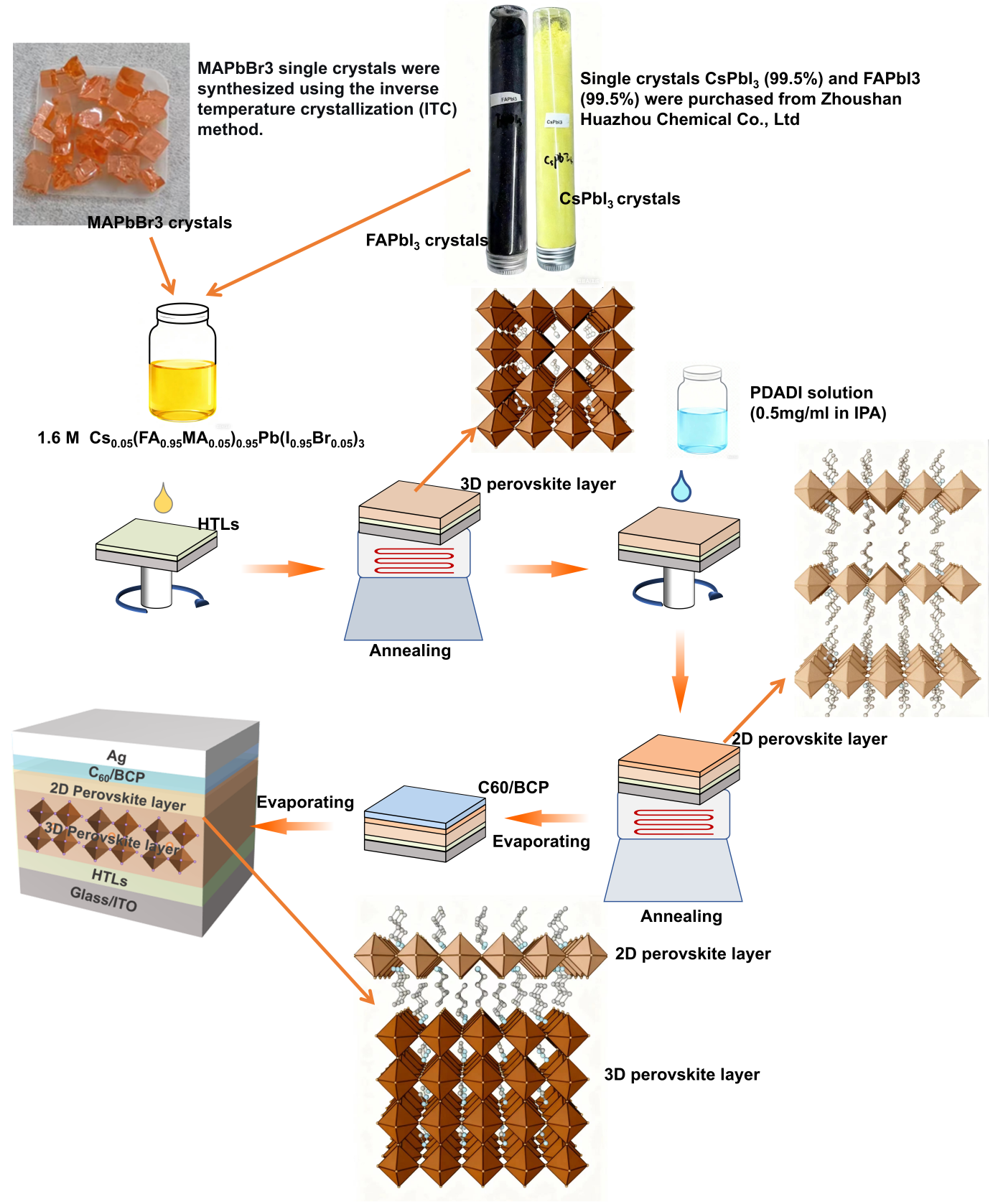


**Figure S69.** Schematic of device fabrication utilizing pre-synthesized single crystals as solutes and a two-dimensional (2D) perovskite passivated interface.


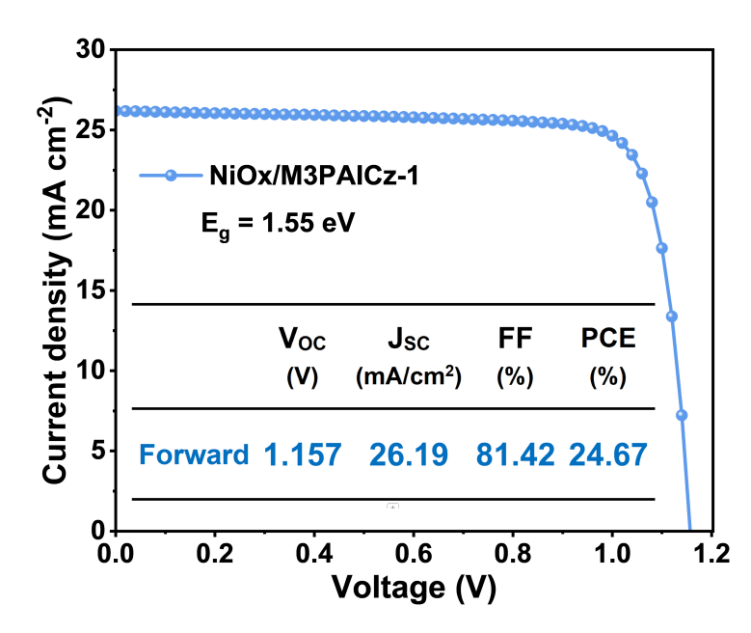


**Figure S70.** *J-V* curves of the NiO_x_/M3PAICz-1-based PSCs using presynthesized single crystals as solutes and passivated the interfaces with 2D perovskite in forward scan.


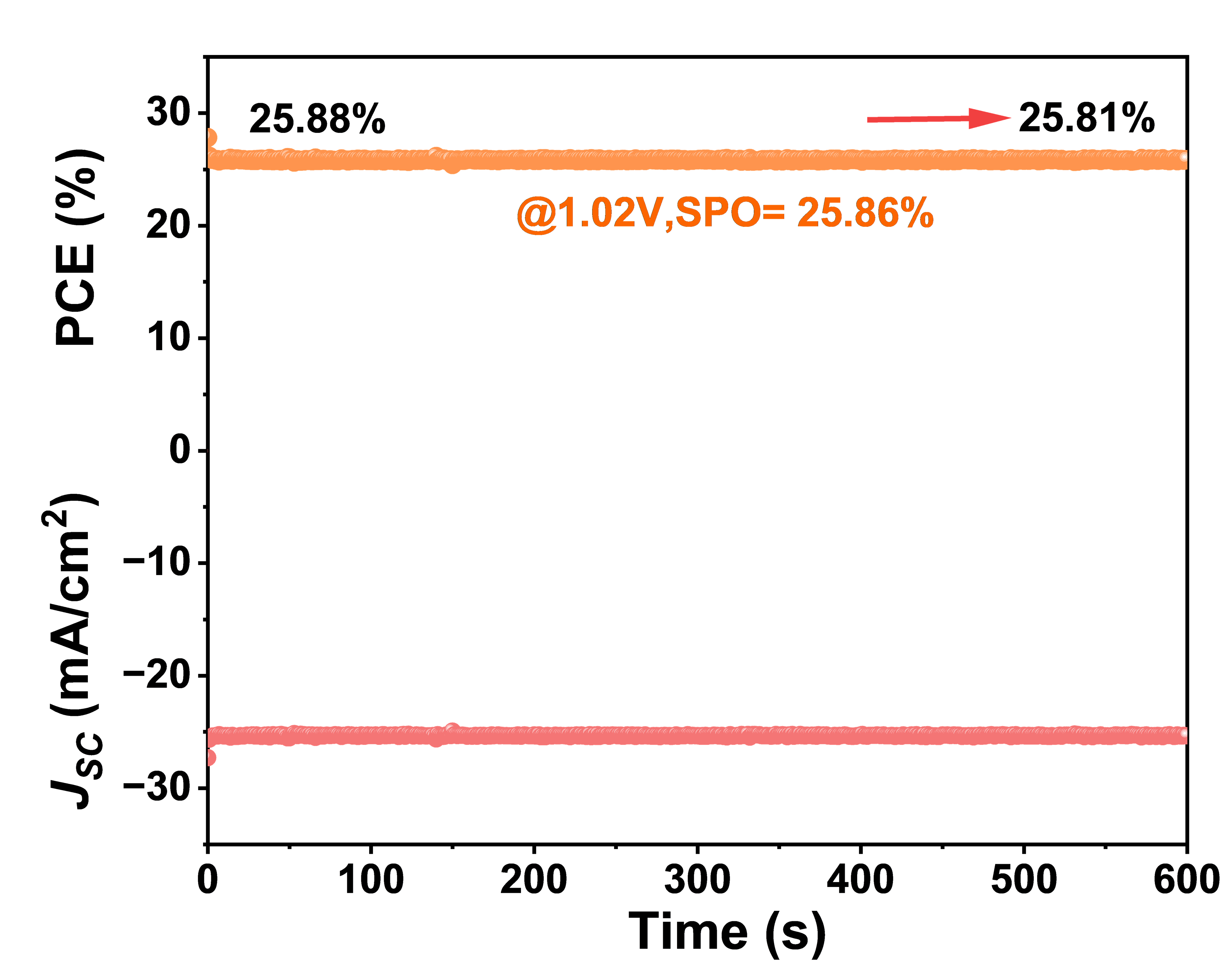


**Figure S71.** SPO of the NiO_x_/M3PAICz-1-based PSCs using presynthesized single crystals as solutes and passivated the interfaces with 2D perovskite.


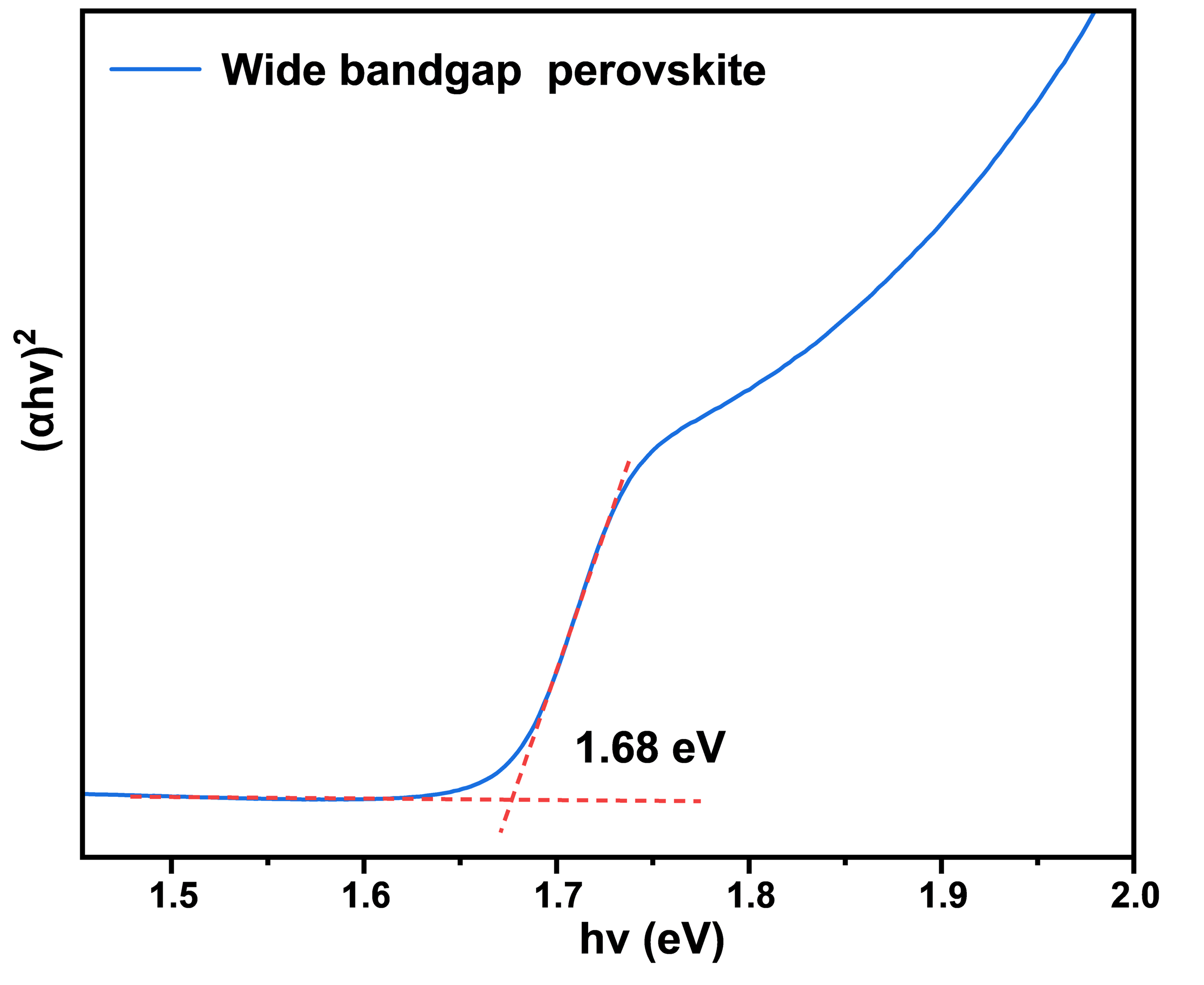


**Figure S72.** The Tauc plot of the 1.68 eV wide-bandgap perovskite Cs_0.05_FA_0.73_MA_0.22_Pb(I_0.77_Br_0.23_)_3_ film.


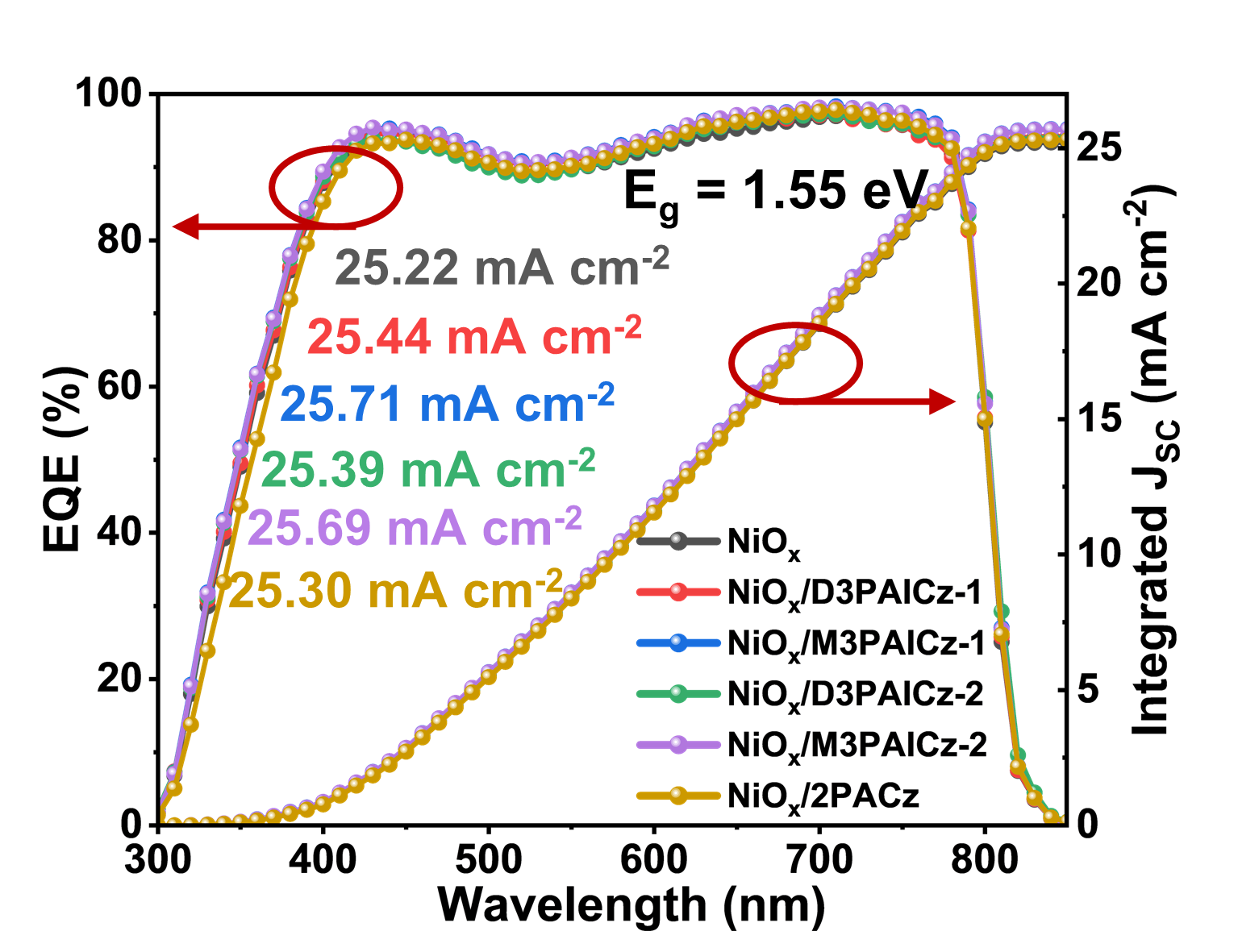


**Figure S73.** The statistics of EQEs and integrated current density curves of the corresponding 1.55 eV bandgap PSCs


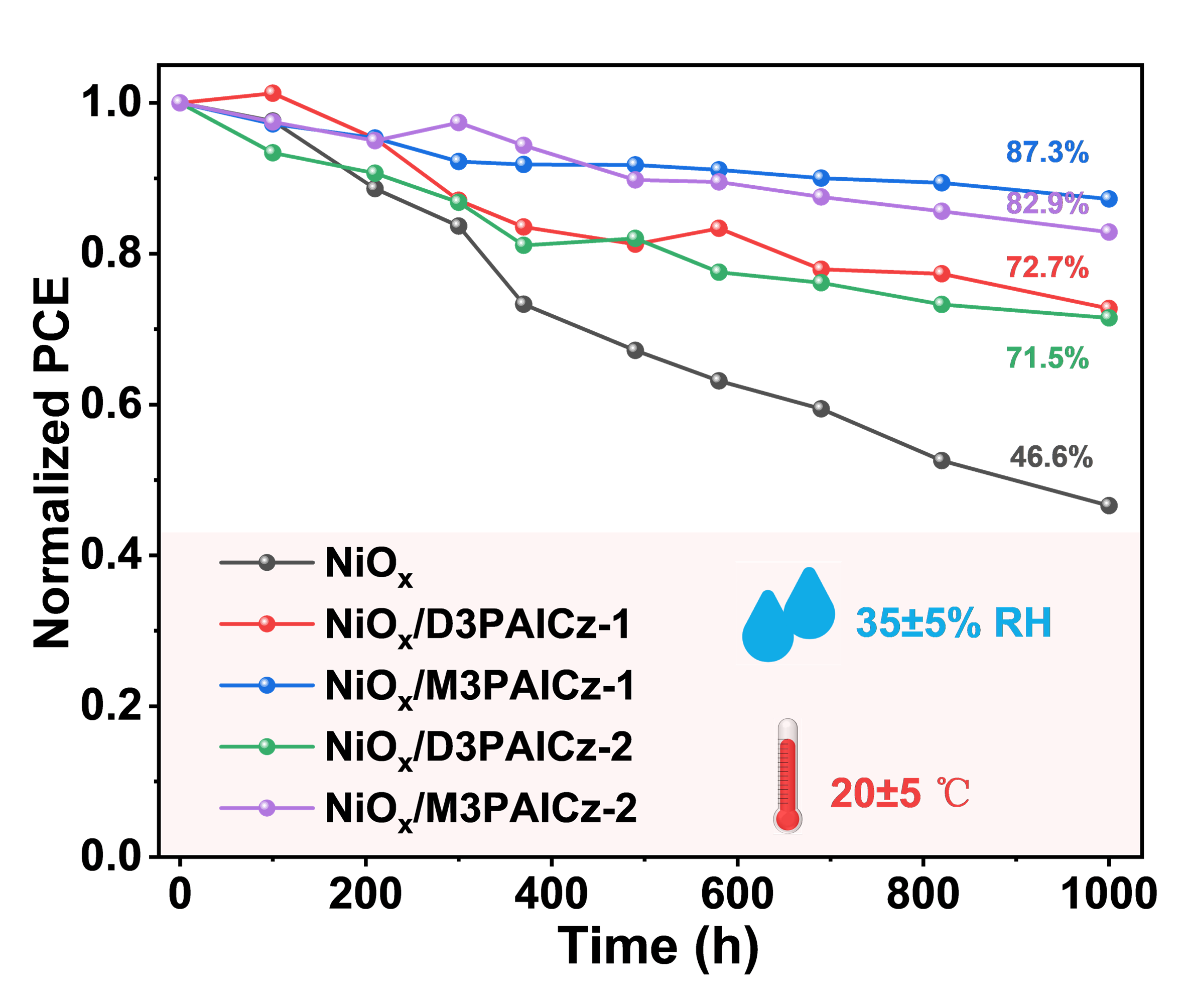


**Figure S74.** The PCE degradation curves of PSCs based on different HTLs at room temperature in ambient air.

**Table S9.** Summary of performance of wide-bandgap inverted PSCs.

| **Time** | **Bandgap**  [eV] | **PCE**  [%] | ***V_OC_***  [V] | **FF**  [%] | ***J_SC_***  [mA cm^-2^] | **Ref.** |
| --- | --- | --- | --- | --- | --- | --- |
| 2025 | 1.68 | 20.90 | 1.23 | 77.15 | 22.09 | ^[7]^ |
| 2025 | 1.68 | 20.95 | 1.20 | 83.60 | 20.90 | ^[8]^ |
| 2025 | 1.66 | 22.53 | 1.24 | 84.27 | 21.59 | ^[9]^ |
| 2024 | 1.68 | 21.86 | 1.21 | 86.18 | 20.93 | ^[10]^ |
| 2024 | 1.68 | 21.30 | 1.19 | 82.30 | 21.70 | ^[11]^ |
| 2024 | 1.68 | 22.35 | 1.22 | 82.87 | 22.18 | ^[12]^ |
| 2024 | 1.68 | 22.06 | 1.25 | 84.34 | 21.05 | ^[13]^ |
| 2024 | 1.68 | 21.72 | 1.22 | 82.88 | 21.48 | ^[14]^ |
| 2024 | 1.68 | 21.48 | 1.24 | 82.70 | 20.90 | ^[15]^ |
| 2024 | 1.68 | 20.18 | 1.20 | 20.51 | 82.04 | ^[16]^ |
| 2023 | 1.68 | 21.44 | 1.19 | 83.5 | 21.66 | ^[17]^ |
| This work | 1.68 | 22.19 | 1.23 | 84.00 | 21.55 |  |

# Reference:

1. J. Zhang, T. Lu, *Phys. Chem. Chem. Phys.* **2021**, *23*, 20323-20328.
2. T. Lu, *J. Chem. Phys.* **2024**, *161*, 082503.
3. T. Lu, F. Chen, *J. Comput. Chem.* **2011**, *33*, 580-592.
4. Kresse, Furthmuller, *Phys. Rev.* **1996**, *54*, 11169.
5. n. Perdew, n. Burke, n. Ernzerhof, *Phys. Rev. Lett.* **1996**, *77*, 3865.
6. S. Grimme, J. Antony, S. Ehrlich, H. Krieg, *J. Chem. Phys.* **2010**, *132*, 154104.
7. X. Hu, W. Duan, K. Chen, B. Pi, S. Li, Z. Lin, C. Liu, Y. Pan, H. Ling, D. Li, L. Zhou, T. Liu, F. Wu, X. Guo, B. Zou, *Energy Environ. Sci.* **2025**, DOI: 10.1039/d5ee02269j.
8. L. Liu, Z. Ying, X. Li, H. Du, M. Zhang, J. Wu, Y. Sun, H. Ma, Z. He, Y. Yu, X. Guo, J. Sun, Y. Zeng, X. Yang, J. Ye, *Adv. Energy Mater.* **2025**, *15*, 2405675.
9. Q. Cao, T. Wang, X. Pu, X. He, M. Xiao, H. Chen, L. Zhuang, Q. Wei, H. L. Loi, P. Guo, B. Kang, G. Feng, J. Zhuang, G. Feng, X. Li, F. Yan, *Adv. Mater.* **2024**, *36*, 2311970.
10. C. Li, Y. Chen, Y. Li, L. Gong, Z. Yuan, L. Liang, J. Chen, P. Ganesan, Y. Zhang, J. Ma, P. Gao, *Angew. Chem. Int. Ed.* **2024**, *64*, e202420585.
11. T. Nie, Z. Fang, T. Yang, K. Zhao, J. Ding, S. Liu, *Angew. Chem. Int. Ed.* **2024**, *63*, e202400205.
12. L. Yang, Z. Fang, Y. Jin, H. Feng, B. Deng, L. Zheng, P. Xu, J. Chen, X. Chen, Y. Zhou, C. Shi, W. Gao, J. Yang, X. Xu, C. Tian, L. Xie, Z. Wei, *Adv. Mater.* **2024**, *36*, 2309768.
13. P. Jia, G. Chen, G. Li, J. Liang, H. Guan, C. Wang, D. Pu, Y. Ge, X. Hu, H. Cui, S. Du, C. Liang, J. Liao, G. Xing, W. Ke, G. Fang, *Adv. Mater.* **2024**, *36*, 2400105.
14. X. Li, Y. Li, Y. Feng, J. Qi, J. Shen, G. Shi, S. Yang, M. Yuan, T. He, *Adv. Mater.* **2024**, *36*, 2401103.
15. X. Hu, F. Yao, C. Wang, H. Cui, P. Jia, S. Du, S. Zhou, H. Guan, Q. Lin, W. Ke, C. Tao, G. Fang, *Chem. Eng. J.* **2024**, *489*, 151379.
16. J. Shen, N. Li, Y. Wang, X. Ge, J. Tao, S. Yin, X. Ning, T. He, G. Fu, S. Yang, *Chem. Eng. J.* **2024**, *489*, 151459.
17. X. Niu, N. Li, Z. Cui, L. Li, F. Pei, Y. Lan, Q. Song, Y. Du, J. Dou, Z. Bao, L. Wang, H. Liu, K. Li, X. Zhang, Z. Huang, L. Wang, W. Zhou, G. Yuan, Y. Chen, H. Zhou, C. Zhu, G. Liu, Y. Bai, Q. Chen, *Adv. Mater.* **2023**, *35*, 2305822.
